# Use of multiple epinephrine doses in anaphylaxis: A systematic review and meta-analysis

**DOI:** 10.1016/j.jaci.2021.03.042

**Published:** 2021-11

**Authors:** Nandinee Patel, Kok Wee Chong, Alexander Y.G. Yip, Despo Ierodiakonou, Joan Bartra, Robert J. Boyle, Paul J. Turner

**Affiliations:** a National Heart and Lung Institute, Imperial College London, London, United Kingdom; b Allergy Service, Department of Paediatric Medicine, KK Women’s and Children’s Hospital, Singapore; c School of Medicine, Imperial College London, London, United Kingdom; d Department of Primary Care and Population Health, University of Nicosia Medical School, Nicosia, Cyprus; e Hospital Clínic de Barcelona, Barcelona, Spain

**Keywords:** Epinephrine, allergic reaction, anaphylaxis, autoinjector device, refractory anaphylaxis, EAI, Epinephrine autoinjector, FAAN, Food Allergy and Anaphylaxis Network, IPD, Individual patient data, NIAID, National Institute of Allergy and Infectious Diseases

## Abstract

**Background:**

Regulatory bodies recommend that all patients at risk of anaphylaxis be prescribed 2 epinephrine autoinjectors, which they should carry at all times. This is in contrast to some guidelines. The proportion of anaphylaxis reactions that are treated with multiple doses of epinephrine has not been systematically evaluated.

**Objective:**

Our aim was to undertake a systematic review and meta-analysis of published studies reporting epinephrine treatment for anaphylaxis in which data relating to the number of doses administered were available.

**Methods:**

We searched the Medline, Embase, and Cochrane databases for relevant studies reporting at least 10 anaphylaxis events (due to food or venom) from 1946 until January 2020. Data were extracted in duplicate for the meta-analysis, and the risk of bias was assessed. The study was registered under the PROSPERO identifier CRD42017069109.

**Results:**

A total of 86 studies (36,557 anaphylaxis events) met the inclusion criteria (20 of the studies [23%] were prospective studies; 64 [74%] reported reactions in the community, and 22 [26%] included food challenge data). Risk of bias was assessed as low in 50 studies. Overall, 7.7% of anaphylaxis events from any cause (95% CI = 6.4-9.1) were treated with multiple doses of epinephrine. When only epinephrine-treated reactions for which subsequent doses were administered by a health care professional were considered, 11.1% of food-induced reactions (95% CI = 9.4-13.2) and 17.1% of venom-induced reactions (95% CI = 11.3-25.0) were treated with more than 1 epinephrine dose. Heterogeneity was moderate to high in the meta-analyses, but at sensitivity analysis this estimate was not affected by study design or anaphylaxis definition.

**Conclusion:**

Around 1 in 10 anaphylaxis reactions are treated with more than 1 dose of epinephrine.

Epinephrine is established as the first-line treatment for anaphylaxis.^1^ The majority of allergic reactions occur in the community.^2^ Delayed administration of epinephrine has been associated with poor outcomes in anaphylaxis.^3^^,^^4^ To mitigate against this, patients at risk of anaphylaxis to food and insect stings are often prescribed epinephrine autoinjectors (EAIs) for self-administration.

National and international regulatory agencies, including the US Food and Drug Administration, the Medicines and Healthcare Products Regulatory Agency in the United Kingdom, and the European Medicines Agency recommend that individuals at risk of anaphylaxis carry at least 2 EAIs at all times.^5^ This is in contrast to guidelines from some specialist societies, which make this recommendation for only selected “at-risk” patients.^6^, ^7^, ^8^, ^9^ This divergence in advice is potentially problematic for clinicians, who might be faced with medicolegal consequences if they go against official recommendations from regulatory authorities and prescribe only a single EAI device.

A number of observational studies have assessed the frequency of anaphylaxis reactions that fail to adequately respond to a single dose of epinephrine.^2^^,^^6^^,^^10^, ^11^, ^12^, ^13^, ^14^, ^15^ However, the data are limited by the studies' small sample sizes and differences in local practice in defining and treating anaphylaxis and heterogeneity in study design. As a result, estimates for the rate of allergic reactions treated with more than a single dose of epinephrine vary widely, ranging from 0%^16^ to 32%.^6^ We therefore undertook a systematic review and meta-analysis to assess the proportion of anaphylaxis reactions reported in the literature that were treated with at least 2 doses of epinephrine.

## Methods

This systematic review was registered at inception with PROSPERO (identifier CRD42017069109). The study is reported in accordance with Preferred Reporting Items for Systematic Reviews and Meta-Analyses Statement 2009 and Meta-analysis of Observational Studies in Epidemiology recommendations.^17^^,^^18^

### Search strategy and eligibility/inclusion criteria

We searched Medline, Embase, and the Cochrane Register of Controlled Trials, including all primary records from 1946 to July 2019 that referred to anaphylaxis in response to food or venom triggers, which included data with respect to the use of epinephrine (for search strategies and terms, see Tables E1-E3 in this article's Online Repository at www.jacionline.org). The search was updated in January 2021 by using the same methodology to include relevant studies published between July 2019 and December 2020. Eligible studies included those reporting more than 10 cases of anaphylaxis (by any definition) in individuals of all ages and in any country; the requirement for at least 10 cases was to minimize selection bias. We included both prospective and retrospective data, including data from food challenges conducted under medical supervision and patient surveys in which the categorization of anaphylaxis was evaluated by a health care professional (for further details, see the Methods section of the Online Repository at www.jacionline.org). No language restrictions were made, and we planned to include non-English articles if they met our inclusion criteria. We excluded data relating to adverse events following immunotherapy, as well as data sets that reported fatal anaphylaxis exclusively. Abstracts were independently screened by 2 researchers, and disagreements were resolved by discussion with a third team member. We also reviewed reference lists of included studies and review articles to identify other relevant studies. In cases in which potentially eligible studies did not report the number of epinephrine doses given, those studies' authors were contacted to determine whether these additional data could be provided.

### Data extraction and additional data

Data were extracted in duplicate (by J.B., K.W.C., N.P., and A.Y.), and any discrepancies identified were resolved by discussion and consensus with a third reviewer (P.J.T.). When needed, authors were contacted for clarifications. The screening process was undertaken using Endnote X8. For all studies, we extracted data relating to the proportion of study-defined anaphylaxis treated with more than a single dose of epinephrine, and we noted whether the definition used was that published by the National Institutes of Allergy and Infectious Diseases (NIAID)/Food Allergy and Anaphylaxis Network (FAAN).^19^ Authors were asked to provide further data to determine the proportion of reactions that involved objective cardiovascular and/or lower respiratory signs (which we termed *cardiorespiratory anaphylaxis*). We also extracted data with respect to the number of epinephrine-treated reactions as the denominator, given that anaphylaxis is frequently not treated with epinephrine and conversely, some nonanaphylaxis reactions are treated with epinephrine.^6^ We also noted whether epinephrine doses were administered by a health care professional to facilitate sensitivity analyses. Risk of bias was assessed in duplicate (by N.P. and K.W.C.) using the approach of Hoy et al.^20^

### Data analysis and statistical methods

Meta-analysis of proportions (Meta Package, R project, version 4.0.3) was undertaken by using an inverse variance method for summary estimates of logit-transformed data in a random effects model, with a continuity correction of 0.5 for studies with zero events (Clopper-Pearson for CIs and restricted maximum likelihood estimator for heterogeneity estimates). In cases in which substantial heterogeneity existed, meta-regression of categoric and continuous variables was performed to assess for potential moderators (eg, publication year). For meta-analyses of at least 10 studies, tests for small-study effects were performed by using funnel plots to assess asymmetry and Egger tests (with use of weighted linear regression of the outcome on its SE).

We undertook the following prespecified subgroup analyses: by trigger (community reactions to food, supervised food challenge, and venom); patient age (adult, child younger than 18 years, or both). Sensitivity analyses were undertaken to assess how estimates varied according to the following: use of different definitions of anaphylaxis (study-defined anaphylaxis, reactions with cardiorespiratory signs, or reactions with any use of injected epinephrine); inclusion of only studies at low risk of bias, full-text publications only, and publication after 2006 (when the NIAID/FAAN clinical criteria for anaphylaxis were published)^19^; and studies in which subsequent epinephrine doses were given by a health care professional (presumably on the basis of a suboptimal response to the initial epinephrine dose).

## Results

### Included studies and study/reaction characteristics

**Fig 1:**
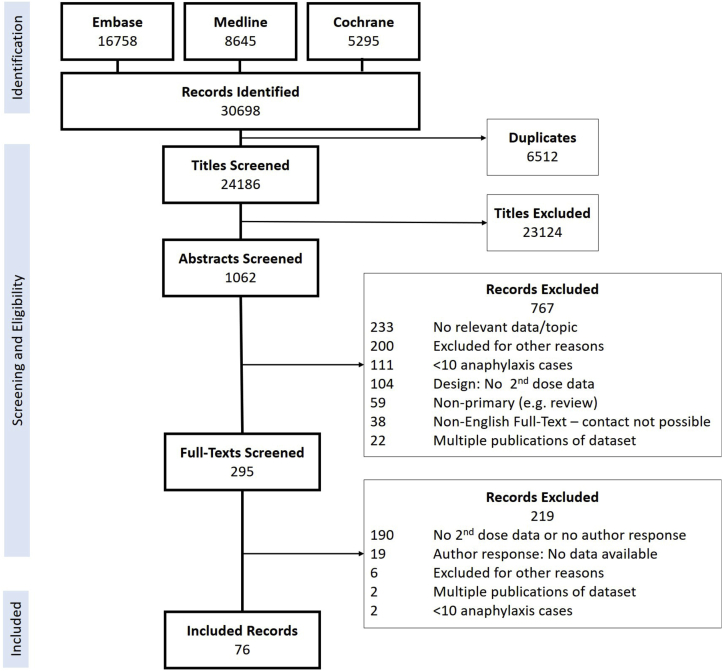
Preferred Reporting Items for Systematic Reviews and Meta-Analyses flow diagram.

The Preferred Reporting Items for Systematic Reviews and Meta-Analyses diagram for this systematic review is shown in Fig 1. A total of 86 studies were eligible for inclusion (76 from the original search and a further 10 from when the search was updated in 2021),^2^^,^^3^^,^^10^, ^11^, ^12^, ^13^, ^14^, ^15^, ^16^^,^^21^, ^22^, ^23^, ^24^, ^25^, ^26^, ^27^, ^28^, ^29^, ^30^, ^31^, ^32^, ^33^, ^34^, ^35^, ^36^, ^37^, ^38^, ^39^, ^40^, ^41^, ^42^, ^43^, ^44^, ^45^, ^46^, ^47^, ^48^, ^49^, ^50^, ^51^, ^52^, ^53^, ^54^, ^55^, ^56^, ^57^, ^58^, ^59^, ^60^, ^61^, ^62^, ^63^, ^64^, ^65^, ^66^, ^67^, ^68^, ^69^, ^70^, ^71^, ^72^, ^73^, ^74^, ^75^, ^76^, ^77^, ^78^, ^79^, ^80^, ^81^, ^82^, ^83^, ^84^, ^85^, ^86^, ^87^, ^88^, ^89^, ^90^, ^91^, ^92^, ^93^, ^94^, ^95^, ^96^, ^97^ representing 88 data sets (2 studies reported both retrospective and prospective data sets in the same publication)^14^^,^^16^ and a total of 36,557 anaphylaxis events (see Tables E13 and E14). A total of 35 studies reported food-induced reactions only, whereas 1 study reported venom-induced reactions only (see Tables E4 and E5). Of the remaining 50 studies, trigger-specific data were available for 23. Risk of bias and individual study characteristics are reported in Tables E6 and E15, respectively (available in the Online Repository at www.jacionline.org) and summarized in Table I. Of the 86 studies, 47 (55%) used the NIAID/FAAN criteria for anaphylaxis. Overall, epinephrine was administered in 50.4% of reactions (range 11.1%-100% across studies).

**Table I:** Summary of the included studies

Indicator	Any trigger	Food only	Venom only
Data sets available for meta-analysis (no.)			
Studies included	86	58	20
Available data sets	88	60	20
Data reports accidental reactions in the community	66	38	20
Study design (no.)			
Prospective	20/88	18/60	6/20
Retrospective	66/88	42/60	14/20
Both	2/88	0/60	0/20
Continent of study (no.)			
Europe	27	21	8
United States/Canada	38	24	8
Australia	12	8	3
Asia	11	7	1
Patient characteristics (no.)			
Children aged <18 years only	51	37	6
Adults only	3	2	0
Includes children and adults	34	21	14
Risk of bias (no.)			
Low	50	31	13
Moderate	36	27	7
High	2	2	0

### Rate of anaphylaxis reactions treated with more than 1 dose of epinephrine

**Fig 2:**
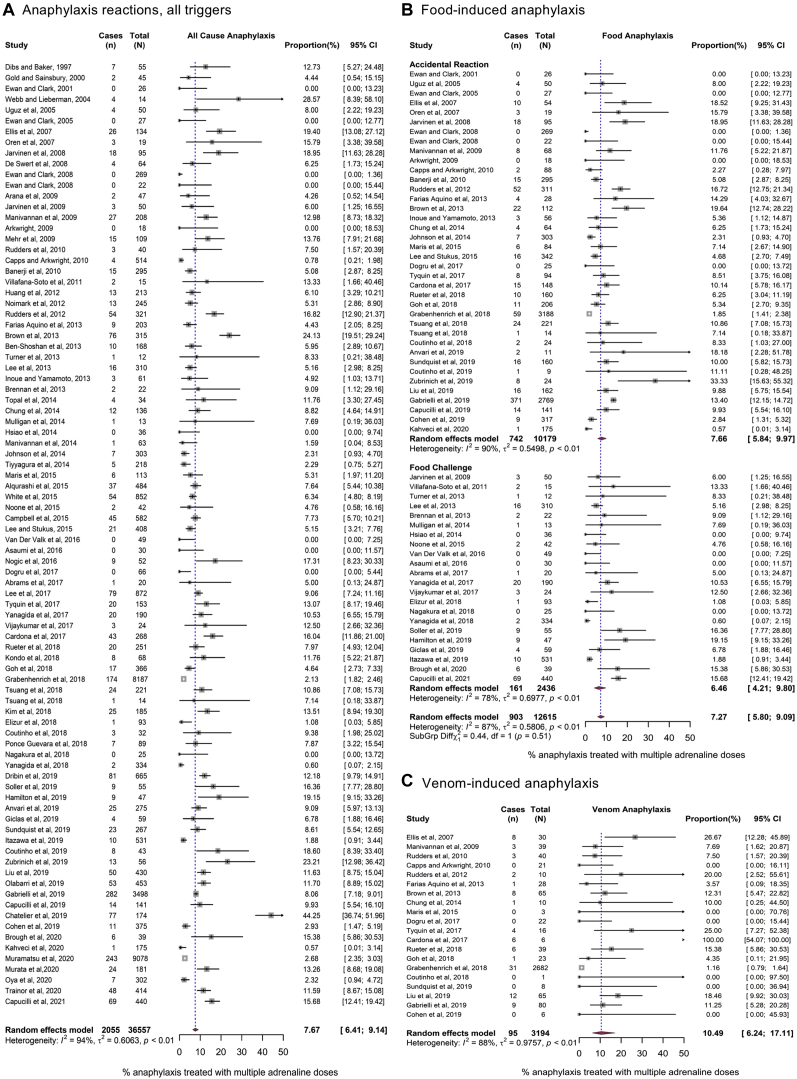
Forest plots for the use of 2 (or more) doses of epinephrine to treat allergic reactions. **A**, All triggers. **B**, Food-induced reactions. **C**, Venom-induced reactions.

**Fig 3:**
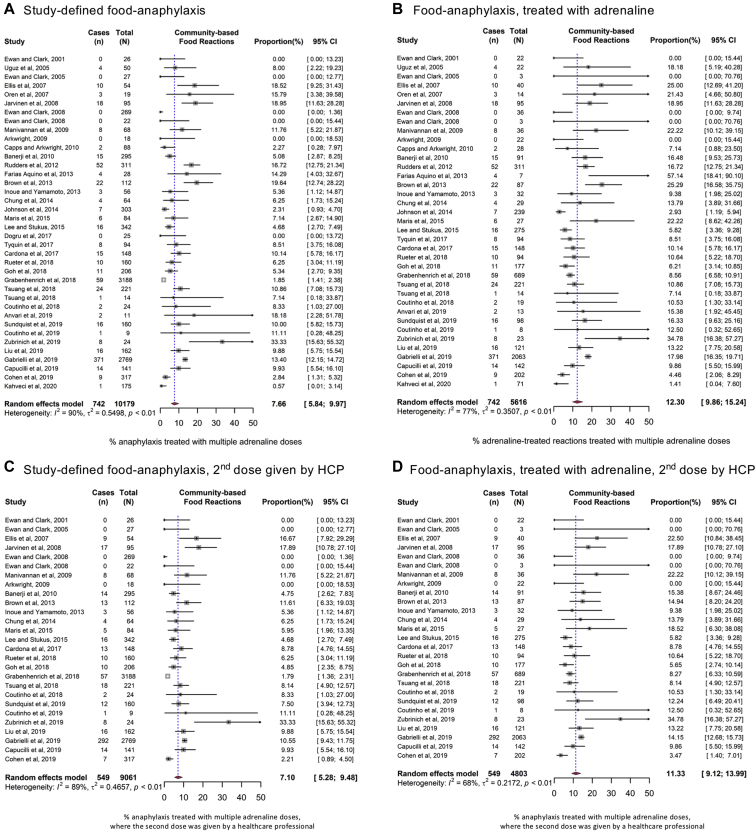
Forest plots for the use of 2 (or more) doses of epinephrine to treat food-related anaphylaxis occurring in the community as a result of accidental exposure. Study-defined anaphylaxis (**A**) and epinephrine-treated reactions only (**B**), irrespective of who administered the second (and subsequent) dose of epinephrine. Study-defined anaphylaxis (**C**), and only those epinephrine-treated reactions in which a subsequent dose of epinephrine was administered by a health care professional (HCP) (**D**).

Overall, at meta-analysis, 7.7% (95% CI = 6.4-9.1) of anaphylaxis reactions (all triggers) were treated with more than a single dose of epinephrine (Fig 2). We undertook sensitivity analyses to further refine this pooled estimate by limiting the definition of anaphylaxis to those reactions with objective cardiovascular or lower respiratory symptoms only (cardiorespiratory anaphylaxis) and those reactions for which epinephrine was administered. These estimates are reported in Table II (the corresponding forest plots are shown in Figs 2 and 3, and see also Figs E1-E18 [in the Online Repository at www.jacionline.org]). A slightly higher proportion of reactions (9.8% [95% CI = 7.8-12.2]) were treated with more than 1 dose of epinephrine when only cardiorespiratory anaphylaxis was considered. We also performed a separate analysis limiting the numerator to include only those reactions for which any subsequent doses were given by a health care professional (on the basis that such doses would be given only if there was a suboptimal response to the first dose of epinephrine). In this analysis, the pooled estimates of anaphylaxis reactions treated with further doses of epinephrine by a health care professional were 7.0% (95% CI = 5.5%-8.9%) for food-induced reactions and 10.0% (95% CI = 5.1%-18.8%) for venom-induced reactions. For food reactions, the rate of subsequent administration of epinephrine was higher in cases of reactions resulting from allergen exposure in the community than in cases of anaphylaxis occurring at food challenge performed under medical supervision, but this difference was not statistically significant.

**Table II:** Summary of pooled estimates for all meta-analyses undertaken, by definition of anaphylaxis used

Indicator	Trigger, % of reactions treated with >1 dose of epinephrine					
	All		Food			Venom
	Any setting (n = 36,557)	Community (n = 34,121)	Any setting (n = 12,615)	Community (n = 10,179)	Food challenge (n = 2,436)	Any setting (n = 3,194)
Study-defined anaphylaxis	7.7% (6.4%-9.1%)	7.9% (6.5%-9.7%)	7.3% (5.8%-9.1%)	7.7% (6.5%-10.0%)	6.5% (4.2%-9.8%)	10.5% (6.2%-17.1%)
Cardiorespiratory anaphylaxis	9.8% (7.8%-12.2%)	9.6% (7.6%-12.1%)	9.7% (7.0%-13.4%)	9.1% (6.2%-13.1%)	10.8% (6.0%-18.8%)	11.1% (4.3%-26.0%)
Reaction treated with ≥1 dose of epinephrine	12.9% (11.2%-14.9%)	13.5% (11.5%-15.9%)	11.7% (9.9%-13.9%)	12.3% (9.9%-15.2%)	10.6% (7.9%-14.1%)	17.9% (13.2%-24.0%)
Reaction for which further epinephrine was administered by a health care professional	7.1% (5.8%-8.7%)	7.2% (5.7%-9.0%)	7.0% (5.5%-8.9%)	7.1% (5.3%-9.5%)	6.8% (4.4%-10.4%)	10.0% (5.1%-18.8%)
Epinephrine-treated reaction for which further epinephrine was administered by a health care professional	12.2% (10.4%-14.3%)	12.8% (10.6%-15.4%)	11.1% (9.4%-13.2%)	11.4% (9.1%-14.0%)	10.8% (8.0%-14.4%)	17.1% (11.3%-25.0%)

Further subgrouping of meta-analyses by reaction trigger (any trigger, food, or venom) and setting (community reactions, food challenges under medical supervision, or any setting) are listed. Data are presented as percentages (pooled estimates [95% CI]).

We also undertook sensitivity analyses assessing the impact of study design, risk of bias, publication after 2006, or full-text publications only (see Tables E9-E12 in the Online Repository). The only significant difference (*P* < .001) identified was for the comparison of prospective versus retrospective studies when subsequent doses were administered by a health care professional: in the prospective studies, an estimated 5.1% of anaphylaxis reactions (95% CI = 2.9-8.9%) were treated with more than 1 dose of epinephrine administered by a health care professional, whereas in the retrospective studies, the corresponding rate was 7.9% (95% CI = 6.5-9.7%) (see Table E9).

### Heterogeneity and moderator assessment

Heterogeneity (as represented by *I*^*2*^) was moderate to high for all meta-analyses (range 51.7%-99.5%). Assessment of the contribution of potential moderators to the overall heterogeneity for the all-trigger and food trigger data sets was undertaken by using prespecified variables, including age group, study design (prospective vs retrospective), and publication year. No evidence of a moderator effect was noted. We also explored the impact of study size on the pooled estimates. Funnel plots for all meta-analyses are provided in Fig E19 (in the Online Repository at www.jacionline.org). Mild asymmetry was noted for smaller studies, with a relative absence of small studies demonstrating higher proportions of multiple epinephrine use. Egger tests were performed for all of the meta-analyses (see Table E8 in the Online Repository at www.jacionline.org); we did not identify any statistical evidence of small-study effects in the various meta-analyses undertaken, with the exception of venom- and food-induced reactions irrespective of who administered the second epinephrine dose (when limited to second doses given by a health care professional, the small-study effect was not apparent). The risk of bias was low in 56%, 52%, and 65% of studies contributing to all-trigger, food-trigger, and venom-trigger meta-analyses, respectively.

### Administration of 3 or more doses of epinephrine

A total of 11 studies reported the precise number of epinephrine doses administered. Overall, at least 3 doses were administered in 2.2% of anaphylaxis reactions (95% CI = 1.1%-4.1%) or in 3.4% of reactions treated with epinephrine (Table III^10^^,^^13^^,^^14^^,^^21^^,^^22^^,^^40^^,^^48^^,^^72^^,^^76^^,^^84^^,^^87^).

**Table III:** Total number of epinephrine doses given to individual patients receiving multiple doses of epinephrine

Study	% with food trigger	Proportion of anaphylaxis reactions treated with epinephrine				Proportion of epinephrine-treated reactions treated with multiple doses of epinephrine		
		1 Dose (%)	2 Doses (%)	3 Doses (%)	≥4 Doses (%)	2 Doses (%)	3 Doses (%)	≥4 Doses (%)
Järvinen et al, 2008^49^	100%	77/95 (81%)	12/95 (13%)	6/95 (6.3%)	0/95 (0%)	12/95 (13%)	6/95 (6.3%)	0/95 (0%)
Manivannan et al, 2009^10^	33%	77/208 (37%)	25/208 (12%)	2/208 (1.0%)	0/208 (0%)	25/104 (24%)	2/104 (1.9%)	0/104 (0%)
Noimark et al, 2012^76^	91%	28/245 (11%)	12/245 (4.9%)	1/245 (0.4%)	0/245 (0%)	12/41 (29%)	1/41 (2.4%)	0/41 (0%)
Brown et al, 2013^40^	36%	130/315 (54%)	59/315 (19%)	39/315 (12%)	17/315 (5.4%)	59/245 (24%)	39/245 (16%)	17/245 (6.9%)
Campbell et al, 2015^13^	36%	281/582 (48%)	36/582 (6.2%)	6/582 (1.0%)	3/582 (0.5%)	36/326 (11%)	6/326 (1.8%)	3/326 (0.9%)
Nogic et al, 2016^72^	75%	38/52 (73%)	10/52 (19%)	1/52 (1.9%)	0/52 (0%)	10/49 (20%)	1/49 (2.0%)	0/49 (0%)
Yanagida et al, 2017^22^	100%	70/190 (37%)	18/190 (9.5%)	2/190 (1.1%)	0/190 (0%)	18/90 (20%)	2/90 (2.2%)	0/90 (0%)
Tsuang et al, 2018^14^	100%	197/221 (89%)	19/221 (8.6%)	4/221 (1.8%)	1/221 (0.5%)	19/221 (8.6%)	4/221 (1.8%)	1/221 (0.5%)
Anvari et al, 2019^21^	48%	218/275 (79%)	20/275 (7.3%)	5/275∗ (1.8%)	—	20/243 (8.2%)	5/243∗ (2.1%)	—
Gabrielli et al, 2019^87^	79%	2276/3498 (65%)	234/3498 (6.7%)	36/3498 (1.0%)	12/3498 (0.3%)	234/2558 (9.1%)	36/2558 (1.4%)	12/2558 (0.5%)
Liu et al, 2019^84^	38%	255/430 (59%)	34/430 (7.9%)	16/430∗ (3.7%)	—	34/305 (11%)	16/305∗ (5.2%)	—
Pooled estimate at meta-analysis (95% CI)		57% (41%-72%)	9.2% (7.2%-12%)	2.2% (1.1%-4.1%)		14% (11%-19%)	3.4% (1.9%-5.9%)	
				1.9% (1.0%-3.5%)	0.3% (0.1%-1.3%)		2.9% (1.7%-5.0%)	0.5% (0.1%-1.9%)

These data were available in 11 studies. The pooled estimate for each dosing bracket is provided as a percentage of either the total number of study-defined anaphylaxis reactions or epinephrine-treated reactions (95% CI).

∗ Data available only for 3 or more doses.

## Discussion

This is the first systematic review in the literature in which meta-analysis was used to evaluate the rate of anaphylaxis reactions treated with more than 1 dose of epinephrine. We found that approximately 1 in 10 reactions are treated with at least 1 additional epinephrine dose. This estimate did not change significantly in the sensitivity analyses, including when the data were limited to those reactions for which subsequent doses were administered by a health care professional (which arguably might reflect a higher degree of confidence in the persistence of anaphylaxis symptoms despite initial treatment with epinephrine). This estimate was robust despite a high degree of heterogeneity between the included data sets, reflecting differences in cohort characteristics, study design and setting, and anaphylaxis definition used. The majority of the data sets assessed anaphylaxis occurring in the community; it is therefore likely that these data are representative of the broader population of individuals with allergy.

One potential limitation is that we were unable to distinguish between the administration route or dose of epinephrine given, as these data were not available for most data sets. However, the majority of data included was related to initial doses given in the community via use of EAIs. Excluding data sets published before 2006 (when the Joint Task Force on Practice Parameters published its recommendation that epinephrine be administered by the intramuscular route^98^) did not demonstrate any significant impact on the pooled estimates. Furthermore, we did not find that year of publication was a significant moderator in heterogeneity across studies. Several data sets reported biphasic reactions; unfortunately, we were unable to clarify with study authors whether the data provided with respect to epinephrine administration was for the first or delayed phase of these reactions. Thus, we were unable to assess the need for more than 1 dose of epinephrine to treat late-phase reactions.

A strength of this meta-analysis is the high response rate from authors who were contacted to provide further clarification. Many authors shared anonymized raw data, which facilitated the analyses. However, the meta-analyses were undertaken by using aggregate data from individual studies rather than individual patient data (IPD). Although this allowed for inclusion of a greater number of studies, we were unable to further assess potential risk factors for the use of multiple epinephrine doses, which would have been possible with an IPD meta-analysis. Given the inconsistencies in reported risk factors for multiple epinephrine use,^10^^,^^11^^,^^13^, ^14^, ^15^^,^^21^, ^22^, ^23^ an IPD meta-analysis would help address this evidence gap.

Recommendations vary with respect to the number of EAIs that patients at risk of anaphylaxis should be prescribed—both between countries and within a single country in which guidelines from specialist societies may contradict official government advice.^5^, ^6^, ^7^, ^8^, ^9^ Many anaphylaxis reactions resolve spontaneously without treatment,^2^^,^^76^ and in this analysis, we found that only 50.4% of anaphylaxis reactions were treated with *any* epinephrine, a rate that is consistent with the literature. It is clearly inappropriate to *not* treat anaphylaxis with epinephrine, which is rightly the universal recommendation in all international guidelines. A single dose of epinephrine may be insufficient to terminate a reaction for multiple reasons, including the following: reaction progression; underdosing (international guidelines recommend that teenagers and adults receive 0.5 mg of epinephrine, but for most EAI devices, the highest dose available is 0.3 mg of epinephrine); incorrect administration; subcutaneous administration, which is associated with a prolonged onset of action; delayed administration; and biphasic course of reaction. Our analysis, which is based on more than 25,000 anaphylaxis events, provides an important estimate of the frequency of multiple epinephrine doses given to treat anaphylaxis. Whether patients could be risk-stratified to assess the need for repeat doses of epinephrine requires further analysis, as is discussed in a recent publication by Shaker et al.^99^

### Conclusions

Around 10% of patients receiving epinephrine for anaphylaxis have a suboptimal response to a single dose of epinephrine, as assessed by a health care professional. These data are important in informing guidance on the provision of EAI for patients at risk of anaphylaxis in the community.

**Clinical implications**

Around 10% of anaphylaxis reactions are treated with more than 1 dose of epinephrine, including when the decision to administer a further dose was made by a health care professional.
