# Supplementary material for: Use of multiple epinephrine doses in anaphylaxis: A systematic review and meta-analysis
Source: J Allergy Clin Immunol. 2021 Nov;148(5):1307–15. doi: 10.1016/j.jaci.2021.03.042 (PMC8588837; doi:10.1016/j.jaci.2021.03.042)
Supplement: Supplementary Material [file mmc1.pdf]

ONLINE SUPPLEMENT

Use of multiple epinephrine doses in anaphylaxis: A systematic review and meta-analysis

Nandinee Patel,[1] Kok Wee Chong,[2] Alexander Y.G. Yip,[3] Despo Ierodiakonou,[4] Joan Bartra,[5] Robert J. Boyle,[1] Paul J Turner[1]

[1]National Heart & Lung Institute, Imperial College London, London, UK;

[2]Allergy Service, Department of Paediatric Medicine, KK Women's and Children's Hospital, Singapore;

[3]School of Medicine, Imperial College London, London, UK;

[4]Department of Primary Care and Population Health, University of Nicosia Medical School, Nicosia, Cyprus, Greece

[5]Hospital Clínic de Barcelona, Barcelona, Spain.

***Corresponding author:**

Dr Paul Turner

National Heart & Lung Institute,

Imperial College London,

Norfolk Place

London, W2 1PG

Email: p.turner@imperial.ac.uk

Contents

A. Additional Methods 4

Search Strategy and Eligibility/Inclusion Criteria 4

Table S1. Embase Search Strategy: 5

Table S2. Medline Search Strategy 6

Table S3. Cochrane Search Strategy 7

B. Number of Datasets and Events included in each Metanalysis 8

Table S4. Number of Studies and Number of Analysis in each data-set for each sub-analyses . 8

C. Breakdown of the percentage of reactions in each dataset by trigger (for ALL Cause Anaphylaxis analyses) 9

Table S5. Proportion of reactions (by aetiology) in each dataset 9

D. Risk of Bias Tables 11

Table S6: Risk of Bias assessment for all datasets (N=78) ED: emergency department, FC: food challenge, FT: full-text, ABS: Abstract 11

E. Additional Pooled Estimates and Forest Plots 14

Table S7: Pooled Estimates for all sub-analyses 14

Figure S1. Proportion of patients with allergic reactions who were diagnosed as having anaphylaxis: all trigger 15

Figure S2. Proportion of patients with allergic reactions who were diagnosed as having anaphylaxis: food 16

Figure S3. Proportion of patients with allergic reactions who were diagnosed as having anaphylaxis: venom 16

Figure S4. Proportion of anaphylaxis treated with multiple epinephrine doses: all trigger 17

Figure S5. Proportion of anaphylaxis treated with multiple epinephrine doses: food 18

Figure S6. Proportion of anaphylaxis treated with multiple epinephrine doses: venom 19

Figure S7. Cardiorespiratory reactions treated with multiple epinephrine doses: all triggers 20

Figure S8. Cardiorespiratory reactions treated with multiple epinephrine doses: food 21

Figure S9. Cardiorespiratory reactions treated with multiple epinephrine doses: venom 21

Figure S10. Epinephrine-treated reactions where multiple epinephrine doses were administered: all triggers 22

Figure S11. Epinephrine-treated reactions where multiple epinephrine doses were administered: food 23

Figure S12. Epinephrine-treated reactions where multiple epinephrine doses were administered: venom 24

Figure S13. Anaphylaxis reactions treated with multiple epinephrine doses by a health-care professional: all trigger 25

Figure S14. Anaphylaxis reactions treated with multiple epinephrine doses by a health-care professional: food 26

Figure S15. Anaphylaxis reactions treated with multiple epinephrine doses by a health-care professional: venom ... 26

Figure S16. Epinephrine-treated reactions with multiple epinephrine doses administered by a health-care professional: all triggers ... 27

Figure S17. Epinephrine-treated reactions with multiple epinephrine doses administered by a health-care professional: food ... 28

Figure S18. Epinephrine-treated reactions with multiple epinephrine doses administered by a health-care professional: venom ... 28

F. Funnel Plots and Egger Tests ... 29

Figure S19: Funnel Plots for all metanalyses: Assessment of small studies effect ... 29

Table S8: Egger Test: Intercept and p value ... 31

G. Summary of Pooled Estimates for Sensitivity Analyses ... 32

Table S9. Pooled estimates for prospective studies only. ... 32

Table S10. Pooled estimates for low risk of bias studies only. ... 32

Table S11. Pooled estimates for studies published after 2006 only. ... 33

Table S12. Pooled estimates for Full-Text references only. ... 33

H. Record Flow: Excluded Records at Full-Text screening ... 34

Table S13: References excluded at full-text screening with reason for exclusion provided ... 34

I. Record Flow: Included References ... 50

Table S14 ... 50

J. Summary of Data Extraction for Included Studies ... 56

Table S15. Individual study characteristics for included references ... 56

A. Additional Methods

Search Strategy and Eligibility/Inclusion Criteria

We searched MEDLINE, EMBASE, Cochrane Controlled Register of Trials, from launch to July 2019 for primary records referring to anaphylaxis reactions to food or venom triggers with data on the use of epinephrine. The full list of search terms for individual search databases with the records noted at each stage are listed in Table S1-S3. The search terms and strategies were reviewed by Imperial College London university library services prior to being run. The search was updated on 18 Jan 2021.

Reference lists were checked for additional publications where relevant. All primary data-records were included where at least 10 cases of anaphylaxis were reported. Where abstracts from peer-reviewed journals met eligibility criteria, data were included. Original studies from case series, registries, food challenge datasets and surveys (where health-professional review of the anaphylaxis diagnosis was apparent) were eligible. Reviews (non-original) data were excluded, although reference lists were cross-checked to include any relevant records not found via the search strategy. Where multiple publications were available with overlapping datasets, a single publication was included and authors were contacted to provide outcomes for the overall dataset where needed. Where extrapolative modelling had been performed on a sample dataset, studies were excluded unless complete data were also available on the sample, unmodelled data. Datasets exclusively focussed on cases of fatal anaphylaxis were excluded.

Participants/Population included:

Inclusion criteria: All age ranges, genders, ethnicities, country of study.

Exclusion criteria: nil

Intervention/Exposures

Inclusion criteria: We included studies where food or venom (hymenoptera) were listed (not exclusively) as known triggers in any case series. This included datasets where other triggers (eg. drug, unknown) were also included. We decided to focus on food and venom reactions as they form the key indications for epinephrine auto-injector prescription to patients.

Exclusion criteria: We excluded studies where there was no evidence that any food or venom anaphylaxis events were included in the study dataset. Datasets pertaining to immunotherapy-related reactions were excluded.

Outcomes

Data was collected on the following reference and study characteristics: publication year, country of study, duration of data collection, prospective/retrospective, study design and patient characteristics, definition of anaphylaxis used.

Data was extracted on the following study outcomes: By any trigger, food-only reactions, venom-only reactions): Numbers of allergic reactions, number of anaphylaxis reactions as defined by study authors, number of cardiorespiratory reactions, epinephrine use, use of multiple epinephrine doses, use of multiple epinephrine doses where any repeat doses were administered by a health-care professional, admissions to hospital, intensive care and fatalities and salbutamol use.

Table S1. Embase Search Strategy:

The initial search returned 16758 records. Updated search returned an additional 2230 records. The search terms are listed below.

1. exp food allergy/
2. food*.mp. [mp=title, abstract, heading word, drug trade name, original title, device manufacturer, drug manufacturer, device trade name, keyword, floating subheading word, candidate term word]
3. exp food/
4. 1 or 2 or 3
5. exp venom/
6. venom*.mp. [mp=title, abstract, heading word, drug trade name, original title, device manufacturer, drug manufacturer, device trade name, keyword, floating subheading word, candidate term word]
7. (bee or bees).mp. [mp=title, abstract, heading word, drug trade name, original title, device manufacturer, drug manufacturer, device trade name, keyword, floating subheading word, candidate term word]
8. Honeybee*.mp. [mp=title, abstract, heading word, drug trade name, original title, device manufacturer, drug manufacturer, device trade name, keyword, floating subheading word, candidate term word]
9. hymenoptera.mp. [mp=title, abstract, heading word, drug trade name, original title, device manufacturer, drug manufacturer, device trade name, keyword, floating subheading word, candidate term word]
10. (wasps or wasp).mp. [mp=title, abstract, heading word, drug trade name, original title, device manufacturer, drug manufacturer, device trade name, keyword, floating subheading word, candidate term word]
11. vespid.mp. [mp=title, abstract, heading word, drug trade name, original title, device manufacturer, drug manufacturer, device trade name, keyword, floating subheading word, candidate term word]
12. vespula.mp. [mp=title, abstract, heading word, drug trade name, original title, device manufacturer, drug manufacturer, device trade name, keyword, floating subheading word, candidate term word]
13. (ant or ants).mp. [mp=title, abstract, heading word, drug trade name, original title, device manufacturer, drug manufacturer, device trade name, keyword, floating subheading word, candidate term word]
14. 6 or 7 or 8 or 9 or 10 or 11 or 12 or 13
15. 4 or 14
16. exp anaphylaxis/
17. anaphyl*.mp. [mp=title, abstract, heading word, drug trade name, original title, device manufacturer, drug manufacturer, device trade name, keyword, floating subheading word, candidate term word]
18. 16 or 17
19. epinephrine/
20. epinephrine autoinjector/
21. adrenalin*.mp. [mp=title, abstract, heading word, drug trade name, original title, device manufacturer, drug manufacturer, device trade name, keyword, floating subheading word, candidate term word]
22. epinephrin*.mp. [mp=title, abstract, heading word, drug trade name, original title, device manufacturer, drug manufacturer, device trade name, keyword, floating subheading word, candidate term word]
23. epipen*.mp. [mp=title, abstract, heading word, drug trade name, original title, device manufacturer, drug manufacturer, device trade name, keyword, floating subheading word, candidate term word]
24. 19 or 20 or 21 or 22 or 23
25. 18 or 24
26. 25 and 15
27. (exp animal/ or nonhuman/) not exp human/
28. 26 not 27
29. limit 28 to ("systematic review" and "review")
30. 28 not 29

Table S2. Medline Search Strategy

The initial search returned 8645 records. Updated search returned an additional 996 records. The search terms are listed below.

1. exp Food Hypersensitivity/
2. food*.mp. [mp=title, abstract, heading word, drug trade name, original title, device manufacturer, drug manufacturer, device trade name, keyword, floating subheading word, candidate term word]
3. exp Food/
4. 1 or 2 or 3
5. exp Venoms/
6. venom*.mp. [mp=title, abstract, heading word, drug trade name, original title, device manufacturer, drug manufacturer, device trade name, keyword, floating subheading word, candidate term word]
7. exp Hymenoptera/
8. exp Arthropod Venoms/
9. (bee or bees).mp. [mp=title, abstract, heading word, drug trade name, original title, device manufacturer, drug manufacturer, device trade name, keyword, floating subheading word, candidate term word]
10. Honeybee*.mp. [mp=title, abstract, heading word, drug trade name, original title, device manufacturer, drug manufacturer, device trade name, keyword, floating subheading word, candidate term word]
11. hymenoptera.mp. [mp=title, abstract, heading word, drug trade name, original title, device manufacturer, drug manufacturer, device trade name, keyword, floating subheading word, candidate term word]
12. (wasps or wasp).mp. [mp=title, abstract, heading word, drug trade name, original title, device manufacturer, drug manufacturer, device trade name, keyword, floating subheading word, candidate term word]
13. vespid.mp. [mp=title, abstract, heading word, drug trade name, original title, device manufacturer, drug manufacturer, device trade name, keyword, floating subheading word, candidate term word]
14. vespula.mp. [mp=title, abstract, heading word, drug trade name, original title, device manufacturer, drug manufacturer, device trade name, keyword, floating subheading word, candidate term word]
15. (ant or ants).mp. [mp=title, abstract, heading word, drug trade name, original title, device manufacturer, drug manufacturer, device trade name, keyword, floating subheading word, candidate term word]
16. 5 or 6 or 7 or 8 or 9 or 10 or 11 or 12 or 13 or 14 or 15
17. 4 or 16
18. exp Anaphylaxis/
19. anaphyl*.mp. [mp=title, abstract, heading word, drug trade name, original title, device manufacturer, drug manufacturer, device trade name, keyword, floating subheading word, candidate term word]
20. 18 or 19
21. exp Epinephrine/
22. adrenalin*.mp. [mp=title, abstract, heading word, drug trade name, original title, device manufacturer, drug manufacturer, device trade name, keyword, floating subheading word, candidate term word]
23. epinephrin*.mp. [mp=title, abstract, heading word, drug trade name, original title, device manufacturer, drug manufacturer, device trade name, keyword, floating subheading word, candidate term word]
24. epipen*.mp. [mp=title, abstract, heading word, drug trade name, original title, device manufacturer, drug manufacturer, device trade name, keyword, floating subheading word, candidate term word]
25. 21 or 22 or 23 or 24
26. 20 or 25
27. 17 and 26
28. exp animals/ not humans.sh.
29. 27 not 28
30. limit 29 to ("systematic review" and "review")
31. 29 not 30

Table S3. Cochrane Search Strategy

The search terms and records returned in the initial and updated search are listed below.

"Adrenalin" in Title Abstract Keyword OR "adrenaline" in Title Abstract Keyword OR "anaphylaxis" in Title Abstract Keyword - with Cochrane Library publication date Between Jan 1900 and May 2019 (Word variations have been searched). Generates:			
Initial search:	34 Cochrane reviews	5260 trials	1 Cochrane protocol
Additional records in updated search:	5 Cochrane reviews	370 trials	0 Cochrane protocol

B. Number of Datasets and Events included in each Metanalysis

The number of datasets and number of allergic reactions (denominator) in each Metanalysis are listed below.

Table S4. Number of Studies and Number of Analysis in each data-set for each sub-analyses

	**Number of datasets in analysis**			**Number of reactions in analysis**		
	Accidental Reactions	Food Challenge	All Studies	Accidental Reactions	Food Challenge	All Studies
Proportion of patients with allergic reactions who were diagnosed as having anaphylaxis						
ALL TRIGGERS	30	19	49	26,578	7,164	33,742
FOOD TRIGGERS	14		33	3,499		10,663
VENOM TRIGGERS	3	-	3	284	-	284
Proportion of anaphylaxis treated with multiple epinephrine doses						
ALL TRIGGERS	66	22	88	34,121	2,436	36,557
FOOD TRIGGERS	38		60	10,179		12,615
VENOM TRIGGERS	20	-	20	3,194	-	3,194
Cardiorespiratory reactions treated with multiple epinephrine doses						
ALL TRIGGERS	49	16	65	16,960	1,469	18,429
FOOD TRIGGERS	20		36	4,397		5,866
VENOM TRIGGERS	10	-	10	2,128	-	2,128
Epinephrine-treated reactions where multiple epinephrine doses were administered						
ALL TRIGGERS	66	22	88	16,985	1,453	18,438
FOOD TRIGGERS	37		59	5,616		7,069
VENOM TRIGGERS	19	-	19	725	-	725
Anaphylaxis reactions treated with multiple epinephrine doses by a healthcare professional (HCP)						
ALL TRIGGERS	43	20	63	20,342	2,381	22,723
FOOD TRIGGERS	27		47	9,061		11,442
VENOM TRIGGERS	15	-	15	3,067	-	3,067
Epinephrine-treated reactions with multiple epinephrine doses administered by a HCP						
ALL TRIGGERS	43	20	63	9,499	1,438	10,937
FOOD TRIGGERS	27		47	4,803		6,241
VENOM TRIGGERS	15	-	15	666	-	666

C. Breakdown of the percentage of reactions in each dataset by trigger (for ALL Cause Anaphylaxis analyses)

For the ALL Trigger metanalyses, this table lists the percentage of total reactions caused by food, venom, drug or other triggers.

Table S5. Proportion of reactions (by aetiology) in each dataset

Reference	Percentage of food, venom, drug and other/unknown triggers contributing to each dataset within meta-analysis				N (all reactions)
	Food (%)	Venom (%)	Drug (%)	Unknown/ Other (%)	
Abrams et al, 2017	100	0	0	0	104
Alqurashi et al, 2015	70	2	2	26	484
Anvari S et al, 2019	48	11	12	29	275
Arana et al, 2009	81	-	-	19	47
Arkwright, 2009	100	0	0	0	22
Asaumi et al, 2016	100	0	0	0	30
Banerji et al, 2010	100	0	0	0	486
Ben-Shoshan et al, 2013	85	4	3	9	168
Brennan et al, 2013	100	0	0	0	22
Brough et al, 2020	100	0	0	0	238
Brown et al, 2013	36	21	-	-	315
Campbell et al, 2015	36	12	19	33	582
Capps and Arkwright, 2010	28	7	52	14	488
Capucilli et al, 2019	100	0	0	0	168
Capucilli et al, 2021	100	0	0	0	746
Cardona et al, 2017	55	2	32	10	268
Chatelier et al, 2019	52	-	-	48	174
Chung et al, 2014	47	7	16	29	136
Cohen et al, 2019	85	2	-	13	375
Coutinho et al, 2018	75	3	16	6	32
Coutinho et al, 2019	21	23	33	23	43
De Swert et al, 2008	75	6	8	11	64
Dibs and Baker, 1997	25	15	18	42	55
Dogru et al, 2017	38	33	21	0	66
Dribin et al, 2019	58	1	6	34	665
Elizur et al, 2018	100	0	0	0	93
Ellis et al, 2007	40	22	16	23	134
Ewan and Clark, 2001	100	0	0	0	26
Ewan and Clark, 2005	100	0	0	0	27
Ewan and Clark, 2008	100	0	0	0	269
Ewan and Clark, 2008	100	0	0	0	22
Farias Aquino et al, 2013	14	14	-	-	203
Gabrielli et al, 2019	79	2	5	13	3498
Giclas et al, 2019	100	0	0	0	70
Goh et al, 2018	56	6	-	37	366
Gold and Sainsbury, 2000	49	27	0	24	45
Grabenhenrich et al, 2018	39	33	18	10	8187
Hamilton et al, 2019	100	0	0	0	47
Hsiao et al, 2014	100	0	0	0	306
Huang et al, 2012	71	-	9	20	213

Inoue and Yamamoto, 2013	92	0	2	7	61
Itazawa et al, 2013	100	0	0	0	531
Jarvinen et al, 2008	100	0	0	0	95
Jarvinen et al, 2009	100	0	0	0	436
Johnson et al, 2014	100	0	0	0	703
Kahveci et al, 2020	100	0	0	0	175
Kim et al, 2018	27	10	56	7	194
Kondo et al, 2018	32	63	3	1	68
Lee and Stukus, 2015	84	0	4	12	408
Lee et al, 2013	100	0	0	0	310
Lee et al, 2017	35	12	25	27	872
Liu et al, 2019	38	15	19	28	430
Manivannan et al, 2009	33	19	14	35	208
Manivannan et al, 2014	28	12	31	29	103
Maris et al, 2015	74	3	6	17	113
Mehr et al, 2009	82	4	7	7	107
Mulligan et al, 2014	100	0	0	0	150
Muramatsu et al, 2020	21	-	-	79	9079
Murata et al,2020	-	-	-	-	271
Nagakura et al, 2018	100	0	0	0	25
Nogic et al, 2016	75	4	6	15	52
Noimark et al, 2012	91	0	2	6	245
Noone et al, 2015	100	0	0	0	74
Olabarri et al, 2019	-	-	-	-	453
Oren et al, 2007	100	0	0	0	19
Oya et al, 2020	76	-	15	9	302
Ponce Guevara et al, 2018	43	9	34	15	89
Rudders et al, 2010	0	100	0	0	153
Rudders et al, 2012	50	3		47	321
Rueter et al, 2018	64	16	3	18	251
Soller et al, 2019	100	0	0	0	55
Sundquist et al, 2019	60	3	21	16	267
Tiyyagura et al, 2014	76	1	8	16	218
Topal et al, 2014	18	18	62	3	34
Trainor et al, 2020	86	0.5	3	-	414
Tsuang et al, 2018	100	0	0	0	221
Tsuang et al, 2018	100	0	0	0	14
Turner et al, 2013	100	0	0	0	86
Tyquin et al, 2017	58	10	-	-	161
Uguz et al, 2005	100	0	0	0	126
Van Der Valk et al, 2016	100	0	0	0	137
Vijaykumar et al, 2017	100	0	0	0	24
Villafana-Soto et al, 2011	100	0	0	0	88
Webb and Lieberman, 2004	22	0	11	67	601
White et al, 2015	62	-	-	-	852
Yanagida et al, 2017	100	0	0	0	190
Yanagida et al, 2018	100	0	0	0	334
Zubrinich et al, 2019	44	0	29	0	48

D. Risk of Bias Tables

For all included references the table below lists the risk of bias assessment.

Table S6: Risk of Bias assessment for all datasets (N=78) ED: emergency department, FC: food challenge, FT: full-text, ABS: Abstract

Reference	Study Setting	Type of Study	Full Text or Abstract	Risk of Bias: Anaphylaxis Definition	Risk of Bias: Epinephrine Methodology	Risk of Bias: Completeness of Data	Risk of Bias: Representative data	Overall Risk of Bias Assessment
Abrams et al, 2017	ROUTINE FC	Food Challenge	FT	0	0	0	0	LOW
Alqurashi et al, 2015	ED	Acute.retro	FT	0	0	0	0	LOW
Anagnostou et al, 2019	ED	Acute.retro	FT	0	1	0	0	MODERATE
Arana et al, 2009	CLINIC CASE SERIES	Clinic	ABS	0	0	0	0	LOW
Arkwright, 2009	SURVEY	Survey	FT	0	0	0	1	MODERATE
Asaumi et al, 2016	ROUTINE FC	Food Challenge	FT	0	0	0	1	MODERATE
Banerji et al, 2010	ED	Acute.retro	ABS	0	1	0	0	MODERATE
Ben-Shoshan et al, 2013	ED	Acute.prosp	FT	0	0	0	0	LOW
Brennan et al, 2013	ROUTINE FC	Food Challenge	ABS	0	0	1	0	MODERATE
Brown et al, 2013	ED	Acute.prosp	FT	0	0	0	1	MODERATE
Campbell et al, 2015	ED	Acute.retro	FT	0	0	0	0	LOW
Capps and Arkwright, 2010	PREHOSP	Acute.retro	FT	0	0	1	0	MODERATE
Cardona et al, 2017	REGISTRY	Clinic	FT	0	0	0	1	MODERATE
Chung et al, 2014	PREHOSP	Acute.retro	FT	0	0	1	0	LOW
Coutinho et al, 2018	ED	Acute.retro	FT	0	0	1	0	MODERATE
Coutinho et al, 2019	ED	Acute.retro	FT	0	0	0	0	LOW
De Swert et al, 2008	CLINIC CASE SERIES	Clinic	FT	0	0	0	0	LOW
Dibs and Baker, 1997	CLINIC CASE SERIES	Acute.retro	FT	0	0	0	1	MODERATE
Dogru et al, 2017	CLINIC CASE SERIES	Clinic	FT	0	0	0	0	LOW
Dribin et al, 2019	ED	Acute.retro	FT	0	0	0	0	LOW
Elizur et al, 2018	RESEARCH FC	Food Challenge	FT	0	0	0	0	LOW
Ellis et al, 2007	ED	Acute.retro	FT	0	0	0	0	LOW
Ewan and Clark, 2001	CLINIC CASE SERIES	Clinic	FT	0	0	1	0	MODERATE
Ewan and Clark, 2005	CLINIC CASE SERIES	Clinic	FT	0	0	1	0	MODERATE
Ewan and Clark, 2008	CLINIC CASE SERIES	Clinic	FT	0	0	1	0	MODERATE
Ewan and Clark, 2008	CLINIC CASE SERIES	Clinic	FT	0	0	1	0	MODERATE
Farias Aquino et al, 2013	ED	Acute.retro	ABS	0	0	1	1	MODERATE
Giclas et al, 2019	ROUTINE FC	Food Challenge	ABS	0	0	0	0	LOW

Goh et al, 2018	ED	Acute.retro	FT	0	0	1	0	MODERATE
Gold and Sainsbury, 2000	SURVEY	Survey	FT	0	0	0	1	MODERATE
Grabenhenrich et al, 2018	REGISTRY	Clinic	FT	0	0	0	0	LOW
Hamilton et al, 2019	RESEARCH FC	Food Challenge	ABS	0	0	1	1	MODERATE
Hsiao et al, 2014	ROUTINE FC	Food Challenge	ABS	0	0	0	0	LOW
Huang et al, 2012	ED	Acute.retro	FT	0	0	0	0	LOW
Inoue and Yamamoto, 2013	CLINIC CASE SERIES	Acute.retro	FT	0	0	1	0	MODERATE
Itazawa et al, 2013	ROUTINE FC	Food Challenge	FT	0	0	0	0	LOW
Jarvinen et al, 2008	SURVEY	Survey	FT	1	1	0	0	MODERATE
Jarvinen et al, 2009	RESEARCH FC	Food Challenge	FT	0	0	0	0	LOW
Johnson et al, 2014	ED	Acute.retro	FT	0	0	0	0	LOW
Kim et al, 2018	ED	Acute.retro	FT	0	0	0	0	LOW
Kondo et al, 2018	PREHOSP	Acute.retro	FT	0	0	0	1	MODERATE
Lee and Stukus, 2015	ED+PREHOSP	Acute.retro	ABS	0	0	0	0	LOW
Lee et al, 2012	ROUTINE FC	Food Challenge	FT	0	0	0	0	LOW
Lee et al, 2017	ED	Acute.retro	FT	0	0	0	0	LOW
Liu et al, 2019	ED	Acute.prosp	FT	0	0	0	0	LOW
Manivannan et al, 2009	CLINIC CASE SERIES	Acute.retro	FT	0	0	0	0	LOW
Manivannan et al, 2014	PREHOSP	Acute.prosp	FT	0	0	0	0	LOW
Maris et al, 2015	REGISTRY	Acute.prosp	ABS	0	0	0	0	LOW
Mehr et al, 2009	ED	Acute.retro	FT	0	0	0	1	MODERATE
Mulligan et al, 2014	ROUTINE FC	Food Challenge	ABS	0	0	0	0	LOW
Nagakura et al, 2018	RESEARCH FC	Food Challenge	FT	0	0	0	1	MODERATE
Nogic et al, 2016	ED+PREHOSP	Acute.retro	FT	0	0	0	0	LOW
Noimark et al, 2012	SURVEY	Survey	FT	0	0	0	0	LOW
Noone et al, 2015	RESEARCH FC	Food Challenge	FT	1	0	0	1	MODERATE
Olabarri et al, 2019	ED	Acute.prosp	ABS	0	0	0	0	LOW
Oren et al, 2007	ED	Acute.retro	FT	0	0	0	1	MODERATE
Ponce Guevara et al, 2018	ED	Acute.retro	FT	0	0	0	0	LOW
Rudders et al, 2010	ED	Acute.retro	FT	0	0	0	0	LOW
Rudders et al, 2012	ED	Acute.retro	FT	0	0	1	1	MODERATE
Rueter et al, 2018	ED	Acute.retro	FT	0	0	0	0	LOW
Soller et al, 2019	ROUTINE FC	Food Challenge	FT	0	0	0	0	LOW
Sundquist et al, 2019	ED	Acute.retro	FT	0	0	0	0	LOW
Tiyyagura et al, 2014	ED	Acute.retro	FT	0	0	0	0	LOW

Topal et al, 2014	CLINIC CASE SERIES	Acute.retro	FT	1	0	0	1	MODERATE
Tsuang et al, 2018	CLINIC CASE SERIES	Clinic	FT	1	1	0	0	MODERATE
Tsuang et al, 2018	CLINIC CASE SERIES	Clinic	FT	1	0	1	1	HIGH
Turner et al, 2013	ROUTINE FC	Food Challenge	FT	0	0	0	1	MODERATE
Tyquin et al, 2017	CLINIC CASE SERIES	Clinic	ABS	0	0	0	0	LOW
Uguz et al, 2005	SURVEY	Survey	FT	1	0	1	1	HIGH
Van Der Valk et al, 2016	RESEARCH FC	Food Challenge	FT	0	0	0	0	LOW
Vijaykumar et al, 2017	ROUTINE FC	Food Challenge	ABS	1	0	0	0	MODERATE
Villafana-Soto et al, 2011	ROUTINE FC	Food Challenge	ABS	0	0	0	0	LOW
Webb and Lieberman, 2004	CLINIC CASE SERIES	Clinic	FT	1	0	0	1	MODERATE
White et al, 2015	SURVEY	Survey	ABS	1	0	0	0	MODERATE
Yanagida et al, 2017	RESEARCH FC	Food Challenge	FT	0	0	0	0	LOW
Yanagida et al, 2018	ROUTINE FC	Food Challenge	ABS	0	0	0	0	LOW
Zubrinich et al, 2019	ED	Acute.retro	FT	0	0	0	0	LOW
Brough et al, 2020	RESEARCH FC	Food Challenge	FT	1	0	0	1	MODERATE
Capucilli et al, 2019	ED	Acute.retro	FT	0	0	0	0	LOW
Capucilli et al, 2021	ROUTINE FC	Food Challenge	FT	0	0	0	0	LOW
Chatelier et al, 2019	ED	Acute.retro	ABS	0	0	0	0	LOW
Cohen et al, 2019	ED	Acute.retro	FT	0	0	0	0	LOW
Kahveci et al, 2020	ED	Acute.retro	FT	0	1	0	1	MODERATE
Muramatsu et al, 2020	ED	Acute.retro	FT	1	0	1	0	MODERATE
Murata et al,2020	ED	Acute.retro	FT	1	0	0	1	MODERATE
Oya et al, 2020	ED	Acute.prosp	FT	0	0	0	0	LOW
Trainor et al, 2020	ED	Acute.retro	FT	0	0	0	0	LOW

E. Additional Pooled Estimates and Forest Plots

Table S7: Pooled Estimates for all sub-analyses by trigger and anaphylaxis definition

Trigger	ALL CAUSE	FOOD	VENOM
**Proportion of patients with allergic reactions who were diagnosed as having anaphylaxis**			
Number of datasets (Overall estimate)	49	33	3
Accidental Reactions % Estimate (95%CI):	50.4 (37.8; 63.0)	48.2 (34.7; 62.0)	51.6 (25.8; 76.6)
Food Challenges % Estimate (95%CI):	31.8 (22.6; 42.7)	31.8 (22.6; 42.7)	-
Overall % Estimate (95%CI):	42.9 (34.0; 52.2)	38.5 (30.1; 47.6)	51.6 (25.8; 76.6)
Tau^2; I^2	1.7457; 99.5%	1.1480; 97.3%	0.9142; 95.2%
Subgroup Differences (Q value; p value)	4.85; 0.0276	3.43; 0.0639	-
Corresponding Figure for Forest Plot	Fig.S1	Fig.S2	Fig.S3
**Proportion of anaphylaxis treated with multiple epinephrine doses**			
Number of datasets (Overall estimate)	88	60	20
Accidental Reactions % Estimate (95%CI):	8.0 (6.5; 9.7)	7.7 (5.8; 10.0)	10.5 (6.2; 17.1)
Food Challenges % Estimate (95%CI):	6.5 (4.2; 9.8)	6.5 (4.2; 9.8)	-
Overall % Estimate (95%CI):	7.7 (6.4; 9.1)	7.3 (5.8; 9.1)	10.5 (6.2; 17.1)
Tau^2; I^2	0.6063; 93.6%	0.5806; 87.0%	0.9757; 88.3%
Subgroup Differences (Q value; p value)	0.78; 0.3757	0.44; 0.5062	-
Corresponding Figure for Forest Plot	Fig.S4	Fig.S5	Fig.S6
**Cardiorespiratory reactions treated with multiple epinephrine doses**			
Number of datasets (Overall estimate)	65	36	10
Accidental Reactions % Estimate (95%CI):	9.6 (7.6; 12.0)	9.1 (6.2; 13.1)	11.1 (4.3; 26.0)
Food Challenges % Estimate (95%CI):	10.8 (6.0; 18.8)	10.8 (6.0; 18.8)	-
Overall % Estimate (95%CI):	9.8 (7.8; 12.2)	9.8 (7.0; 13.4)	11.1 (4.3; 26.0)
Tau^2; I^2	0.7432; 93.7%	0.8067; 90.7%	2.0096; 89.9%
Subgroup Differences (Q value; p value)	0.14; 0.7055	0.25; 0.6172	-
Corresponding Figure for Forest Plot	Fig.S7	Fig.S8	Fig.S9
**Epinephrine-treated reactions treated with multiple epinephrine doses**			
Number of datasets (Overall estimate)	88	59	19
Accidental Reactions % Estimate (95%CI):	13.5 (11.5; 15.9)	12.3 (9.9; 15.2)	18.0 (13.2; 24.0)
Food Challenges % Estimate (95%CI):	10.6 (7.9; 14.1)	10.6 (7.9; 14.1)	-
Overall % Estimate (95%CI):	12.9 (11.2; 14.9)	11.7 (9.9; 13.9)	18.0 (13.2; 24.0)
Tau^2; I^2	0.3996; 88.1%	0.2999; 71.7%	0.2069; 51.7%
Subgroup Differences (Q value; p value)	2.06; 0.1515	0.62; 0.4316	-
Corresponding Figure for Forest Plot	Fig.S10	Fig.S11	Fig.S12
**Anaphylaxis reactions where multiple epinephrine doses were administered by a healthcare professional**			
Number of datasets (Overall estimate)	63	47	15
Accidental Reactions % Estimate (95%CI):	7.2 (5.7; 9.0)	7.1 (5.3; 9.5)	10.0 (5.1; 18.8)
Food Challenges % Estimate (95%CI):	6.8 (4.4; 10.4)	6.8 (4.4; 10.4)	-
Overall % Estimate (95%CI):	7.1 (5.8; 8.7)	7.0 (5.5; 8.9)	10.0 (5.1; 18.8)
Tau^2; I^2	0.5418; 91.1%	0.5265; 86.3%	1.2887; 90.3%
Subgroup Differences (Q value; p value)	0.05; 0.8205	0.03; 0.8743	-
Corresponding Figure for Forest Plot	Fig.S13	Fig.S14	Fig.S15
**Epinephrine-treated reactions with multiple epinephrine doses administered by a health-care professional**			
Number of datasets (Overall estimate)	63	47	15
Accidental Reactions % Estimate (95%CI):	12.8 (10.6; 15.4)	11.3 (9.1; 14.0)	17.1 (11.3; 25.0)
Food Challenges % Estimate (95%CI):	10.8 (8.0; 14.4)	10.8 (8.0; 14.4)	-
Overall % Estimate (95%CI):	12.2 (10.4; 14.3)	11.1 (9.4; 13.2)	17.1 (11.3; 25.0)
Tau^2; I^2	0.3254; 80.9%	0.2117; 62.9%	0.3284; 60.1%
Subgroup Differences (Q value; p value)	0.95; 0.3288	0.07; 0.7853	-
Corresponding Figure for Forest Plot	Fig.S16	Fig.S17	Fig.S18

Below are the corresponding forest plots for the meta-analyses listed in Table S7.

Figure S1. Proportion of patients with allergic reactions who were diagnosed as having anaphylaxis: all trigger

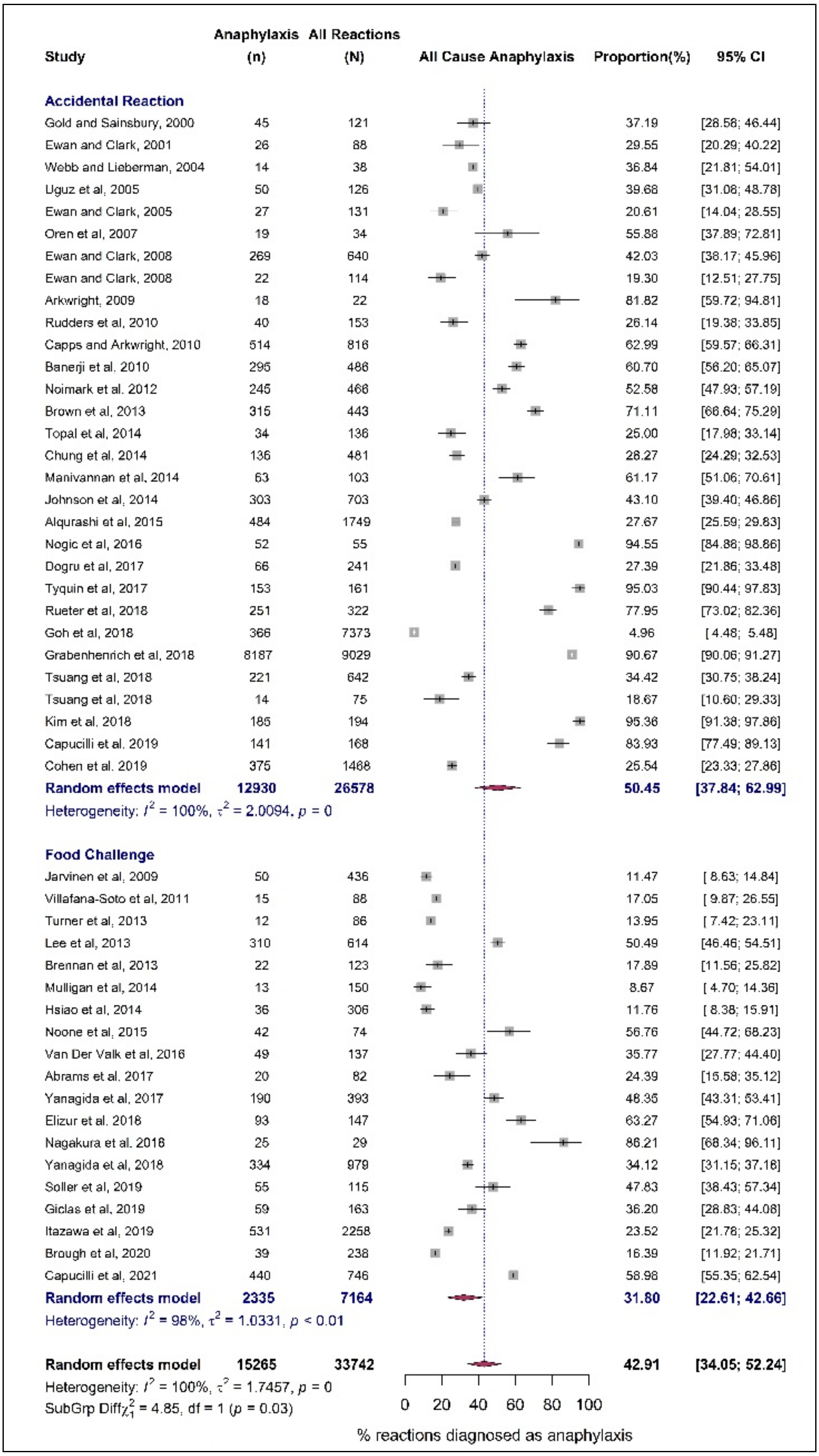

Figure S2. Proportion of patients with allergic reactions who were diagnosed as having anaphylaxis: food

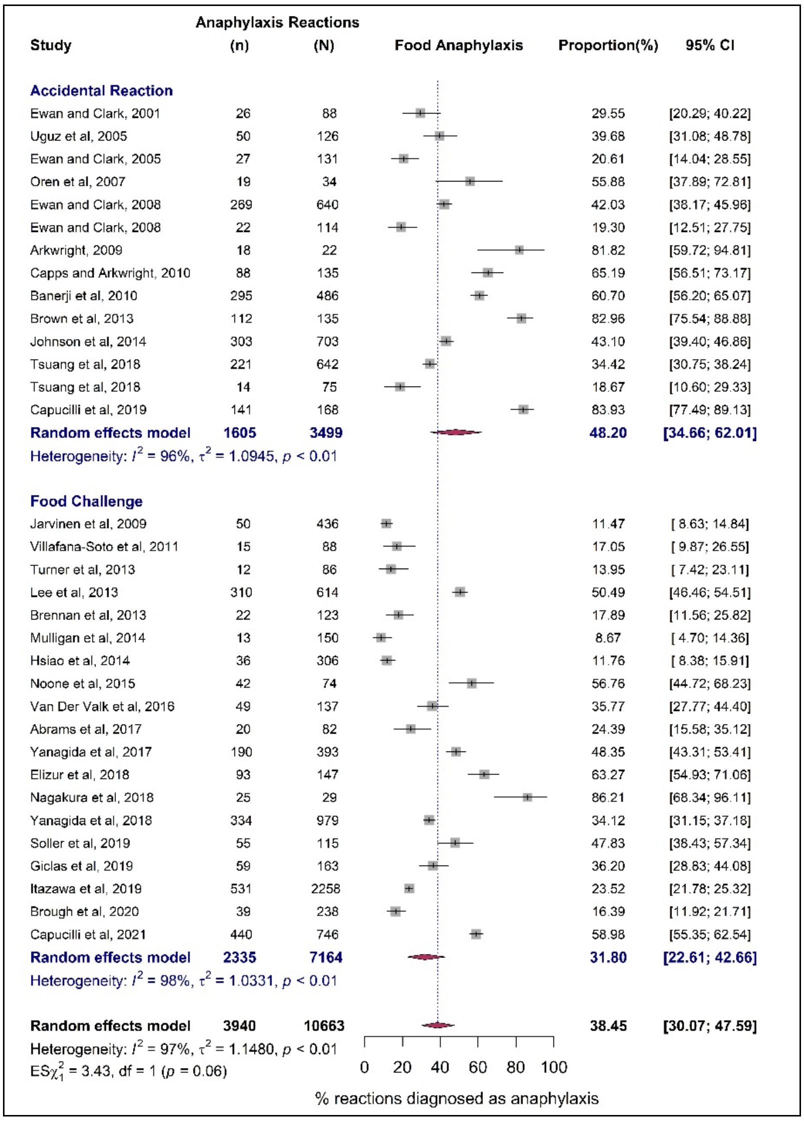

Figure S3. Proportion of patients with allergic reactions who were diagnosed as having anaphylaxis: venom

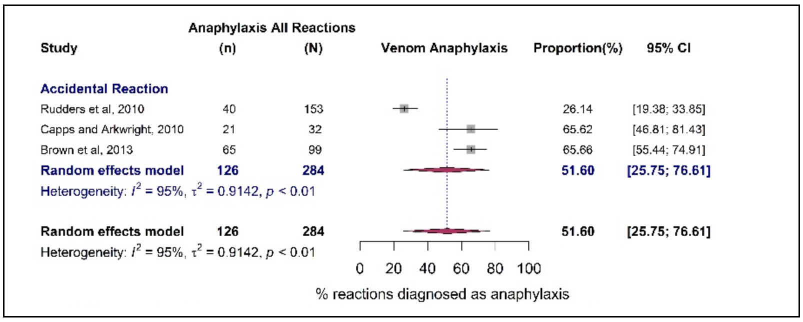

Figure S4. Proportion of anaphylaxis treated with multiple epinephrine doses: all trigger

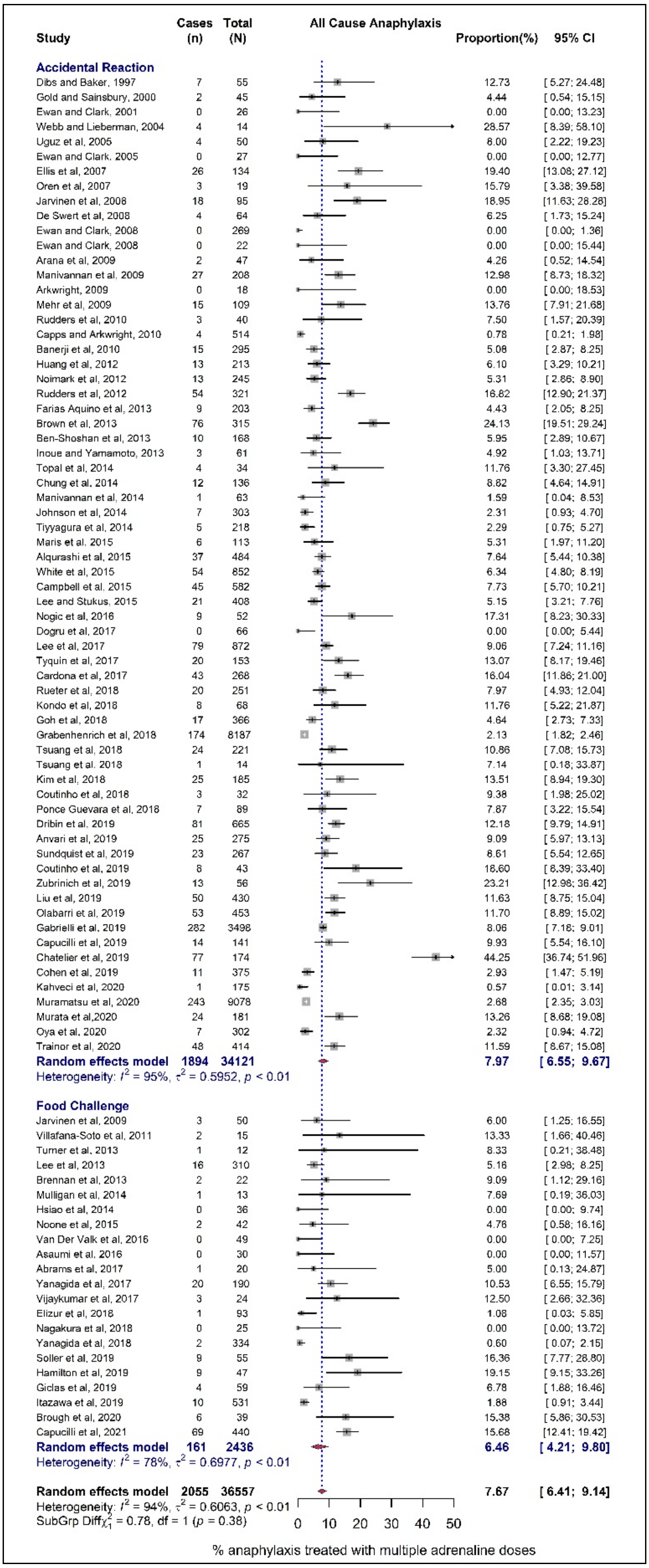

Figure S5. Proportion of anaphylaxis treated with multiple epinephrine doses: food

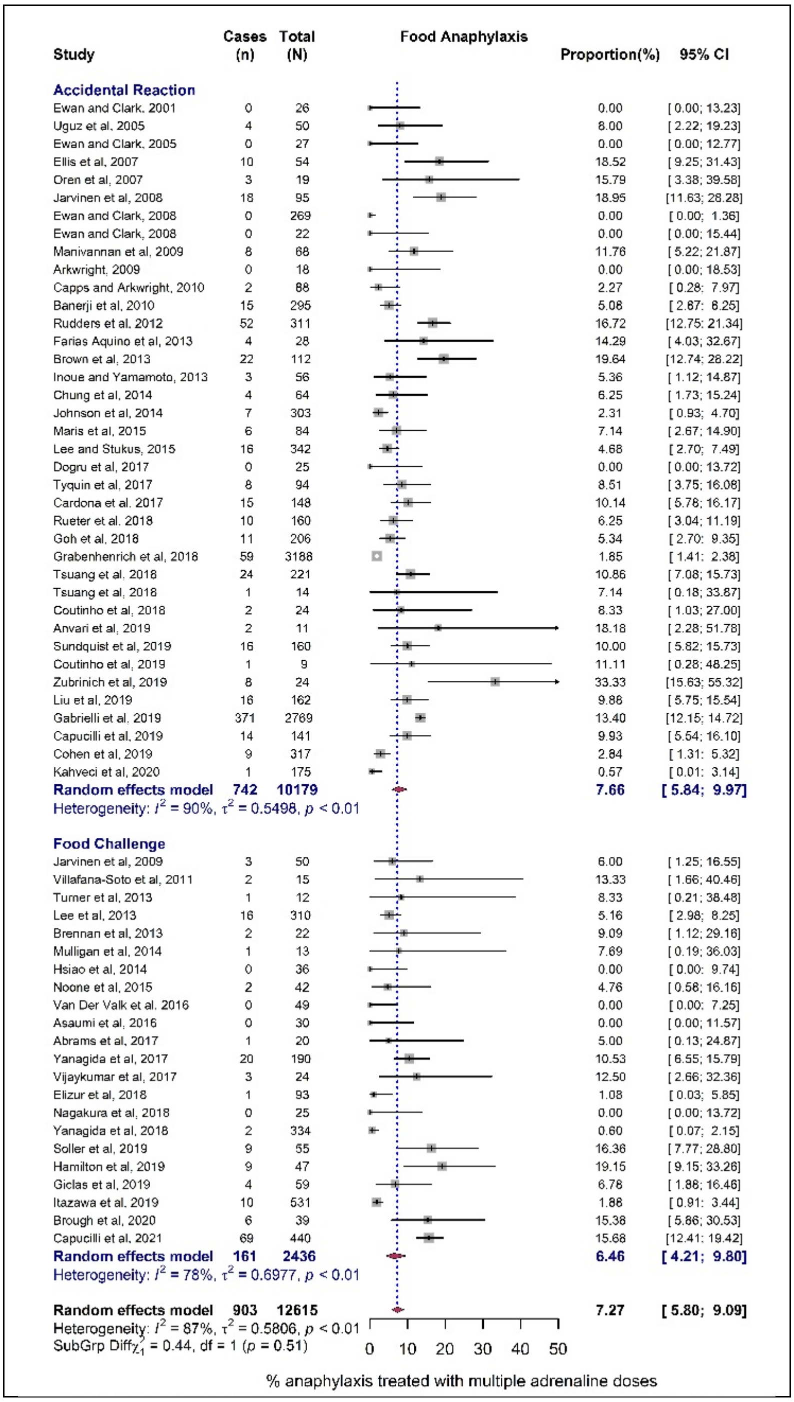

Figure S6. Proportion of anaphylaxis treated with multiple epinephrine doses: venom

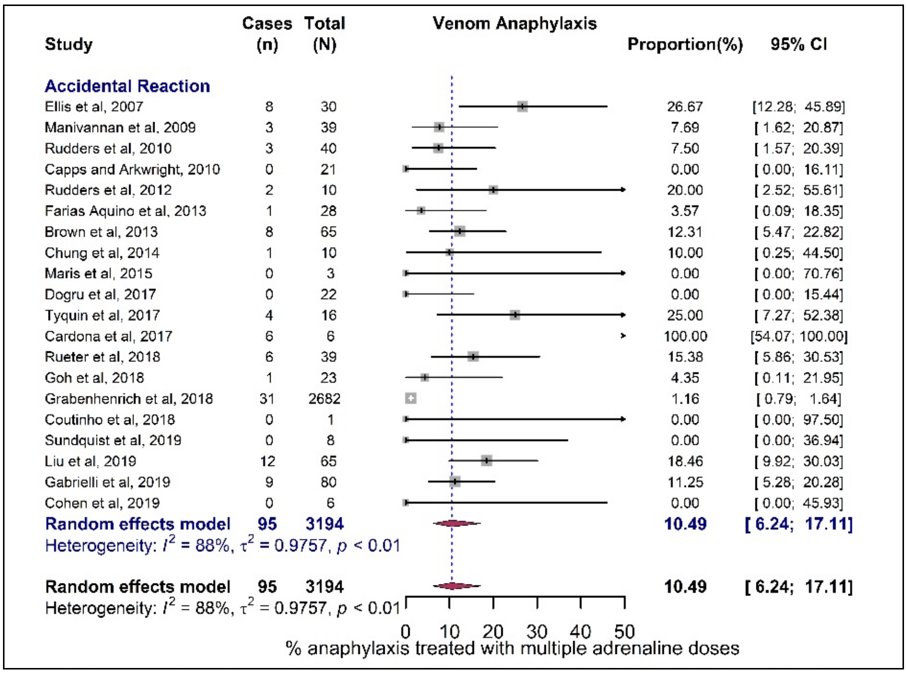

Figure S7. Cardiorespiratory reactions treated with multiple epinephrine doses: all triggers

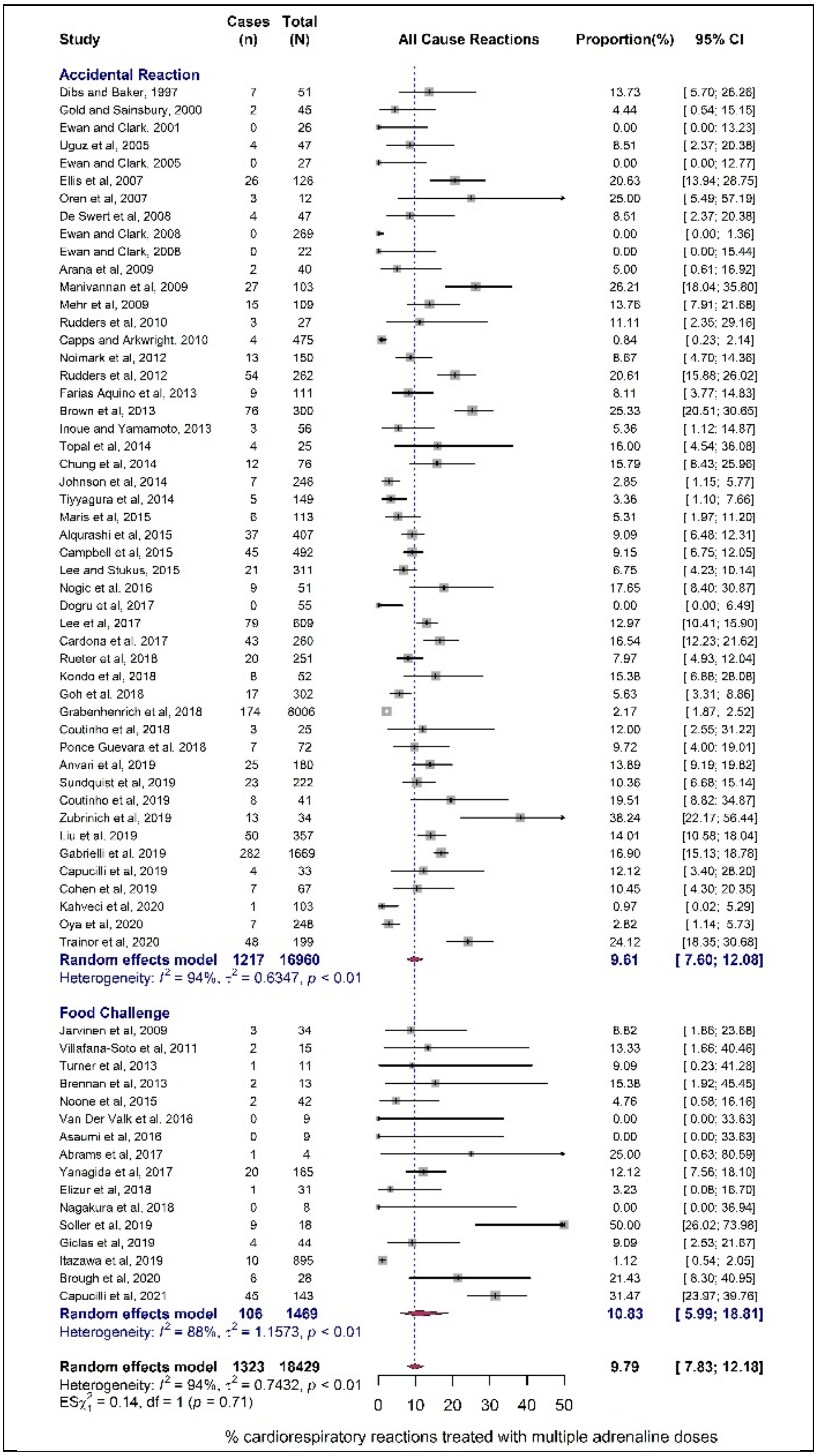

Figure S8. Cardiorespiratory reactions treated with multiple epinephrine doses: food

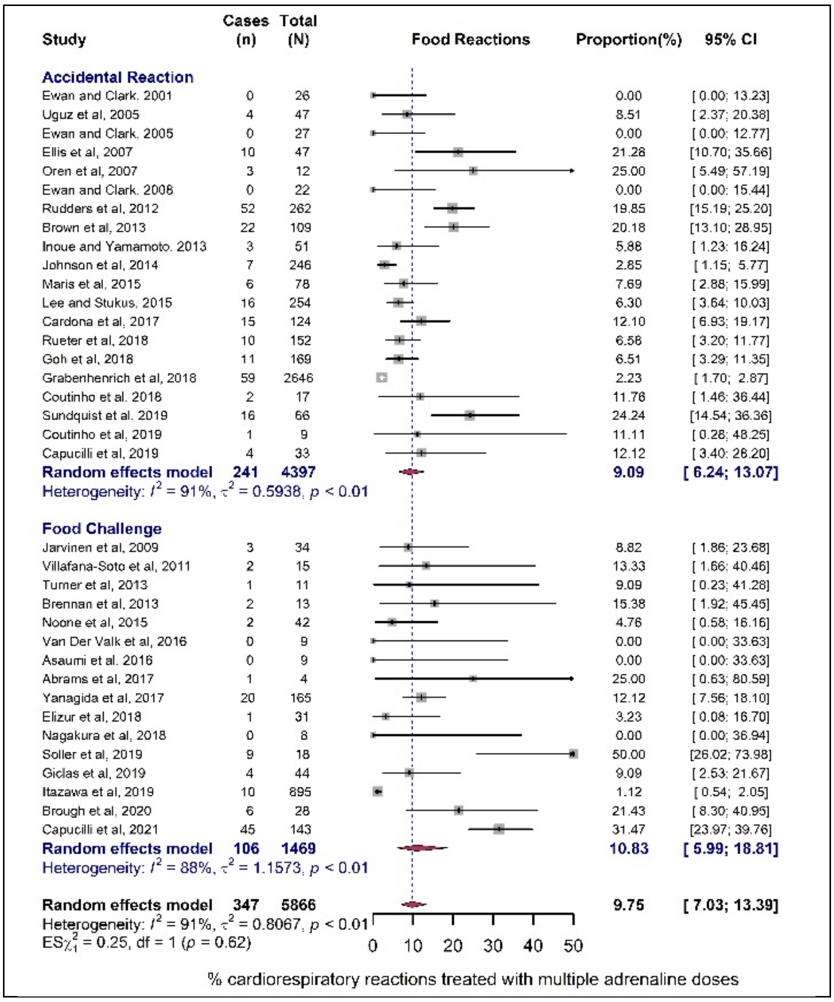

Figure S9. Cardiorespiratory reactions treated with multiple epinephrine doses: venom

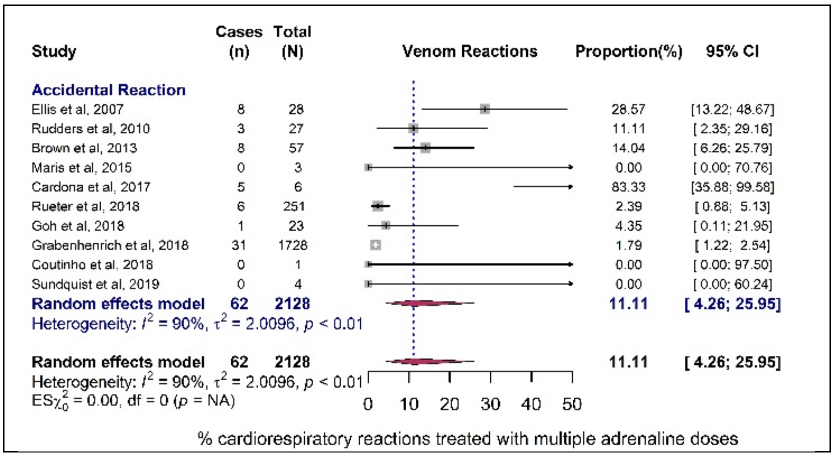

Figure S10. Epinephrine-treated reactions where multiple epinephrine doses were administered: all triggers

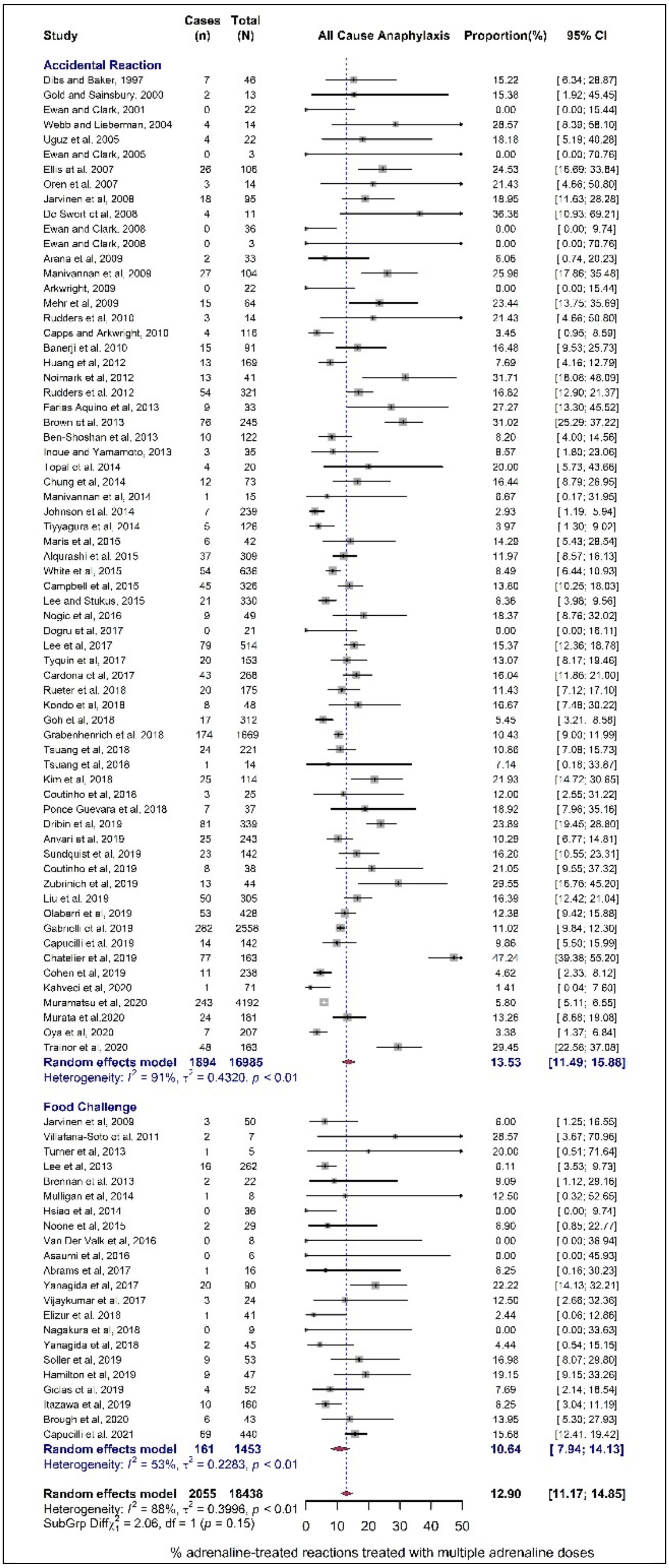

Figure S11. Epinephrine-treated reactions where multiple epinephrine doses were administered: food

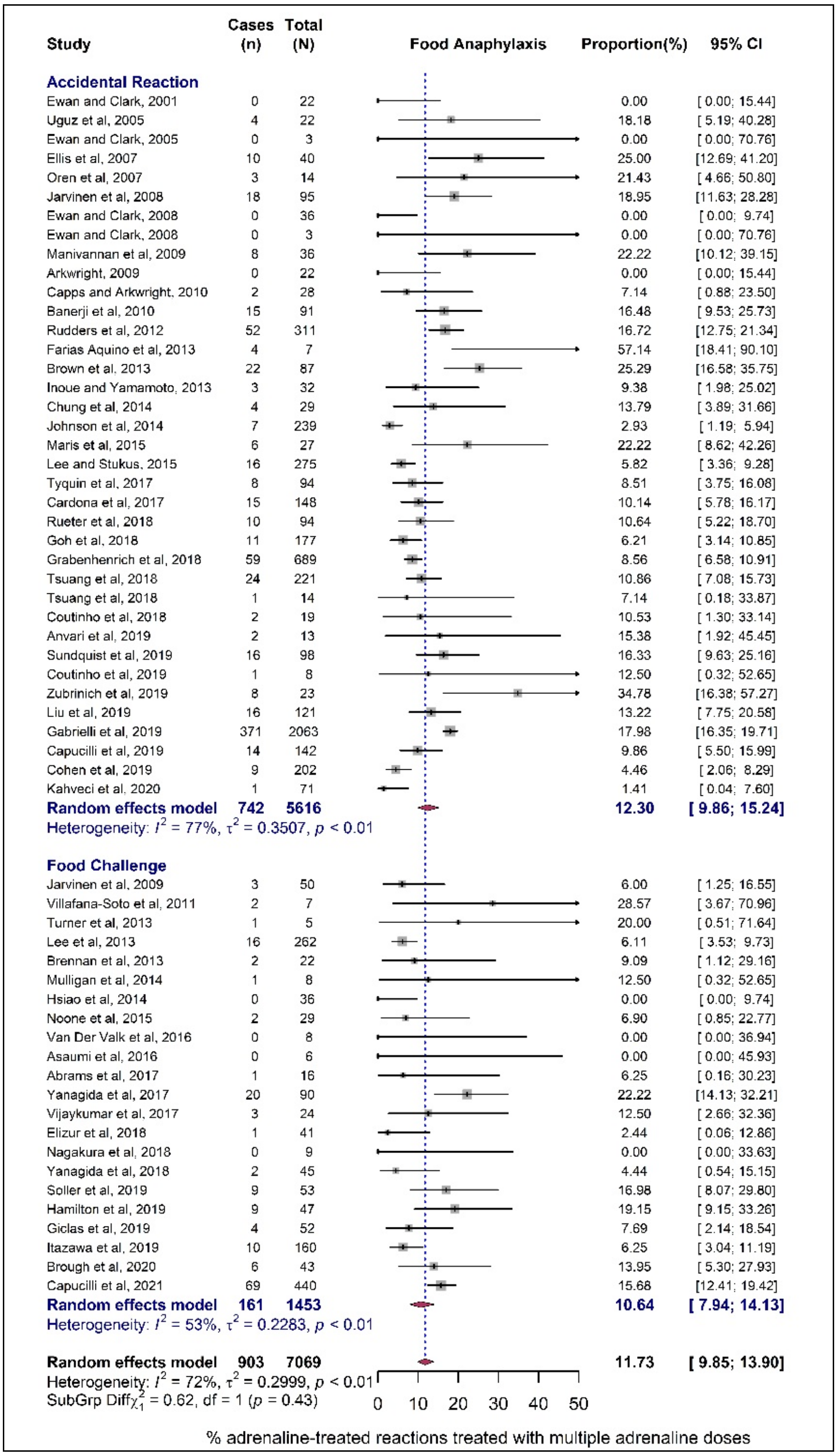

Figure S12. Epinephrine-treated reactions where multiple epinephrine doses were administered: venom

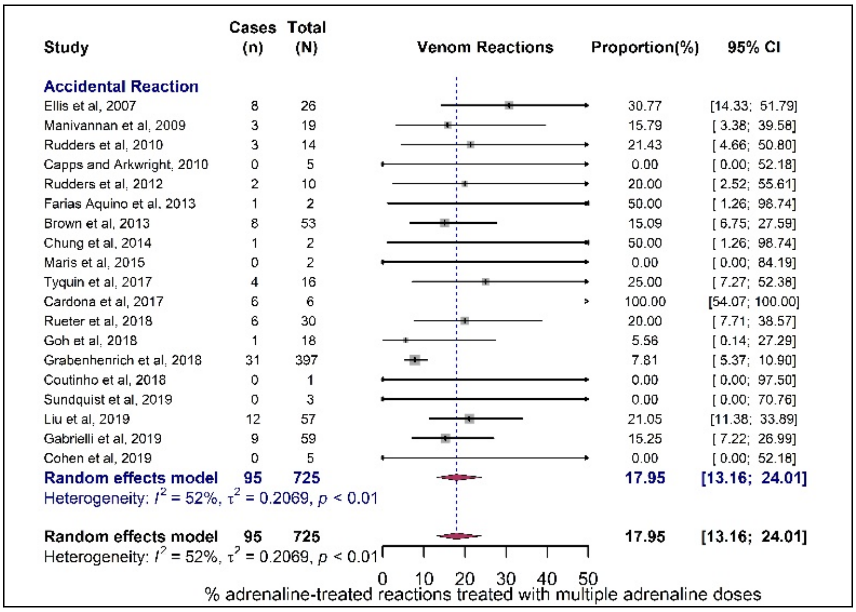

Figure S13. Anaphylaxis reactions treated with multiple epinephrine doses by a HCP: all trigger

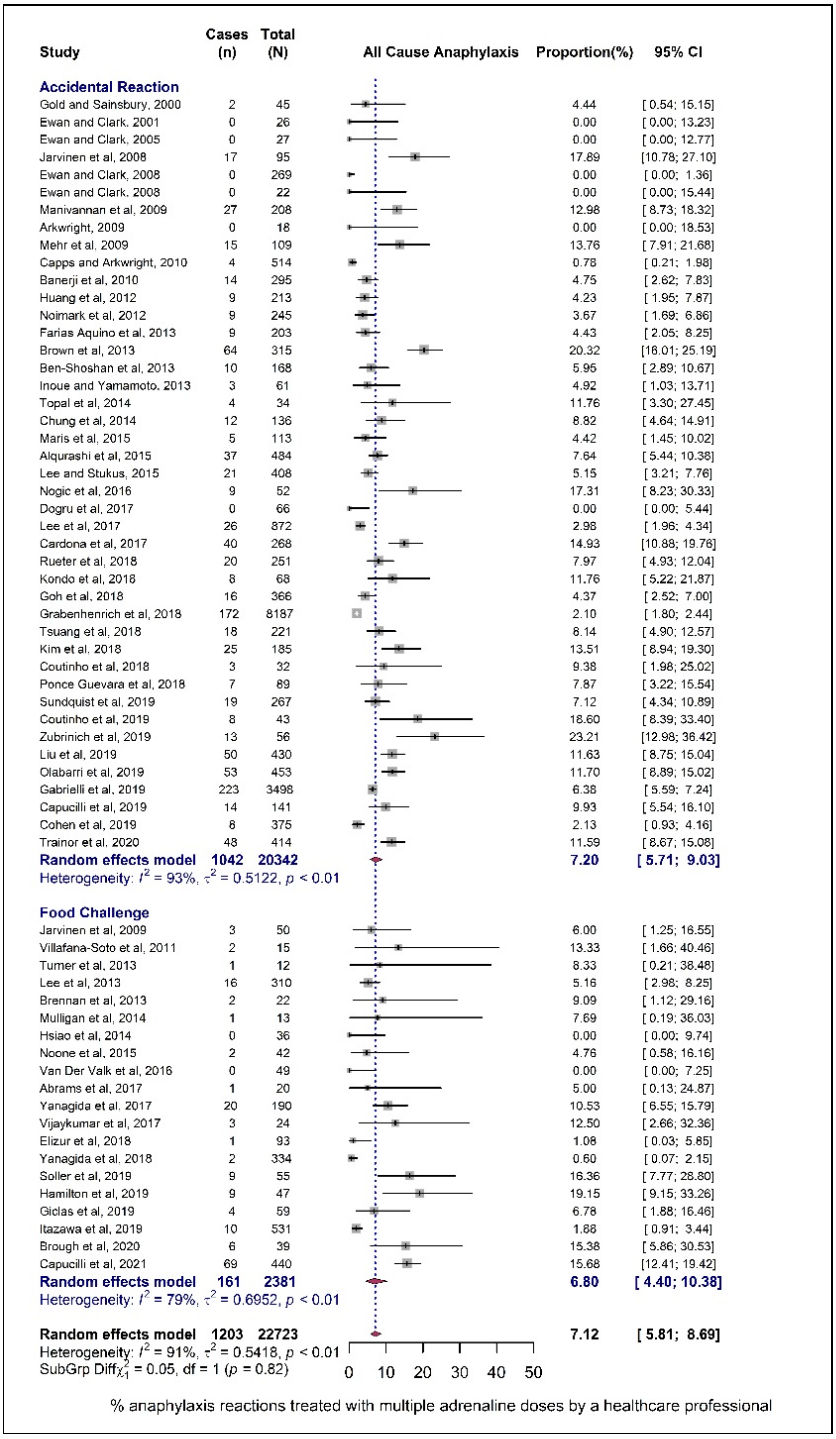

Figure S14. Anaphylaxis reactions treated with multiple epinephrine doses by a HCP: food

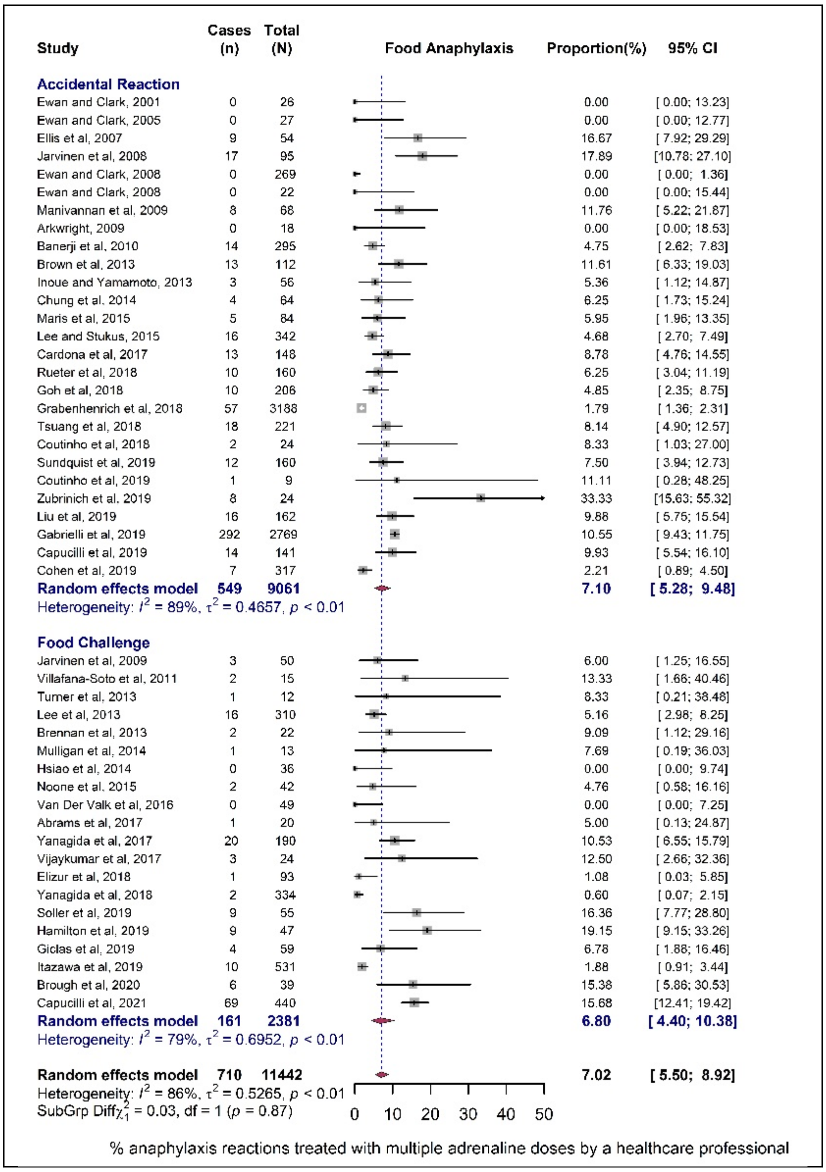

Figure S15. Anaphylaxis reactions treated with multiple epinephrine doses by a HCP: venom

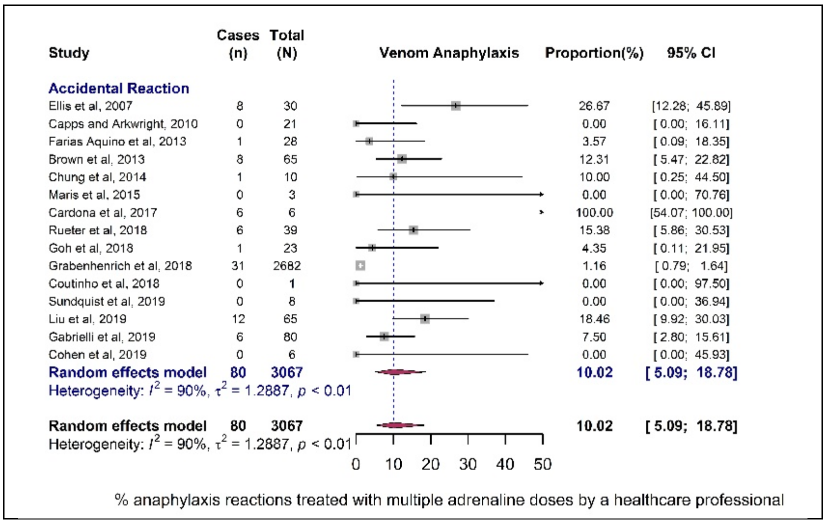

Figure S16. Epinephrine-treated reactions with multiple epinephrine doses administered by a health-care professional: all triggers

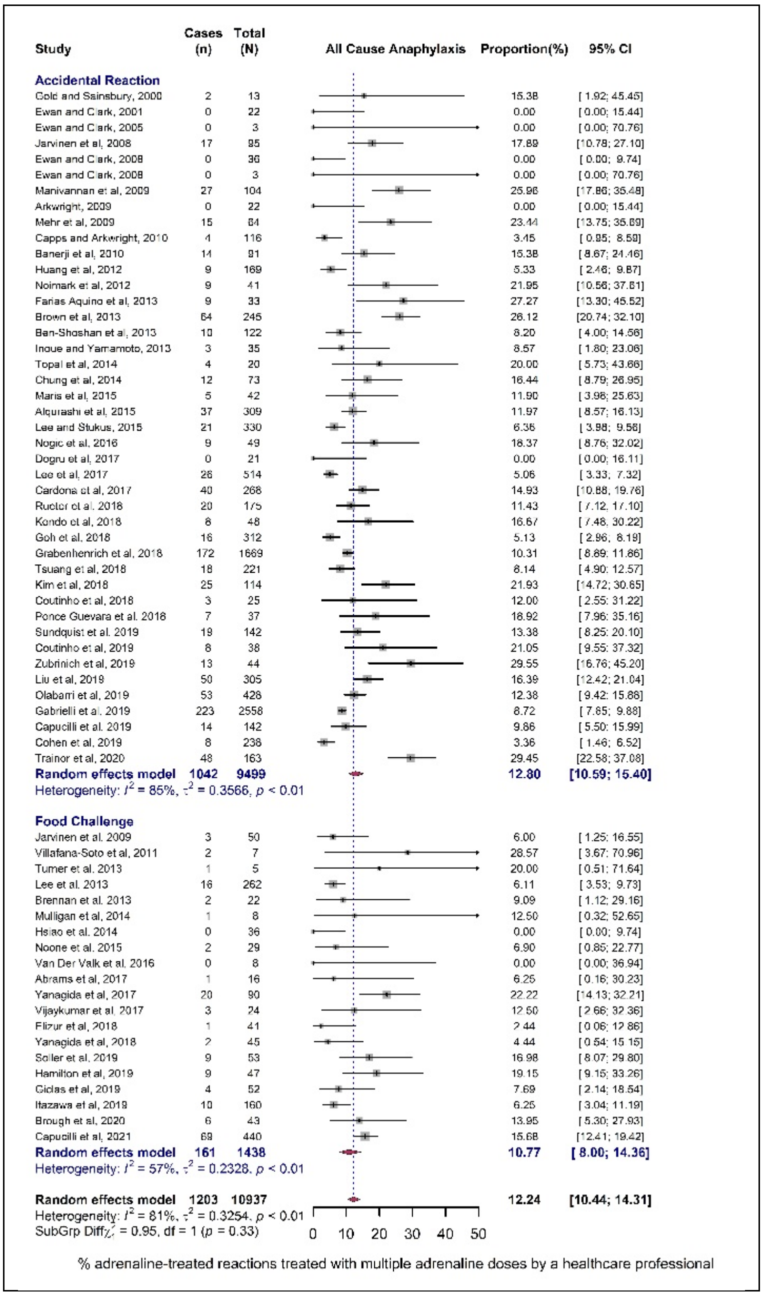

Figure S17. Epinephrine-treated reactions with subsequent epinephrine doses administered by a HCP: food

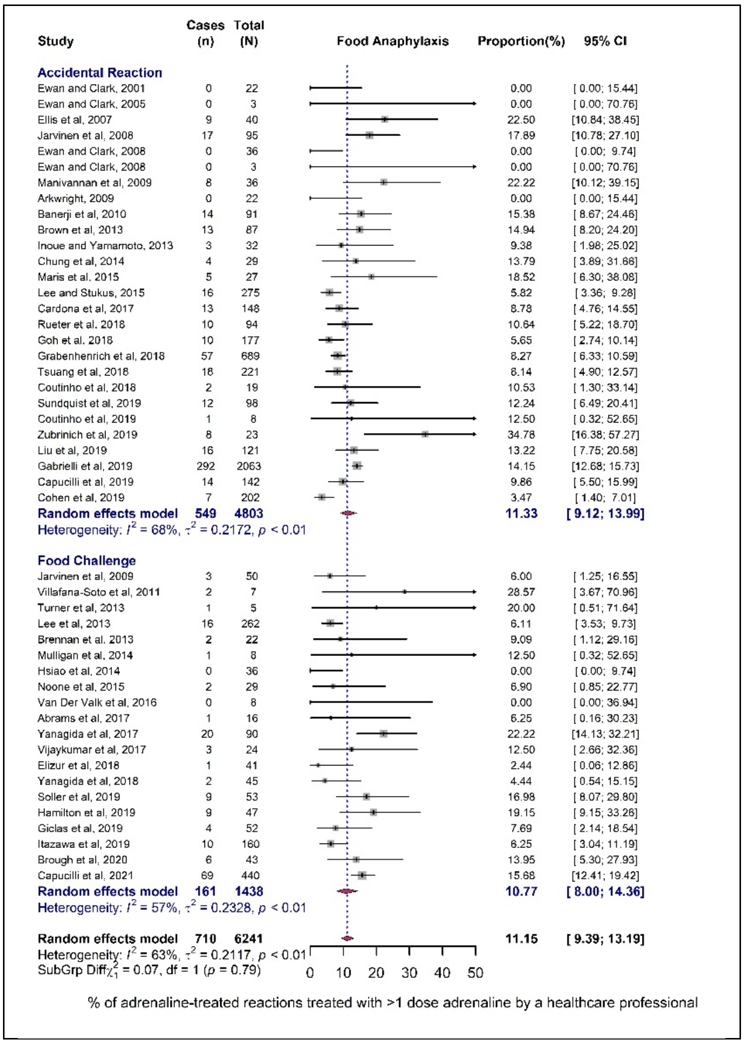

Figure S18. Epinephrine-treated reactions with subsequent epinephrine doses administered by a HCP: venom

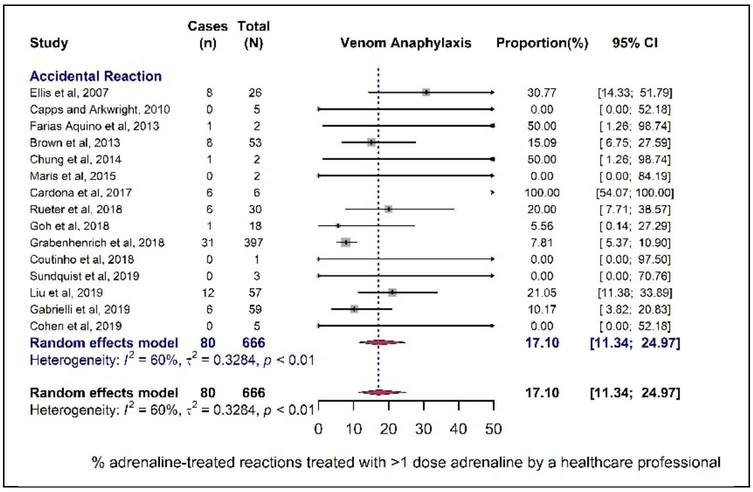

F. Funnel Plots and Egger Tests

Figure S19: Funnel Plots for all metanalyses: Assessment of small studies effect

All Cause Reactions	Food Reactions	Venom Reactions
Proportion of allergic reactions diagnosed as anaphylaxis by study authors		
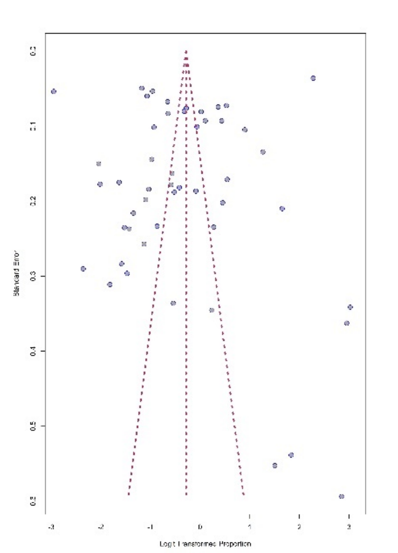	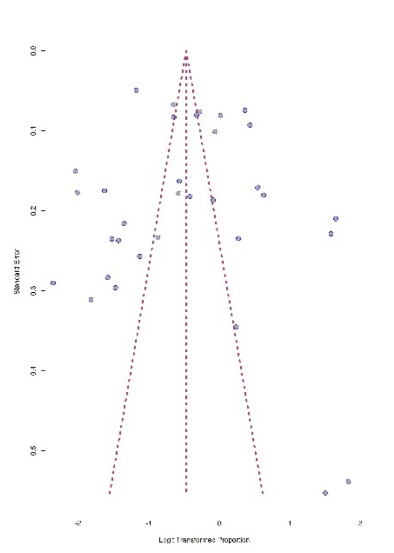	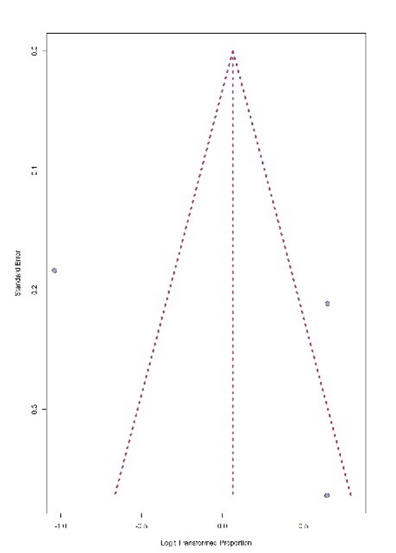
Proportion of anaphylaxis treated with multiple epinephrine doses		
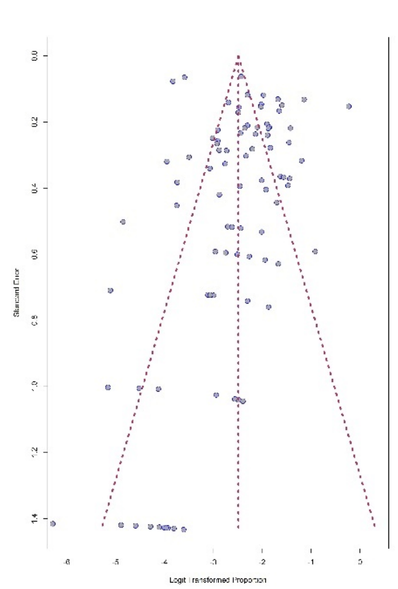	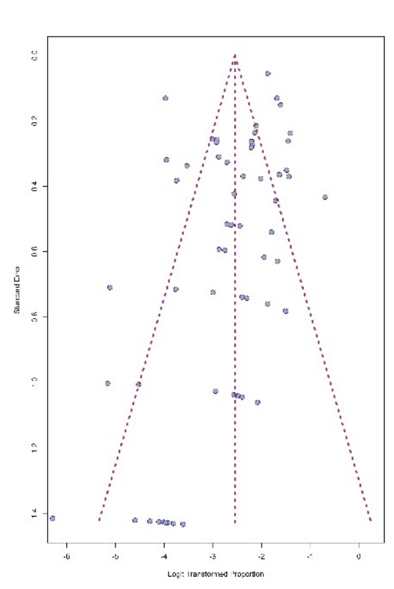	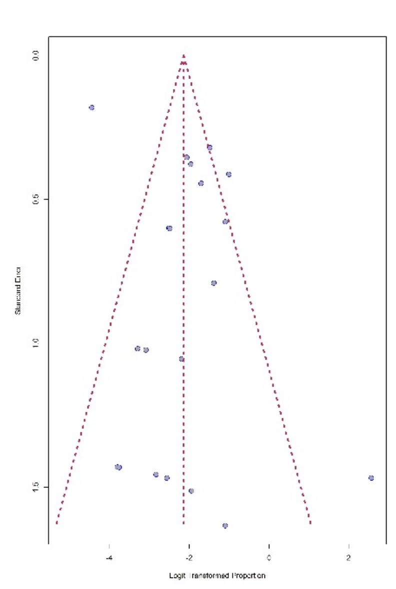
Cardiorespiratory reactions treated with multiple epinephrine doses		
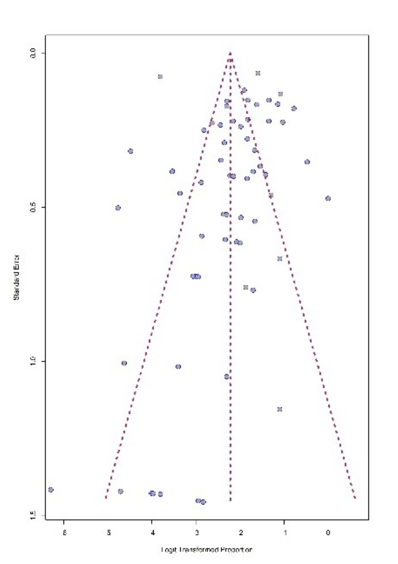	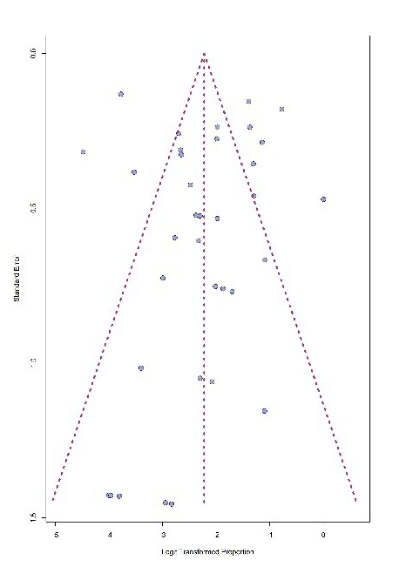	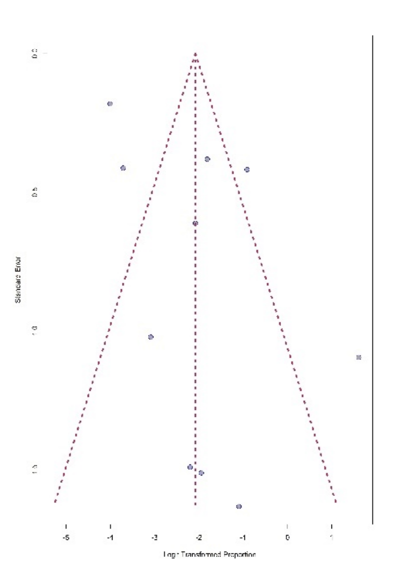

Funnel Plots for Assessment of small studies effect (publication bias) - continued

**All Cause Reactions**	**Food Reactions**	**Venom Reactions**
Epinephrine-treated reactions where multiple epinephrine doses were administered		
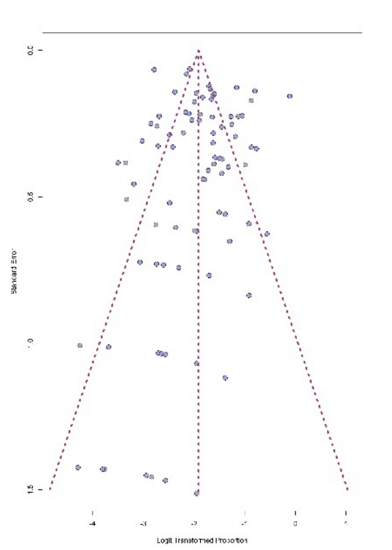	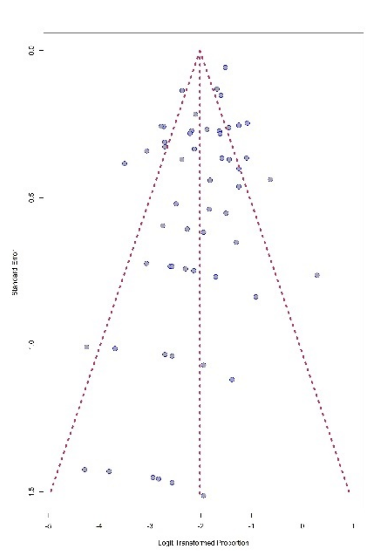	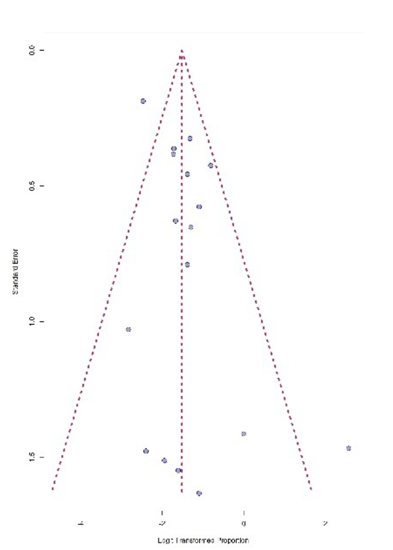
Anaphylaxis reactions treated with multiple epinephrine doses by a health-care professional		
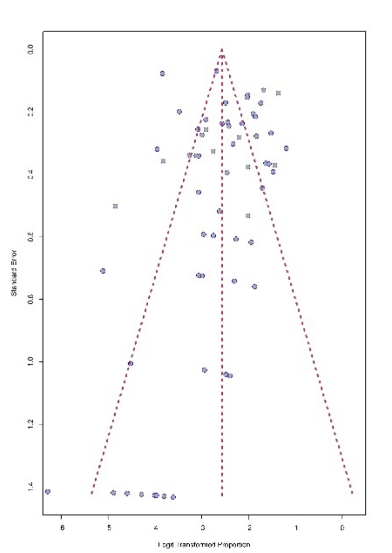	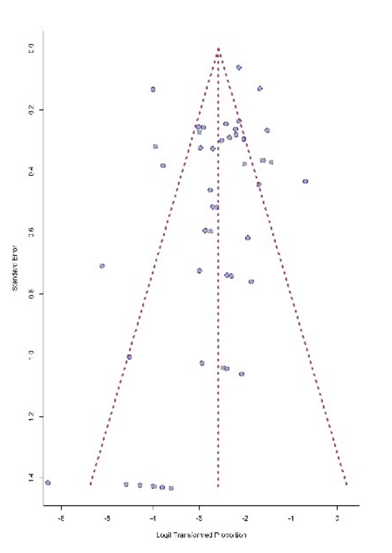	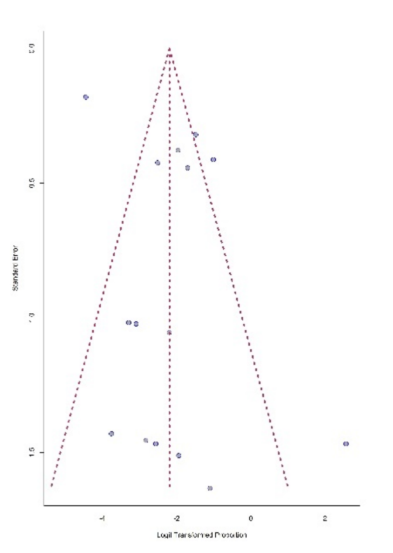
Epinephrine-treated reactions with multiple epinephrine doses administered by a health-care professional.		
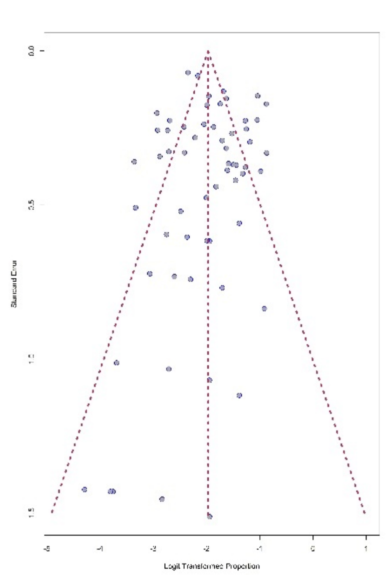	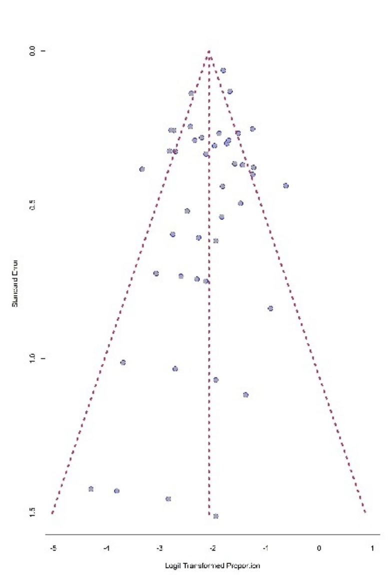	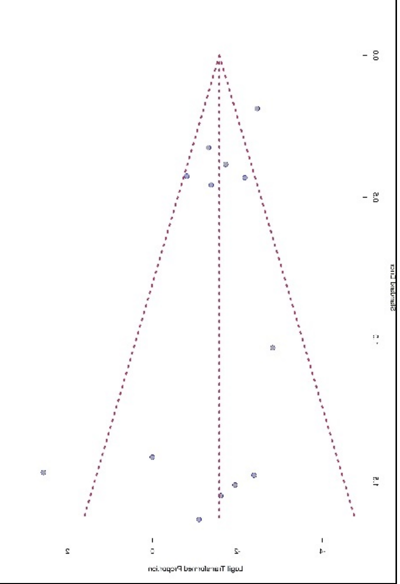

Above are funnel plots for each of the analyses listed in Table S7. Below are the results of the corresponding Egger Tests.

Table S8: Egger Test: Intercept and p value

Weighted linear regression of the treatment effect on its standard error, minimum study number = 10.

	Intercept	p value
Proportion of patients with allergic reactions who were diagnosed as having anaphylaxis		
ALL TRIGGERS	0.059	0.3956
FOOD TRIGGERS	-0.516	0.8697
VENOM TRIGGERS	-	-
Proportion of anaphylaxis treated with multiple epinephrine doses		
ALL TRIGGERS	-2.594	0.4332
FOOD TRIGGERS	-1.948	**0.0092**
VENOM TRIGGERS	-3.634	**0.0494**
Cardiorespiratory reactions treated with multiple epinephrine doses		
ALL TRIGGERS	-2.054	0.7283
FOOD TRIGGERS	-2.575	0.8961
VENOM TRIGGERS	-4.121	0.0622
Epinephrine-treated reactions where multiple epinephrine doses were administered		
ALL TRIGGERS	-1.977	0.5827
FOOD TRIGGERS	-1.588	**0.0031**
VENOM TRIGGERS	-2.275	**0.0248**
Anaphylaxis reactions treated with multiple epinephrine doses by a health-care professional		
ALL TRIGGERS	-2.631	0.6717
FOOD TRIGGERS	-2.227	0.1405
VENOM TRIGGERS	-3.795	0.0852
Epinephrine-treated reactions with multiple epinephrine doses administered by a health-care professional		
ALL TRIGGERS	-1.973	0.9855
FOOD TRIGGERS	-1.822	0.0587
VENOM TRIGGERS	-2.321	0.0741

G. Summary of Pooled Estimates for Sensitivity Analyses

The pooled estimates of each of the metanalyses conducted for the listed sensitivity analyses are summarised below. Data are presented by trigger and anaphylaxis definition as % (95% CI).

A. Prospective Studies only

Table S9. Pooled estimates for prospective studies only.

%reactions treated with >1 dose epinephrine % (95% CI)	Trigger		
	**ALL**	**FOOD**	**VENOM**
	**Any Setting N=14,659**	**Any Setting N=7,596**	**Any Setting N=2,925**
A) Study-defined anaphylaxis	5.5% (3.3 to 9.2%)	5.5% (3.1 to 9.6%)	9.9% (3.6 to 24.7%)
B) "Cardiorespiratory" anaphylaxis	7.8% (4.1 to 14.2%)	7.3% (3.4 to 15.0%)	9.7% (2.3 to 32.4%)
C) Reaction treated with at least one dose of epinephrine	11.6% (8.3 to 15.9%)	12.9% (9.5 to 17.4%)	15.9% (9.9 to 24.6%)
D) Reaction where further epinephrine administered by a healthcare professional	5.1% (2.9 to 9.0%)	5.1% (2.9 to 9.0%)	9.3% (3.3 to 23.3%)
E) Epinephrine-treated reaction where with further epinephrine administered by a healthcare professional	11.7% (8.8 to 15.5%)	11.9% (9.3 to 15.2%)	14.9% (9.0 to 23.7%)

B. LOW Risk of Bias Studies only

Table S10. Pooled estimates for low risk of bias studies only.

%reactions treated with >1 dose epinephrine % (95% CI)	Trigger		
	**ALL**	**FOOD**	**VENOM**
	**Any Setting N=22,294**	**Any Setting N=10,159**	**Any Setting N=3,040**
A) Study-defined anaphylaxis	7.4% (5.9 to 9.3%)	6.6% (4.8 to 9.0%)	9.6% (5.2 to 17.2%)
B) "Cardiorespiratory" anaphylaxis	10.3% (7.7 to 13.6%)	9.5% (5.7 to 15.3%)	6.9% (2.3 to 19.0%)
C) Reaction treated with at least one dose of epinephrine	12.9% (10.6 to 15.7%)	11.0% (8.6 to 14.0%)	17.5% (12.2 to 24.5%)
D) Reaction where further epinephrine administered by a healthcare professional	6.8% (5.3 to 8.7%)	6.6% (4.7 to 9.2%)	9.1% (4.0 to 19.5%)
E) Epinephrine-treated reaction where with further epinephrine administered by a healthcare professional	11.8% (9.7 to 14.4%)	11.2% (8.8 to 14.1%)	16.0% (9.9 to 24.9%)

C. Studies Published after 2006 only

Table S11. Pooled estimates for studies published after 2006 only.

%reactions treated with >1 dose epinephrine % (95% CI)	Trigger		
	**ALL**	**FOOD**	**VENOM**
	**Any Setting N=36,340**	**Any Setting N=12,512**	**Any Setting N=3194**
A) Study-defined anaphylaxis	7.6% (6.3 to 9.1%)	7.4% (5.8 to 9.3%)	10.5% (6.2 to 17.1%)
B) "Cardiorespiratory" anaphylaxis	10.0% (7.9 to 12.6%)	10.2% (7.3 to 14.1%)	11.1% (4.3 to 26.0%)
C) Reaction treated with at least one dose of epinephrine	12.7% (11.0 to 14.8%)	11.7% (9.9 to 13.9%)	18.0% (13.2 to 24.0%)
D) Reaction where further epinephrine administered by a healthcare professional	7.2% (5.9 to 8.9%)	7.2% (5.6 to 9.1%)	10.0% (5.1 to 18.8%)
E) Epinephrine-treated reaction where with further epinephrine administered by a healthcare professional	12.3% (10.4 to 14.4%)	11.2% (9.4 to 13.3%)	17.1% (11.3 to 25.0%)

D. Studies published as Full-Texts in peer-reviewed journals only

Table S12. Pooled estimates for Full-Text references only.

%reactions treated with >1 dose epinephrine % (95% CI)	Trigger		
	**ALL**	**FOOD**	**VENOM**
	**Any Setting N=34,790**	**Any Setting N=11,851**	**Any Setting N=3147**
A) Study-defined anaphylaxis	7.3% (6.1 to 8.9%)	6.8% (5.2 to 8.8%)	10.6% (6.2 to 17.5%)
B) "Cardiorespiratory" anaphylaxis	9.9% (7.8 to 12.6%)	9.8% (6.7 to 14.0%)	11.1% (4.0 to 27.5%)
C) Reaction treated with at least one dose of epinephrine	12.7% (10.9 to 14.7%)	11.5% (9.5 to 13.9%)	17.2% (12.3 to 23.4%)
D) Reaction where further epinephrine administered by a healthcare professional	6.9% (5.5 to 8.7%)	6.7% (5.2 to 8.9%)	10.7% (5.1 to 21.0%)
E) Epinephrine-treated reaction where with further epinephrine administered by a healthcare professional	12.2% (10.2 to 14.5%)	11.1% (9.2 to 13.4%)	16.5% (10.8 to 24.5%)

H. Record Flow: Excluded Records at Full-Text screening

The references reviewed at during the full-text screening, along with outcome and reason are listed below.

Table S13: References excluded at full-text screening with reason for exclusion provided

AUTHOR	TITLE	YR	JOURNAL	SCREENING OUTCOME	REASON for OUTCOME
Alvarez-Perea, A. A., B.; Morales, C.; Zambrano, G.; Rodriguez, A.; Guzman, M.; Zubeldia, J. M.; Baeza, M. L.	Anaphylaxis in the Pediatric Emergency Department: Analysis of 133 Cases After an Allergy Workup	2017	Journal of Allergy and Clinical Immunology: In Practice	Excluded at Full-Text Screen	No 2nd dose data
Yanagida, N. S., S.; Asaumi, T.; Ogura, K.; Borres, M. P.; Ebisawa, M.	Safety and feasibility of heated egg yolk challenge for children with egg allergies	2017	Pediatric Allergy and Immunology	Excluded at Full-Text Screen	No 2nd dose data
Gawlik, R. M., M.; Kolodziej, I.; Dzienniak, A.; Bozek, A.	First-line treatment of hymenoptera venom anaphylaxis: a 23-year real-life experience	2017	Annals of Allergy, Asthma and Immunology	Excluded at Full-Text Screen	No 2nd dose data
Jeong, K. L., S. Y.; Ahn, K.; Kim, J.; Lee, H. R.; Suh, D. I.; Pyun, B. Y.; Min, T. K.; Kwon, J. W.; Kim, K. E.; Kim, K. W.; Sohn, M. H.; Kim, Y. H.; Song, T. W.; Kwon, J. H.; Jeon, Y. H.; Kim, H. Y.; Kim, J. H.; Ahn, Y. M.; Lee, S.	A multicenter study on anaphylaxis caused by peanut, tree nuts, and seeds in children and adolescents	2017	Allergy: European Journal of Allergy and Clinical Immunology	Excluded at Full-Text Screen	No 2nd dose data
Lee, A. Y. M. E., P.; Clarke, A. E.; La Vieille, S.; Eisman, H.; Chan, E. S.; Mill, C.; Joseph, L.; Ben-Shoshan, M.	Anaphylaxis across two canadian pediatric centers: Evaluating management disparities	2017	Journal of Asthma and Allergy	Excluded at Full-Text Screen	No 2nd dose data
Lee, S. H., E. P.; Lohse, C.; Gilani, W.; Chamberlain, A. M.; Campbell, R. L.	Trends, characteristics, and incidence of anaphylaxis in 2001-2010: A population-based study	2017	Journal of Allergy and Clinical Immunology	Excluded at Full-Text Screen	No 2nd dose data
Lee, S. P., A.; Lohse, C. M.; Hess, E. P.; Campbell, R. L.	Derivation of a clinical decision rule to predict biphasic reactions in emergency department anaphylaxis patients	2017	Academic Emergency Medicine	Excluded at Full-Text Screen	No 2nd dose data
Vetander, M. P., J. L. P.; Lilja, G.; Kull, I.; Hedlin, G.; van Hage, M.; Ostblom, E.; Bergstrom, A.; Wickman, M.	Anaphylaxis to foods in a population of adolescents: incidence, characteristics and associated risks	2016	Clinical and Experimental Allergy	Excluded at Full-Text Screen	No 2nd dose data
Vasar, M. L., A.; Julge, K.; Kivivare, M.; Voor, T.	Anaphylaxis cases in children's clinic of tartu university hospital during the years 2005-2014	2016	Eesti Arst	Excluded at Full-Text Screen	No 2nd dose data
Grzyb, M. J. C., A.; Kimchi, N.; Lachaine, C.; La Vieille, S.; Joseph, L.; Mill, C.; Ben-Shoshan, M.	Anaphylaxis cases treated by out-of-hospital EMS in Western Quebec	2016	Journal of Allergy and Clinical Immunology	Excluded at Full-Text Screen	No 2nd dose data
Blaziene, A. B., N.; Paltarackiene, V.; DuBuske, L. M.	Analysis of anaphylaxis trigger factors and treatment during a five year period in a vilnius university hospital	2016	Journal of Allergy and Clinical Immunology	Excluded at Full-Text Screen	No 2nd dose data
Worm, M. D., S.; Francuzik, W.	Data from the anaphylaxis registry of the German-speaking countries	2015	Revue Francaise d'Allergologie	Excluded at Full-Text Screen	Multiple publications of dataset

Katz, Y. N.-W., A.; Spergel, J. M.	Prevalence of biphasic response in anaphylaxis due to purposeful administration of allergenic food	2015	Annals of Allergy, Asthma and Immunology	Excluded at Full-Text Screen	No 2nd dose data
Dhami, S. R., L.; Austin, M.; Sheikh, A.	Anaphylaxis in the community: a questionnaire survey of members of the UK Anaphylaxis Campaign	2015	JRSM Open	Excluded at Full-Text Screen	No 2nd dose data
Robb, A. L. T., T. W.	Pre-hospital and emergency room management of pediatric anaphylaxis	2015	Canadian Journal of Emergency Medicine	Excluded at Full-Text Screen	No 2nd dose data
Fitzgerald, A. S., S.; Koonamave, R.	Audit of failed inpatient oral food challenges in the peadiatric population	2015	Internal Medicine Journal	Excluded at Full-Text Screen	No 2nd dose data
Patel, M. Z., C.; O'Hehir, R.; Hew, M.	Emergency management of anaphylaxis at a tertiary centre	2015	Internal Medicine Journal	Excluded at Full-Text Screen	No 2nd dose data
Dang, A. T. C., P.; Perez Ramirez, L.; Morris, D.; Goodman, M.; Assa'ad, A. H.	Epinephrine ordering and utilization for inoffice oral food challenges: Standardization of practice	2015	Annals of Allergy, Asthma and Immunology	Excluded at Full-Text Screen	No 2nd dose data
Sandilos, C. P., M.; Gkavogiannakis, N.; Skarlatou, G.; Aggelides, X.; Chliva, C.; Makris, M.	Anaphylaxis in the emergency department: Data of a Greek tertiary hospital	2015	Allergy: European Journal of Allergy and Clinical Immunology	Excluded at Full-Text Screen	No 2nd dose data
McLaughlin, C. C., J.; Aralihond, A.	An audit of the management of anaphylaxis in children in a district general hospital	2015	Archives of Disease in Childhood	Excluded at Full-Text Screen	No 2nd dose data
Mitthamsiri, W. H., S.; Ruxrungtham, K.	Food-induced anaphylaxis in thailand: A 10 years data from a tertiary care center in bangkok	2015	Journal of Allergy and Clinical Immunology	Excluded at Full-Text Screen	No 2nd dose data
Aranez, V. T. L., M. G.; Relan, M.; Qiao, H.; Wrotniak, B.; Lehman, H. K.	An evaluation of the treatment of anaphylaxis in a pediatric emergency room setting	2015	Journal of Allergy and Clinical Immunology	Excluded at Full-Text Screen	No 2nd dose data
Hemler, J. A. S., H. P.	Management of children with anaphylaxis in an urban emergency department	2015	Journal of Allergy and Clinical Immunology	Excluded at Full-Text Screen	No 2nd dose data
O'Connor, M. J. E., M. K.	Anaphylaxis in a rural emergency department: The dartmouth-hitchcock experience	2015	Journal of Allergy and Clinical Immunology	Excluded at Full-Text Screen	No 2nd dose data
Torabi, B. M., J.; Clarke, A.; La Vieille, S.; Alizadehfar, R.; Joseph, L.; Ben-Shoshan, M.	Anaphylaxis cases presenting to the emergency center over a two year period in Montreal, Canada	2015	Journal of Allergy and Clinical Immunology	Excluded at Full-Text Screen	No 2nd dose data
Alangari, A. A.	Characteristics of patients presenting to the emergency department with anaphylaxis in Riyadh, Saudi Arabia	2014	Journal of Taibah University Medical Sciences	Excluded at Full-Text Screen	No 2nd dose data
Lee, S. B., M. F.; Hess, E. P.; Campbell, R. L.	Predictors of Biphasic Reactions in the Emergency Department for Patients With Anaphylaxis	2014	Journal of Allergy and Clinical Immunology: In Practice	Excluded at Full-Text Screen	No 2nd dose data
Clark, S. W., W.; Rudders, S. A.; Camargo, C. A.	Risk factors for severe anaphylaxis in patients receiving anaphylaxis treatment in US emergency departments and hospitals	2014	Journal of Allergy and Clinical Immunology	Excluded at Full-Text Screen	No 2nd dose data
Charatsi, A. M. M., S.; Aversano, G.; Casimir, G.	P32-Food anaphylaxis experience in children in Brussels	2014	Clinical and Translational Allergy	Excluded at Full-Text Screen	No 2nd dose data
Gupta, R. D.-C., L.; Rivkina, V.	Emergency epinephrine use for food allergy reactions in Chicago Public Schools	2014	Annals of Allergy, Asthma and Immunology	Excluded at Full-Text Screen	No 2nd dose data
Shima, D. I., Y.; Yamamoto, Y.; Nagayasu, S.; Fujimoto, Y.	A database study to investigate the incidence of anaphylaxis and the prescription rate of self-injection epinephrine in Japan	2014	Value in Health	Excluded at Full-Text Screen	No 2nd dose data

Larionova, A. V., T.; Vasar, M.; Julge, K.; Kivivare, M.	Pediatric anaphylaxis cases between 2009-2013 in Estonia: A single-centre experience	2014	Allergy: European Journal of Allergy and Clinical Immunology	Excluded at Full-Text Screen	No 2nd dose data
Ameiro, B. N., B.; Zambrano, G.; Morales, C.; Guzman, M.; Baeza, M. L.; Alvarez-Perea, A.	Characteristics of anaphylaxis in a pediatric emergency unit	2014	Journal of Allergy and Clinical Immunology	Excluded at Full-Text Screen	No 2nd dose data
Kimchi, N. C., A. E.; Moisan, J.; Lachaine, C.; Vieille, S. L.; Asai, Y.; Joseph, L.; Mill, C.; Ben-Shoshan, M.	Anaphylaxis cases presenting to primary care paramedics in Quebec	2014	Journal of Allergy and Clinical Immunology	Excluded at Full-Text Screen	No 2nd dose data
Topal, E. B., A.; Yilmaz, O.; Karagol, I. H. E.; Arga, M.; Demirsoy, M. S.; Turktas, I.	Epidemiological and clinical features of anaphylaxis: Single center experience with 109 children	2013	Pediatric, Allergy, Immunology, and Pulmonology	Excluded at Full-Text Screen	No 2nd dose data
Liew, W. K. C., W. C.; Goh, A. E.; Lim, H. H.; Chay, O. M.; Chang, S.; Tan, J. H.; Shih, E.; Kidon, M.	Paediatric anaphylaxis in a Singaporean children cohort: Changing food allergy triggers over time	2013	Asia Pacific Allergy	Excluded at Full-Text Screen	No 2nd dose data
Sala-Cunill, A. C., V.; Labrador-Horrillo, M.; Luengo, O.; Esteso, O.; Garriga, T.; Vicario, M.; Guilarte, M.	Usefulness and limitations of sequential serum tryptase for the diagnosis of anaphylaxis in 102 patients	2013	International Archives of Allergy and Immunology	Excluded at Full-Text Screen	No 2nd dose data
Yavuz, S. T. S., U. M.; Buyuktiryaki, B.; Soyer, O. U.; Sackesen, C.; Sekerel, B. E.; Tuncer, A.	Clinical features of children with venom allergy and risk factors for severe systemic reactions	2013	International Archives of Allergy and Immunology	Excluded at Full-Text Screen	No 2nd dose data
Gushken, A. F. W., L. A.; Beck, C. L.; Castro, A. P. B. M.; Yonamine, G. H.; Pastorino, A. C.; Jacob, C. M. A.	Anaphylactic reactions during oral food challenge test in pediatric patients	2013	Clinical and Translational Allergy	Excluded at Full-Text Screen	No 2nd dose data
Moneret-Vautrin, A.	Emergency treatment of food anaphylaxis: A report of 152 cases registered by the Allergy Vigilance Network	2013	Clinical and Translational Allergy	Excluded at Full-Text Screen	No 2nd dose data
Haltinner, K. K., A.	Anaphylaxis in children-data from Zurich	2013	Swiss Medical Weekly	Excluded at Full-Text Screen	No 2nd dose data
Mitchell, R. C. D., N. M.; Akindolie, O.; Braithwaite, N.	The management and follow-up of patients presenting with anaphylaxis to the paediatric emergency department	2013	Clinical and Experimental Allergy	Excluded at Full-Text Screen	No 2nd dose data
Krishnamoorthy, S. K., S. G.; Rao, R.	Anaphylaxis in children: 2 years audit of practice in a tertiary children's hospital in Asia-Pacific Region	2013	Clinical and Experimental Allergy	Excluded at Full-Text Screen	No 2nd dose data
Zolkipli, Z. E.-L., M.; Grainger-Allen, E.; Roberts, G.	How many adrenaline autoinjectors should be prescribed to patients at risk of anaphylaxis - A systematic review	2013	Clinical and Experimental Allergy	Excluded at Full-Text Screen	No 2nd dose data
Farah, N. W., A.; Joyce, M.; Rajakulasingam, K.	An audit analysis of anaphylaxis presenting at Homerton University Hospital (HUH)	2013	Clinical and Experimental Allergy	Excluded at Full-Text Screen	No 2nd dose data
Orhan, F. K. T., I.; Cakir, M.; Cihan, M.; Baki, A.; Dereci, S.	Anaphylaxis in children: A single-centre, 10-years retrospective, case study	2013	Allergy: European Journal of Allergy and Clinical Immunology	Excluded at Full-Text Screen	No 2nd dose data
Ponce Guevara, V. M. R., E.; Gonzalez Ruiz, A.; Munoz-Bellido, F.; Laffond Yges, E.; Davila Gonzalez, I.	Use of epinephrine in a tertiary hospital	2013	Allergy: European Journal of Allergy and Clinical Immunology	Excluded at Full-Text Screen	No 2nd dose data

Jiang, N. N. Y., J.; Wen, P. L.	Anaphylaxis in children and adults referred to a tertiary allergy clinic: A retrospective study of 1292 anaphylactic reactions	2013	Annals of Allergy, Asthma and Immunology	Excluded at Full-Text Screen	No 2nd dose data
Trepp, S. H., A.; Muller, U.	Emergency treatment of patients with hymenoptera venom allergy. Is the treatment in accordance with the guidelines?	2013	Respiration	Excluded at Full-Text Screen	No 2nd dose data
Ward, C. E. M., L.; Greenhawt, M. J.	Treatment of allergic reactions and quality of life among caregivers of food allergic children	2013	Journal of Allergy and Clinical Immunology	Excluded at Full-Text Screen	No 2nd dose data
Ben-Shoshan, M. V., S. L.; Eisman, H.; Alizadehfar, R.; Perkins, E.; Joseph, L.; Morris, J.; Clarke, A. E.	Anaphylaxis in children treated at the Montreal children's hospital: Rate, clinical characteristics, triggers and management	2013	Journal of Allergy and Clinical Immunology	Excluded at Full-Text Screen	No 2nd dose data
Fleming, J. C., S.; Camargo Jr, C.; Rudders, S. A.	Early treatment of food-induced allergic reactions with epinephrine is associated with lower risk of hospital admission	2013	Journal of Allergy and Clinical Immunology	Excluded at Full-Text Screen	No 2nd dose data
Rafeeq, M. R. D., M.	Food allergy and anaphylaxis-2037. Emergency department management of insect-sting allergic reactions in a community hospital in the United States	2013	World Allergy Organization Journal	Excluded at Full-Text Screen	No 2nd dose data
Vetander, M. H., D.; Flodstrom, C.; Ostblom, E.; Alfven, T.; Ly, D. H.; Hedlin, G.; Lilja, G.; Nilsson, C.; Wickman, M.	Anaphylaxis and reactions to foods in children - a population-based case study of emergency department visits	2012	Clinical and Experimental Allergy	Excluded at Full-Text Screen	No 2nd dose data
Santos, N. G., A.; Piedade, S.; Santa-Marta, C.; Pires, G.; Sampaio, G.; Borrego, L.; Arede, C.; Morais-Almeida, M.	Anaphylaxis in children and adolescents: A 1-year survey in an immunoallergy department	2012	Allergy: European Journal of Allergy and Clinical Immunology	Excluded at Full-Text Screen	No 2nd dose data
Clark, S. R., S.; Wei, W.; Camargo, C.	A retrospective database study of us patients treated in the emergency department (ED) or hospital with anaphylaxis: Factors associated with severeanaphylaxis	2012	Annals of Allergy, Asthma and Immunology	Excluded at Full-Text Screen	No 2nd dose data
Chouksey, A. S., D.; Puri, P.; Swamy, K.	Management of anaphylaxis: Are we compliant with the national guidelines	2012	Annals of Allergy, Asthma and Immunology	Excluded at Full-Text Screen	No 2nd dose data
Landsman-Blumberg, P. B. W., W.; Douglas, D.; Smith, D.; Camargo, C. A.	A retrospective database study of us children in the emergency department or hospital with food-induced anaphylaxis: Concordance with recommended post-discharge care	2012	Journal of Allergy and Clinical Immunology	Excluded at Full-Text Screen	No 2nd dose data
Boyle, J. C., C. A.; Lieberman, P.; Sampson, H.; Schwartz, L. B.; R. Simons F.E; Zitt, M.; Wilkinson, M.; Collins, C.; Tringale, M.; Wood, R.	Anaphylaxis in america-results from a national telephone survey	2012	Journal of Allergy and Clinical Immunology	Excluded at Full-Text Screen	No 2nd dose data
Raie, A. M., S.; Heffler, E.; Cadario, G.; Galimberti, M.; Rolla, G.	Food anaphylaxis: Data from registry of Center for Severe Allergic Reactions of Piemonte region (Italy)	2011	Clinical and Translational Allergy	Excluded at Full-Text Screen	No 2nd dose data
Raie, A. M., S.; Nebiolo, F.; Cadario, G.; Galimberti, M.; Heffler, E.; Rolla, G.	Epidemiology of anaphylaxis in Piemonte, Italy: Data from the regional registry of severe allergic reactions	2011	Allergy: European Journal of Allergy and Clinical Immunology	Excluded at Full-Text Screen	No 2nd dose data
Masumoto, N. S., R.; Yohei, A.; Yuko, A.; Yoshitaka, M.; Naohiko, T.; Yoko, M.; Motomura, C.; Honjo, S.; Kenji, O.; Hiroshi, O.	Immediate food-allergic children visited to our hospital emergency room	2011	Allergy: European Journal of Allergy and Clinical Immunology	Excluded at Full-Text Screen	No 2nd dose data

Cortes Alvarez, N. R. F., L.; Piza Oliveras, A.; May Llanas, E.; Margarit Mallol, J.	Management of anaphylaxis in a pediatric emergency department	2011	Allergy: European Journal of Allergy and Clinical Immunology	Excluded at Full-Text Screen	No 2nd dose data
Takeishi, D. K., T.; Utsunomiya, T.; Sato, S.; Imai, T.; Tomikawa, M.; Syukuya, A.; Ebisawa, M.	Oral challenge tests for sesame in Japan, A summary of 91 cases	2011	Allergy: European Journal of Allergy and Clinical Immunology	Excluded at Full-Text Screen	No 2nd dose data
Mietta, S. R., A.; Nebiolo, F.; Cadario, G.; Galimberti, M.; Rolla, G.	Food anaphylaxis in Piemonte Region (ITALY): Data from the regional registry of severe allergic reactions	2011	Allergy: European Journal of Allergy and Clinical Immunology	Excluded at Full-Text Screen	No 2nd dose data
Ly, D.	Readmittance of children to hospital emergency departments due to repeated reactions to food in Stockholm county-change in severity at re-exposure	2011	Allergy: European Journal of Allergy and Clinical Immunology	Excluded at Full-Text Screen	No 2nd dose data
Orhan, F. B. B., A.; Yilmaz, O.; Reisli, I.; Cakir, M.; Karakas, T.; Yuksel, H.	Anaphylaxis in pediatric patients: A multi-center, retrospective, case study	2009	Allergy: European Journal of Allergy and Clinical Immunology	Excluded at Full-Text Screen	No 2nd dose data
Le, T. V. H., E.; Pasmans, S.; Bruijnzeel-Koomen, C.; Knulst, A.	Suboptimal management of acute foodallergic reactions by patients, emergency departments and general practitioners	2009	Allergy: European Journal of Allergy and Clinical Immunology	Excluded at Full-Text Screen	No 2nd dose data
Rudders, S. A. B., A.; Vassallo, M. F.; Clark, S.; Camargo, C. A.	Trends in pediatric emergency department visits for food-induced anaphylaxis	2010	Journal of Allergy and Clinical Immunology	Excluded at Full-Text Screen	No 2nd dose data
Ben-Shoshan, M. N., L. N.; Alizadehfar, R.; Soller, L.; Fragapane, J.; Joseph, L.; St Pierre, Y.; Harada, L.; Fortin, C.; Allen, M.; Clarke, A.	Treatment of allergic reactions to peanut in recent versus initial reaction	2010	Allergy, Asthma and Clinical Immunology	Excluded at Full-Text Screen	No 2nd dose data
Helbling, A. M., U.; Hausmann, O.	Anaphylaxis - Reality of acute therapy and preventive measures. Analysis of 54 patients in a spezialized city hospital	2009	Allergologie	Excluded at Full-Text Screen	No 2nd dose data
De Silva, I. L. M., S. S.; Tey, D.; Tang, M. L. K.	Paediatric anaphylaxis: A 5 year retrospective review	2008	Allergy: European Journal of Allergy and Clinical Immunology	Excluded at Full-Text Screen	No 2nd dose data
Peng, M. M. J., H.	A Population-Based Study of the Incidence, Cause, and Severity of Anaphylaxis in the United Kingdom	2004	Archives of Internal Medicine	Excluded at Full-Text Screen	No 2nd dose data
Dobbie, A. R., C. M.	Provision of self-injectable adrenaline for children at risk of anaphylaxis: Its source, frequency and appropriateness of use, and effect	1998	Ambulatory Child Health	Excluded at Full-Text Screen	Exclude for other reasons
Bresser, H. S., C.; Rakoski, J.	Emergencies by insect stings in Munich in 1992	1995	Allergo Journal	Excluded at Full-Text Screen	No 2nd dose data
Ganapathy, S. L., Zaw; Ting, Daniel Ha; Goh, Lynette Sh; Chong, Shu Ling	Anaphylaxis in Children: Experience of 485 Episodes in 1,272,482 Patient Attendances at a Tertiary Paediatric Emergency Department from 2007 to 2014	2016	Annals of the Academy of Medicine, Singapore	Excluded at Full-Text Screen	No 2nd dose data
Manuyakorn, W. B., Suwat; Kamchaisatian, Wasu; Vilaiyuk, Soamarat; Sasisakulporn, Cherapat; Jotikasthira, Wanlapa	Pediatric anaphylaxis: triggers, clinical features, and treatment in a tertiary-care hospital	2015	Asian Pacific journal of allergy and immunology	Excluded at Full-Text Screen	No 2nd dose data
Gaspar, A. S., N.; Piedade, S.; Santa-Marta, C.; Pires, G.; Sampaio, G.; Arede, C.; Borrego, L. M.; Morais-Almeida, M.	One-year survey of paediatric anaphylaxis in an allergy department	2015	European annals of allergy and clinical immunology	Excluded at Full-Text Screen	No 2nd dose data

Alvarez-Perea, A. T.-P., M.; Martinez-Lezcano, P.; Marco, G.; Perez, D.; Zubeldia, J. M.; Baeza, M. L.	Anaphylaxis in Adolescent/Adult Patients Treated in the Emergency Department: Differences Between Initial Impressions and the Definitive Diagnosis	2015	Journal of investigational allergology & clinical immunology	Excluded at Full-Text Screen	No 2nd dose data
Asai, Y. Y., Yarden; Clarke, Ann; La Vieille, Sebastian; Delaney, J. Scott; Alizadehfar, Reza; Joseph, Lawrence; Mill, Christopher; Morris, Judy; Ben-Shoshan, Moshe	Rate, triggers, severity and management of anaphylaxis in adults treated in a Canadian emergency department	2014	International archives of allergy and immunology	Excluded at Full-Text Screen	No 2nd dose data
Chan, C.-F. C., Po-Hon; Huang, Ching-Feng; Wu, Tzee-Chung	Emergency department visits for food allergy in Taiwan: a retrospective study	2014	Pediatrics and neonatology	Excluded at Full-Text Screen	No 2nd dose data
Wood, R. A. C., Carlos A., Jr.; Lieberman, Philip; Sampson, Hugh A.; Schwartz, Lawrence B.; Zitt, Myron; Collins, Charlotte; Tringale, Michael; Wilkinson, Marilyn; Boyle, John; Simons, F. Estelle R.	Anaphylaxis in America: the prevalence and characteristics of anaphylaxis in the United States	2014	The Journal of allergy and clinical immunology	Excluded at Full-Text Screen	No 2nd dose data
Vetander, M. L., D. H.; Hakansson, N.; Lilja, G.; Nilsson, C.; Ostblom, E.; Wickman, M.; Bergstrom, A.	Recurrent reactions to food among children at paediatric emergency departments: epidemiology of allergic disease	2014	Clinical and experimental allergy : journal of the British Society for Allergy and Clinical Immunology	Excluded at Full-Text Screen	No 2nd dose data
Landsman-Blumberg, P. B. W., Wenhui; Douglas, Damon; Smith, David M.; Clark, Sunday; Camargo, Carlos A., Jr.	Food-induced anaphylaxis among commercially insured US adults: patient concordance with postdischarge care guidelines	2013	The journal of allergy and clinical immunology. In practice	Excluded at Full-Text Screen	No 2nd dose data
Calderon, E. M., Javier; Nazario, Sylvette	Anaphylaxis diagnosis and treatment at an emergency department in Puerto Rico	2013	Puerto Rico health sciences journal	Excluded at Full-Text Screen	No 2nd dose data
Khan, N. U. S., N.; Makda, A.; Mallick, A. S.; Ali Memon, M.; Hashmi, S. H.; Khan, U. R.; Razzak, J. A.	Anaphylaxis: incidence, presentation, causes and outcome in patients in a tertiary-care hospital in Karachi, Pakistan	2013	QJM : monthly journal of the Association of Physicians	Excluded at Full-Text Screen	No 2nd dose data
Rudders, S. A. C., Sunday; Wei, Wenhui; Camargo, Carlos A., Jr.	Longitudinal study of 954 patients with stinging insect anaphylaxis	2013	Annals of allergy, asthma & immunology : official publication of the American College of Allergy, Asthma, & Immunology	Excluded at Full-Text Screen	No 2nd dose data
Vezir, E. E., Mustafa; Kaya, Aysenur; Toyran, Muge; Ozcan, Celal; Akan, Aysegul; Azkur, Dilek; Ginis, Tayfur; Civelek, Ersoy; Kocabas, Can Naci	Characteristics of anaphylaxis in children referred to a tertiary care center	2013	Allergy and asthma proceedings	Excluded at Full-Text Screen	No 2nd dose data
Nagano, C. I., Akira; Yotani, Nobuyuki; Sakai, Hirokazu; Fujiwara, Takeo; Ohya, Yukihiro	(Anaphylaxis and biphasic reaction in a children hospital]	2013	Arerugi = (Allergy]	Excluded at Full-Text Screen	No 2nd dose data
Gelincik, A. D., Mustafa; Yilmaz, Emre; Ertek, Belkis; Erdogdu, Derya; Colakoglu, Bahattin; Buyukozturk, Suna	Anaphylaxis in a tertiary adult allergy clinic: a retrospective review of 516 patients	2013	Annals of allergy, asthma & immunology : official publication of the American College of Allergy, Asthma, & Immunology	Excluded at Full-Text Screen	No 2nd dose data
Jacobs, T. S. G., Matthew J.; Hauswirth, David; Mitchell, Lynda; Green, Todd D.	A survey study of index food-related allergic reactions and anaphylaxis management	2012	Pediatric allergy and immunology : official publication of the European Society of Pediatric Allergy and Immunology	Excluded at Full-Text Screen	No 2nd dose data

Nguyen-Luu, N. U. B.-S., Moshe; Alizadehfar, Reza; Joseph, Lawrence; Harada, Laurie; Allen, Mary; St-Pierre, Yvan; Clarke, Ann	Inadvertent exposures in children with peanut allergy	2012	Pediatric allergy and immunology : official publication of the European Society of Pediatric Allergy and Immunology	Excluded at Full-Text Screen	No 2nd dose data
Sole, D. I., J. C.; Borges, M. S.; Coelho, M. A.; Rosario, N. A.; Ardusso, L.; Bernd, L. A. G.; Latin American Anaphylaxis Working, Group	Anaphylaxis in Latin American children and adolescents: the Online Latin American Survey on Anaphylaxis (OLASA)	2012	Allergologia et immunopathologia	Excluded at Full-Text Screen	No 2nd dose data
Silva, R. G., E.; Cunha, L.; Falcao, H.	Anaphylaxis in children: a nine years retrospective study (2001-2009)	2012	Allergologia et immunopathologia	Excluded at Full-Text Screen	No 2nd dose data
Orhan, F. C., Y.; Bakirtas, A.; Yilmaz, O.; Boz, A. B.; Can, D.; Kuyucu, S.; Harmanci, K.; Tahan, F.; Reisli, I.; Karakas, T.; Baki, A.; Cokugras, H.; Cakir, M.; Yuksel, H.	Anaphylaxis in Turkish children: a multi-centre, retrospective, case study	2011	Clinical and experimental allergy : journal of the British Society for Allergy and Clinical Immunology	Excluded at Full-Text Screen	No 2nd dose data
Lertnawapan, R. M.-a.-n., Wirach	Anaphylaxis and biphasic phase in Thailand: 4-year observation	2011	Allergology international : official journal of the Japanese Society of Allergology	Excluded at Full-Text Screen	Exclude for other reasons
Hoffer, V. S., O.; Marcus, N.; Levy, Y.; Segal, N.; Lagovsky, I.; Monselise, Y.; Garty, B. Z.	Anaphylaxis in Israel: experience with 92 hospitalized children	2011	Pediatric allergy and immunology : official publication of the European Society of Pediatric Allergy and Immunology	Excluded at Full-Text Screen	No 2nd dose data
Techapornroong, M. A., Krittapoom; Cheungpasitporn, Wisit; Ruxrungtham, Kiat	Anaphylaxis: a ten years inpatient retrospective study	2010	Asian Pacific journal of allergy and immunology	Excluded at Full-Text Screen	No 2nd dose data
Rudders, S. A. B., Aleena; Corel, Blanka; Clark, Sunday; Camargo, Carlos A., Jr.	Multicenter study of repeat epinephrine treatments for food-related anaphylaxis	2010	Pediatrics	Excluded at Full-Text Screen	No 2nd dose data
Russell, S. M., Kathy; Losek, Joseph D.	Anaphylaxis management in the pediatric emergency department: opportunities for improvement	2010	Pediatric emergency care	Excluded at Full-Text Screen	No 2nd dose data
Piromrat, K. C., Sasawan; Trathong, Sommai	Anaphylaxis in an emergency department: a 2-year study in a tertiary-care hospital	2008	Asian Pacific journal of allergy and immunology	Excluded at Full-Text Screen	No 2nd dose data
Melville, N. B., T.	Paediatric allergic reactions in the emergency department: a review	2008	Emergency medicine journal : EMJ	Excluded at Full-Text Screen	No 2nd dose data
Jirapongsananuruk, O. B., Wicharn; Piyaphanee, Nuntawan; Visitsunthorn, Nualanong; Thongngarm, Torpong; Vichyanond, Pakit	Features of patients with anaphylaxis admitted to a university hospital	2007	Annals of allergy, asthma & immunology : official publication of the American College of Allergy, Asthma, & Immunology	Excluded at Full-Text Screen	No 2nd dose data
Braganza, S. C. A., J. P.; McKinnon, D. R. L.; Peake, J. E.; Brown, A. F. T.	Paediatric emergency department anaphylaxis: different patterns from adults	2006	Archives of disease in childhood	Excluded at Full-Text Screen	No 2nd dose data
Mehl, A. W., U.; Niggemann, B.	Anaphylactic reactions in children--a questionnaire-based survey in Germany	2005	Allergy	Excluded at Full-Text Screen	No 2nd dose data
Clark, S. L., Aidan A.; Gaeta, Theodore J.; Camargo, Carlos A., Jr.	Multicenter study of emergency department visits for insect sting allergies	2005	The Journal of allergy and clinical immunology	Excluded at Full-Text Screen	Exclude for other reasons

Cianferoni, A. N., Elio; Pucci, Neri; Lombardi, Enrico; Bernardini, Roberto; Vierucci, Alberto	Anaphylaxis: a 7-year follow-up survey of 46 children	2004	Annals of allergy, asthma & immunology : official publication of the American College of Allergy, Asthma, & Immunology	Excluded at Full-Text Screen	No 2nd dose data
Clark, S. B., S. Allan; Gaeta, Theodore J.; Brenner, Barry E.; Cydulka, Rita K.; Camargo, Carlos A.; Multicenter Airway Research Collaboration, Investigators	Multicenter study of emergency department visits for food allergies	2004	The Journal of allergy and clinical immunology	Excluded at Full-Text Screen	No 2nd dose data
Brown, A. F. M., D.; Chu, K.	Emergency department anaphylaxis: A review of 142 patients in a single year	2001	The Journal of allergy and clinical immunology	Excluded at Full-Text Screen	No 2nd dose data
Cianferoni, A. N., E.; Mugnaini, L.; Lombardi, E.; Bernardini, R.; Pucci, N.; Vierucci, A.	Clinical features of acute anaphylaxis in patients admitted to a university hospital: an 11-year retrospective review (1985-1996)	2001	Annals of allergy, asthma & immunology : official publication of the American College of Allergy, Asthma, & Immunology	Excluded at Full-Text Screen	No 2nd dose data
Pucar, F. K., R.; Lim, H.; Clarke, A. E.	Peanut challenge: a retrospective study of 140 patients	2001	Clinical and experimental allergy : journal of the British Society for Allergy and Clinical Immunology	Excluded at Full-Text Screen	No 2nd dose data
Lee, J. M. G., D. S.	Biphasic anaphylactic reactions in pediatrics	2000	Pediatrics	Excluded at Full-Text Screen	No 2nd dose data
Korenblat, P. L., M. J.; Dankner, R. E.; Day, J. H.	A retrospective study of epinephrine administration for anaphylaxis: how many doses are needed?	1999	Allergy and asthma proceedings	Excluded at Full-Text Screen	Exclude for other reasons
Stewart, A. G. E., P. W.	The incidence, aetiology and management of anaphylaxis presenting to an accident and emergency department	1996	QJM : monthly journal of the Association of Physicians	Excluded at Full-Text Screen	No 2nd dose data
Kemp, S. F. L., R. F.; Wolf, B. L.; Lieberman, P.	Anaphylaxis. A review of 266 cases	1995	Archives of internal medicine	Excluded at Full-Text Screen	No 2nd dose data
Civelek, E. E., M.; Akan, A.; Ozcan, C.; Kaya, A.; Vezir, E.; Ginis, T.; Azkur, D.; Toyran, M.; Tokac, M.; Kocabas, C. N.	The etiology and clinical features of anaphylaxis in a developing country: A nationwide survey in turkey	2017	Asian Pacific Journal of Allergy and Immunology	Excluded at Full-Text Screen	No 2nd dose data
Rudders, S. A. C., S.; Camargo, C. A.	Inpatient interventions are infrequent during pediatric hospitalizations for food-induced anaphylaxis	2017	Journal of Allergy and Clinical Immunology: In Practice	Excluded at Full-Text Screen	No 2nd dose data
van der Valk, J. P. M. B., I.; Gerth van Wijk, R.; Arends, N. J. T.; van Maaren, M. S.; de Groot, H.; Wichers, H. J.; Emons, J. A. M.; Dubois, A. E. J.; de Jong, N. W.	Small percentage of anaphylactic reactions treated with epinephrine during food challenges in Dutch children	2018	Annals of Allergy, Asthma and Immunology	Excluded at Full-Text Screen	No 2nd dose data
Kennard, L. T., I.; Rutkowski, K.; Azzu, V.; Yong, P. F. K.; Kasternow, B.; Hunter, H.; Cabdi, N. M. O.; Nakonechna, A.; Wagner, A.	A Multicenter Evaluation of Diagnosis and Management of Omega-5 Gliadin Allergy (Also Known as Wheat-Dependent Exercise-Induced Anaphylaxis) in 132 Adults	2018	Journal of Allergy and Clinical Immunology: In Practice	Excluded at Full-Text Screen	No 2nd dose data
Ito, K. O., M.; Kando, N.; Matsui, T.; Nakagawa, T.; Sugiura, S.; Ebisawa, M.	Surveillance of the use of adrenaline auto-injectors in Japanese children	2018	Allergology International	Excluded at Full-Text Screen	No 2nd dose data
Al Enezi, M. L., G.; Fox, A. T.; Anagnostou, K.	Safety and allergic reaction profiles of children undergoing baked milk and egg challenges: a 6-year experience from a pediatric tertiary referral center	2018	Journal of Allergy and Clinical Immunology: In Practice	Excluded at Full-Text Screen	No 2nd dose data

Kim, S. Y. K., M. H.; Cho, Y. J.	Different clinical features of anaphylaxis according to cause and risk factors for severe reactions	2018	Allergology International	Excluded at Full-Text Screen	No 2nd dose data
Sasaki, M. K., J. J.; Dharmage, S. C.; Field, M. J.; Sawyer, S. M.; McWilliam, V.; Peters, R. L.; Gurrin, L. C.; Vuillermin, P. J.; Douglass, J.; Pezic, A.; Brewerton, M.; Tang, M. L. K.; Patton, G. C.; Allen, K. J.	Prevalence of clinic-defined food allergy in early adolescence: The SchoolNuts study	2018	Journal of Allergy and Clinical Immunology	Excluded at Full-Text Screen	No 2nd dose data
Zahabi, S. C., A.; Gabrielli, S.; Moissan, J.; Eisman, H.; Morris, J.; Chan, E. S.; Shand, G.; Shoshan, M. B.	Venom triggered anaphylaxis cases management and clinical characteristics in Canada	2017	Allergy, Asthma and Clinical Immunology	Excluded at Full-Text Screen	No 2nd dose data
Mevius, H. W., M.; Vriesman, W.	Pediatric anaphylaxis in the Emergency Department: Incidence, provocative factors and use of epinephrine	2018	Clinical and Translational Allergy	Excluded at Full-Text Screen	No 2nd dose data
Aviles, B. G. L., N. M.; Perez, T. T.; Gil, L. M.; Posada, C. M.; Caballero, M. C.; Rovira, P. M.; Almarcha, T. A.; Arnau, M. J. F.; Toro, C. G.; Garde, J. G.	Review of oral food challenge tests in children	2018	Clinical and Translational Allergy	Excluded at Full-Text Screen	No 2nd dose data
Adeli, M. A., K.; AlShami, A.; Nisar, S.	Etiology and characteristics of patients presenting with anaphylaxis to the Pediatric Emergency Centers in Qatar	2018	Clinical and Translational Allergy	Excluded at Full-Text Screen	No 2nd dose data
Geddes, K. R. R., N.	Recognition of paediatric anaphylaxis-an audit of allergy admissions to our emergency department	2018	Archives of Disease in Childhood	Excluded at Full-Text Screen	No 2nd dose data
Le, M. C., A. E.; Eisman, H.; Morris, J.; Gravel, J.; Chan, E. S.; Lim, R.; O'Keefe, A.; Shand, G.; Ben-Shoshan, M.	Emergency management of pediatric anaphylaxis due to an unknown cause: A 5-year follow-up study in Canada	2018	Journal of Allergy and Clinical Immunology	Excluded at Full-Text Screen	No 2nd dose data
Alshami, A. A., M.; Alyafei, K.; Nisar, S.	Anaphylaxis presenting to the pediatric emergency centers in Qatar	2018	Journal of Allergy and Clinical Immunology	Excluded at Full-Text Screen	No 2nd dose data
Ruiz, J. C. Z., V.; Ben-Shoshan, M.; Tariq, H.; Sheppard, T.; Asai, Y.; Adatia, A.; Yanishevsky, Y.; Chan, E. S.; Shand, G.; Chin, R.; Clarke, A. E.	Cross-Canada anaphylaxis registry (C-CARE): Comparing rates, triggers, and management of anaphylaxis in a single centre emergency department (ED) over 4 years	2018	Journal of Allergy and Clinical Immunology	Excluded at Full-Text Screen	No 2nd dose data
Lee, J. S. W., M.	Characteristics of anaphylaxis and management in a midwestern hospital system	2018	Journal of Allergy and Clinical Immunology	Excluded at Full-Text Screen	No 2nd dose data
Chooniedass, R. T., B.; Martin, D.; Becker, A.	Reflections on the use of epinephrine for anaphylaxis	2017	Allergy, Asthma and Clinical Immunology	Excluded at Full-Text Screen	No 2nd dose data
Brooks, C. C., A.; Erwin, E.; Mikhail, I.	Diagnosis and treatment of food allergic reactions in pediatric emergency settings	2017	Annals of Allergy, Asthma and Immunology	Excluded at Full-Text Screen	No 2nd dose data
Nabavi, M. L., M.; Arshi, S.; Bemanian, M. H.; Esmaeilzadeh, H.; Molatefi, R.; Rekabi, M.; Ahmadian, J.; Eslami, N.; Shokri, S.; Darabi, K.; Sedighi, G. R.; Fallahpour, M.	Characteristics, etiology and treatment of pediatric and adult anaphylaxis in Iran	2017	Iranian Journal of Allergy, Asthma and Immunology	Excluded at Full-Text Screen	No 2nd dose data

Feuille, E. L., C.; Volel, C.; Sicherer, S. H.; Wang, J.	Time Trends in Food Allergy Diagnoses, Epinephrine Orders, and Epinephrine Administrations in New York City Schools	2017	Journal of Pediatrics	Excluded at Full-Text Screen	No 2nd dose data
Akuete, K. G., D.; Israelsen, R. B.; Broyles, J. M.; Higgins, L. J.; Green, T. D.; Naimi, D. R.; MacGinnitie, A. J.; Vitalpur, G.; Minard, C. G.; Davis, C. M.	Multicenter prevalence of anaphylaxis in clinic-based oral food challenges	2017	Annals of Allergy, Asthma and Immunology	Excluded at Full-Text Screen	No 2nd dose data
Ali, F.	A Survey of Self-Reported Food Allergy and Food-Related Anaphylaxis among Young Adult Students at Kuwait University, Kuwait	2017	Medical Principles and Practice	Excluded at Full-Text Screen	No 2nd dose data
Robinson, M. G., M.; Stukus, D. R.	Factors associated with epinephrine administration for anaphylaxis in children before arrival to the emergency department	2017	Annals of Allergy, Asthma and Immunology	Excluded at Full-Text Screen	No 2nd dose data
Wright, C. D. L., M.; Lieberman, P. L.; Lieberman, J. A.	An analysis of anaphylaxis cases at a single pediatric emergency department during a 1-year period	2017	Annals of Allergy, Asthma and Immunology	Excluded at Full-Text Screen	No 2nd dose data
Golovin, B. C., O.; Ghidirimschi, A.; Bagrinovschi, M.	Incidence and management of anaphylaxis in prehospital	2017	Critical Care	Excluded at Full-Text Screen	No 2nd dose data
White, M. H., C.; Muniz, R.	Characteristics of anaphylactic events in schools: Results of the 2015-2016 EpiPen4Schools survey	2017	Annals of Allergy, Asthma and Immunology	Excluded at Full-Text Screen	No 2nd dose data
Sindher, S. L., A.; Tupa, D.; Andorf, S.; Purington, N.; Chinthrajah, R.; Nadeau, K.	Age-related variations in reactions during positive oral food challenge to peanut protein	2017	Annals of Allergy, Asthma and Immunology	Excluded at Full-Text Screen	No 2nd dose data
Azevedo, J. G., A.; Mota, I.; Correia, M.; Benito-Garcia, F.; Piedade, S.; Borrego, L. M.; Morais-Almeida, M.	Anaphylaxis induced by tree nuts in preschool age children	2017	Allergy: European Journal of Allergy and Clinical Immunology	Excluded at Full-Text Screen	No 2nd dose data
Topalusic, I. I. J., I.; Cavcic, A.; Navratil, M.	Anaphylaxis in children-our experience	2017	Allergy: European Journal of Allergy and Clinical Immunology	Excluded at Full-Text Screen	No 2nd dose data
Coskun, R. D., S.; Unal, D.; Olgac, M.; Gelincik, A.; Colakoglu, B.; Buyukozturk, S.	Factors influencing anaphylaxis severity	2017	Allergy: European Journal of Allergy and Clinical Immunology	Excluded at Full-Text Screen	No 2nd dose data
Tagaro, I. A. D. V., M. B.	Anaphylaxis management in the emergency department of a tertiary hospital in the Philippines	2017	Allergy: European Journal of Allergy and Clinical Immunology	Excluded at Full-Text Screen	No 2nd dose data
Fernandes, R. R., F.; Pita, J.; Faria, E.; Pereira, C.; Todo Bom, A.; Carrapatoso, I.	Anaphylaxis in a food allergy outpatient department-one year case series	2017	Allergy: European Journal of Allergy and Clinical Immunology	Excluded at Full-Text Screen	No 2nd dose data
White, M. V. M., R.; Herrem, C.; Silvia, S.; Hogue, S.	Anaphylactic triggers in schools: Results of the 2014-2015 EpiPen4Schools survey	2017	Journal of Allergy and Clinical Immunology	Excluded at Full-Text Screen	No 2nd dose data
Vickery, B. P. B., K.; Burks, A. W.; Casale, T. B.; Hourihane, J. O.; Jones, S. M.; Radwan, A.; Vereda, A.; Adelman, D. C.	Outcome of 583 entry double-blind placebo-controlled peanut challenges during screening for the palisade phase 3 oral immunotherapy trial	2017	Journal of Allergy and Clinical Immunology	Excluded at Full-Text Screen	Exclude for other reasons
Restivo, L. K. C., A. E.; Moissan, J.; Eisman, H.; Morris, J.; Chan, E. S.; Shand, G.; Shoshan, M. B.	Clinical characteristics, immediate treatment, and long-term management of venom allergic reactions presenting to canadian pediatric emergency departments and out-of-hospital emergency services	2017	Journal of Allergy and Clinical Immunology	Excluded at Full-Text Screen	No 2nd dose data

Das, D. R., A.; Clarke, A. E.; Moisan, J.; Chan, E. S.; Eisman, H.; Ben-Shoshan, M.	Longitudinal differences in treatment of anaphylaxis presenting to pre-hospital emergency services in rural quebec	2017	Journal of Allergy and Clinical Immunology	Excluded at Full-Text Screen	No 2nd dose data
Adatia, A. B.-S., M.; Clarke, A. E.; Chan, E. S.; Shand, G.; Delaney, S.; Yanishevsky, Y.	Rate and management of anaphylaxis in adult patients at a tertiary care centre emergency department	2017	Journal of Allergy and Clinical Immunology	Excluded at Full-Text Screen	No 2nd dose data
Klemans, R. J. L., T. M.; Sigurdsson, V.; Enters-Weijnen, C. F.; van Hoffen, E.; Bruijnzeel-Koomen, C. A.; Knulst, A. C.	Management of acute food allergic reactions by general practitioners	2013	Eur Ann Allergy Clin Immunol	Excluded at Full-Text Screen	No 2nd dose data
Lieberman, J. A. C., A. L.; Vitale, M.; Sampson, H. A.	Outcomes of office-based, open food challenges in the management of food allergy	2011	Journal of Allergy and Clinical Immunology	Excluded at Full-Text Screen	<10 cases anaphylaxis
Blackman, A. A., S.; Anagnostou, A.	Treatment of anaphylaxis: Are we doing it right?	2018	Journal of Allergy and Clinical Immunology	Excluded at Full-Text Screen	No 2nd dose data
Mostmans, Y. G., M.; Blykers, M.; Mols, P.; Naeije, N.; Gutermuth, J.	Adrenaline in anaphylaxis treatment and self-administration: experience from an inner city emergency department	2017	Allergy: European Journal of Allergy and Clinical Immunology	Excluded at Full-Text Screen	No 2nd dose data
O'Keefe, A. C., A.; St. Pierre, Y.; Mill, J.; Asai, Y.; Eisman, H.; La Vieille, S.; Alizadehfar, R.; Joseph, L.; Morris, J.; Gravel, J.; Ben-Shoshan, M.	The Risk of Recurrent Anaphylaxis	2017	Journal of Pediatrics	Excluded at Full-Text Screen	No 2nd dose data
Joshi, D. T., J.; Lales, G.; Pittsenbarger, Z.; Yarbrough, M.; Gupta, R. S.; Smith, B.	Disparities in pre-emergency department epinephrine and antihistamine use for anaphylaxis	2017	Journal of Allergy and Clinical Immunology	Excluded at Full-Text Screen	No 2nd dose data
Ko, B. S. K., W. Y.; Ryoo, S. M.; Ahn, S.; Sohn, C. H.; Seo, D. W.; Lee, Y. S.; Lim, K. S.; Kim, T. B.	Biphasic reactions in patients with anaphylaxis treated with corticosteroids	2015	Annals of Allergy, Asthma and Immunology	Excluded at Full-Text Screen	No 2nd dose data
Hitti, E. A. Z., F.; Harmouche, E.; Saliba, M.; Mufarrij, A.	Acute allergic reactions in the emergency department: Characteristics and management practices	2015	European Journal of Emergency Medicine	Excluded at Full-Text Screen	No 2nd dose data
Guzman, M. A., B.; Morales, C.; Zambrano, G.; Rodriguez, A.; Baeza, M. L.; Alvarez-Perea, A.	Characteristics of anaphylaxis in a pediatric emergency department	2015	Allergy: European Journal of Allergy and Clinical Immunology	Excluded at Full-Text Screen	No 2nd dose data
Yanishevsky, Y. C., A. E.; Vieille, S. L.; Delaney, S.; Alizadehfar, R.; Mill, C.; Joseph, L.; Morris, J.; Asai, Y.; Ben-Shoshan, M.	Managing anaphylaxis in adults: A review of all cases presenting in a single year at an emergency department	2014	Journal of Allergy and Clinical Immunology	Excluded at Full-Text Screen	No 2nd dose data
Aston, A., P. Turner, M. Ruiz-Garcia, R. Boyle and S. Brown	Assessing severity of peanut-allergic reactions during research food challenges	2016	World Allergy Organization Journal. Conference: 4th WAO International Scientific Conference, WISC	Excluded at Full-Text Screen	No 2nd dose data
Pouessel, G., V. Cerbelle, S. Lejeune, S. Leteurtre, N. Ramdane and A. Deschildre	Anaphylaxis admissions in pediatric intensive care units: Follow-up and risk of recurrence	2018	Pediatric allergy and immunology : official publication of the European Society of Pediatric Allergy and Immunology.	Excluded at Full-Text Screen	No 2nd dose data
Gabrielli, S., A. Clarke, J. Morris, H. Eisman, J. Gravel, P. Enarson, E. S. Chan, J. Gerdts, A. O'Keefe, R. Porter, R. Lim, Y. Yanishevsky, A. Adatia and M. Ben-Shoshan	Teenagers and those with severe reactions are more likely to use their epinephrine autoinjector in cases of anaphylaxis in Canada	2019	Journal of Allergy and Clinical Immunology: In Practice	Excluded at Full-Text Screen	No 2nd dose data

Reier-Nilsen, T., M. M. Michelsen, K. C. Lodrup Carlsen, K. H. Carlsen, P. Mowinckel, U. C. Nygaard, E. Namork, M. P. Borres and G. Haland	Feasibility of desensitizing children highly allergic to peanut by high-dose oral immunotherapy	2019	Allergy: European Journal of Allergy and Clinical Immunology	Excluded at Full-Text Screen	No 2nd dose data
Kauppila, T. K., M. Paassilta, A. K. Kukkonen, M. Kuitunen, A. S. Pelkonen and M. J. Makela	Outcome of oral immunotherapy for persistent cow's milk allergy from 11 years of experience in Finland	2019	Pediatric Allergy and Immunology.	Excluded at Full-Text Screen	No 2nd dose data
Andrew, E., Z. Nehme, S. Bernard and K. Smith	Pediatric Anaphylaxis in the Prehospital Setting: Incidence, Characteristics, and Management	2018	Prehospital emergency care : official journal of the National Association of EMS Physicians and the National Association of State EMS Directors	Excluded at Full-Text Screen	No 2nd dose data
Kawahara, T., J. Tezuka, T. Ninomiya, S. Honjo, N. Masumoto, M. Nanishi, H. Nakayama and S. Ohga	Risk prediction of severe reaction to oral challenge test of cow's milk	2019	European Journal of Pediatrics	Excluded at Full-Text Screen	No 2nd dose data
Cho, H., D. Kim, Y. Choo, J. Park, J. Choi, D. Jang, T. Kim, J. W. Jeong and J. W. Kwon	Common causes of emergency department visits for anaphylaxis in Korean community hospitals: A cross-sectional study	2019	Medicine	Excluded at Full-Text Screen	No 2nd dose data
Dubus, J. C., M. S. Le, J. Vitte, P. Minodier, A. Boutin, A. Carsin, G. Viudes and G. Noel	Use of epinephrine in emergency department depends on anaphylaxis severity in children	2019	European Journal of Pediatrics	Excluded at Full-Text Screen	No 2nd dose data
Capucilli, P., K. Kennedy, J. Lee, R. Grundmeier and J. M. Spergel	Accidental Food Reactions Requiring Emergency Department Evaluation in Children with Food Allergy are Rare	2019	Journal of Allergy and Clinical Immunology	Excluded at Full-Text Screen	No 2nd dose data
Ghrooda, M. A., M. Ben-Shoshan, A. E. Clarke, J. Moisan, B. Miles and G. Shand	Cross- Canada Anaphylaxis Registry: comparing triggers and management of anaphylaxis in emergency medical service in Quebec over a 4-year period	2019	Journal of Allergy and Clinical Immunology	Excluded at Full-Text Screen	No 2nd dose data
Dickson, M. A., C. W. Ng, K. Neupert and P. Varshney	Epinephrine Administration Trends in a Large Urban School District After Implementing Unassigned Epinephrine	2019	Journal of Allergy and Clinical Immunology	Excluded at Full-Text Screen	No 2nd dose data
Patel, N., M. Vazquez-Ortiz, A. Robb, M. H. Shamji, D. E. Campbell and P. J. Turner	Successful Desensitisation And Sustained Unresponsiveness Using Modified Peanut: Results From The BOPI Study	2019	Journal of Allergy and Clinical Immunology	Excluded at Full-Text Screen	No 2nd dose data
Knox, S. M. and B. C. Schroer	Use Of Emergency Resources During In Office Oral Food Challenges	2019	Journal of Allergy and Clinical Immunology	Excluded at Full-Text Screen	Multiple publications of dataset
Vickery, B. P., A. Vereda, T. B. Casale, K. Beyer, G. D. Toit, J. O. Hourihane, S. M. Jones, W. G. Shreffler, A. Marcantonio, R. Zawadzki, S. G. Dilly, D. C. Adelman and A. Wesley Burks	AR101 oral immunotherapy for peanut allergy	2018	New England Journal of Medicine	Excluded at Full-Text Screen	No 2nd dose data
Reynolds, C., L. Denny, S. Al-Badri and S. O'Connor	Management of anaphylaxis in a tertiary emergency department- A retrospective review	2018	Internal Medicine Journal	Excluded at Full-Text Screen	No 2nd dose data

Capucilli, P., A. Cianferoni, J. Fiedler, L. Gober, N. Pawlowski, G. Ram, R. Saltzman, J. M. Spergel and J. Heimall	Differences in egg and milk food challenge outcomes based on tolerance to the baked form	2018	Annals of Allergy, Asthma and Immunology	Excluded at Full-Text Screen	No 2nd dose data
Araki, M., Y. Hamahata, M. Usui and M. Akashi	Use of multiple doses of adrenaline for food-induced anaphylaxis. (Japanese]	2018	Japanese Journal of Allergology	Excluded at Full-Text Screen	No 2nd dose data
Kitamura, K., Y. Takasato, T. Matsui, S. Sugiura and K. Ito	A dosing interval of 40 minutes is favorable in comparison to 30 minutes for a safe oral food challenges with hen's egg and wheat	2018	Allergy: European Journal of Allergy and Clinical Immunology	Excluded at Full-Text Screen	No 2nd dose data
Pouessel, G., F. Chagnon, C. Trochu, J. Labreuche, S. Lejeune, M. Recher, A. Deschildre, S. Leteurtre, P. Tourneux, G. Boussicault, G. Thiriez, O. Brissaud, A. Garenne, M. Jokic, I. Petit, D. Semama, J. Bergounioux, T. Debillon, I. Wroblewski, A. Dorkenoo, E. Mallet, E. Javouhey, B. Vanel, R. Vialet, F. Michel, G. Cambonie, C. Milesi, N. Boussard, N. Joram, D. Dupont, P. Tissieres, S. Dauger, S. Renolleau, J. L. Leger, T. Mansir, J. P. Saulnier, N. Bednarek, O. Tirel, T. Blanc, H. Patural, D. Astruc, M. O. Marcoux, R. Amadieu and J. Chantreuil	Anaphylaxis admissions to pediatric intensive care units in France	2018	Allergy: European Journal of Allergy and Clinical Immunology	Excluded at Full-Text Screen	No 2nd dose data
Ruiz Oropeza, A., A. Lassen, S. Halken, C. Bindslev-Jensen and C. G. Mortz	Anaphylaxis in an emergency care setting: a one year prospective study in children and adults	2017	Scandinavian journal of trauma, resuscitation and emergency medicine	Excluded at Full-Text Screen	No 2nd dose data
Yoshida, N., H. Hirata, Y. Fukushima and K. Sugiyama	Survey on the proper use of anadrenaline autoinjectorin 551 Japanese outdoor workers after hymenopterastings	2018	Journal of Allergy and Clinical Immunology	Excluded at Full-Text Screen	No 2nd dose data
Adeli, M., K. Alyafei, S. I. Chaudhry and S. Nisar	Incidence, etiology and characteristics of adult onset anaphylaxis in Qatar	2018	Journal of Allergy and Clinical Immunology	Excluded at Full-Text Screen	No 2nd dose data
Vyas, D., B. Musaddaq, C. Gore and R. Boyle	Managing anaphylaxis in the emergency department-what can be done better?	2015	Clinical and Experimental Allergy	Excluded at Full-Text Screen	No 2nd dose data
Yoon, L., B. R. Kim, J. Y. Lee, K. Kim, Y. M. Kim, S. H. Kim and H. Y. Kim	Clinical features of anaphylaxis according to age in a single university hospital in Korea	2017	Asian Pacific Journal of Allergy and Immunology	Excluded at Full-Text Screen	No 2nd dose data
Misirlioglu, E. D., E. Vezir, M. Toyran, M. Capanoglu, H. Guvenir, E. Civelek and C. N. Kocabas	Clinical diagnosis and management of anaphylaxis in infancy	2017	Allergy and Asthma Proceedings	Excluded at Full-Text Screen	No 2nd dose data
Ruiz Oropeza, A., S. Mikkelsen, C. Bindslev-Jensen and C. G. Mortz	Pre-hospital treatment of bee and wasp induced anaphylactic reactions: a retrospective study	2017	Scandinavian journal of trauma, resuscitation and emergency medicine	Excluded at Full-Text Screen	No 2nd dose data
Kim, M., S. Kim, W. Song, Y. Cho and S. Chang	Different clinical features of anaphylaxis according to the cause	2017	Allergy: European Journal of Allergy and Clinical Immunology	Excluded at Full-Text Screen	No 2nd dose data
Sidhu, N., S. Jones, T. Perry, T. Thompson, E. Storm, M. S. Melguizo Castro and T. G. Nick	Evaluation of Anaphylaxis Management in a Pediatric Emergency Department	2016	Pediatric Emergency Care	Excluded at Full-Text Screen	No 2nd dose data

Ben Ghezala, H., N. Kouraichi, N. Brahmi, A. Bouguerba, H. Thabet and M. Amamou	Anaphylaxis in a medical intensive care unit: Experience of a Tunisian reference center. (French]	2016	Reanimation	Excluded at Full-Text Screen	No 2nd dose data
Dibek Misirlioglu, E., E. Vezir, M. Toyran, M. Capanoglu, H. Guvenir, E. Civelek and C. N. Kocabas	Anaphylaxis in children younger than 2 years old: Single center experience	2016	Allergy: European Journal of Allergy and Clinical Immunology	Excluded at Full-Text Screen	No 2nd dose data
Sabouraud-Leclerc, D., E. Beaudouin, A. Chabbert, C. Larue, M. D. Donnou, M. Boulegue, C. Nootens and D. A. Moneret-Vautrin	Food allergy in schools: A report on 56 cases observed by the allergovigilance network between 2005 and 2015. (French]	2015	Revue Francaise d'Allergologie	Excluded at Full-Text Screen	No 2nd dose data
Kim, C. W., J. H. Im and H. Y. Kwon	Characteristics of food-related anaphylaxis in adult: A single center experience	2015	Allergy: European Journal of Allergy and Clinical Immunology	Excluded at Full-Text Screen	No 2nd dose data
Garriga, T., M. Guilarte, L. Ferre, N. Moreno, E. Alcoceba, B. Delavalle, C. Gomez and V. Cardona	Dangers of adrenaline use in anaphylaxis: A multicentre register	2015	Allergy: European Journal of Allergy and Clinical Immunology	Excluded at Full-Text Screen	No 2nd dose data
Abul, M. H., F. Orhan, T. Karakas, Z. I. K. Topcu and A. Baki	Adherence to the treatment choices of anaphylaxis: An epidemiological view of the pediatric patients	2015	Journal of Allergy and Clinical Immunology	Excluded at Full-Text Screen	No 2nd dose data
Strothman, K., D. Scherzer, P. Mustillo and R. Scherzer	Inpatient management and discharge planning for children admitted for food-induced anaphylaxis	2015	Journal of Allergy and Clinical Immunology	Excluded at Full-Text Screen	No 2nd dose data
Oya, S., T. Nakamori and H. Kinoshita	Incidence and characteristics of biphasic and protracted anaphylaxis: Evaluation of 114 inpatients	2014	Acute Medicine and Surgery	Excluded at Full-Text Screen	No 2nd dose data
Katsunuma, T., K. Akashi and M. Watanabe	Anaphylaxis in children: Demographic and clinical features and triggers	2014	Allergy: European Journal of Allergy and Clinical Immunology	Excluded at Full-Text Screen	No 2nd dose data
Battisti, A., F. Lazzarotto, R. Bonaguro and A. Muraro	Anaphylaxis surveillance project in North Eastern Italy. Research grant 309/2009 Veneto Region	2014	Allergy: European Journal of Allergy and Clinical Immunology	Excluded at Full-Text Screen	No 2nd dose data
Goolsby, A. L., H. Minto, A. Perkins and K. M. Maples	Abbreviated oral food challenge as a safe and effective alternative for diagnosing food allergy in a pediatric clinic	2014	Journal of Allergy and Clinical Immunology	Excluded at Full-Text Screen	No 2nd dose data
Rueff, F.	Anaphylactic reactions as adverse effects of the allergen-specific immunotherapy. (German]	2013	Allergologie	Excluded at Full-Text Screen	No 2nd dose data
Fuzak, J. K. and J. Trainor	Comparison of the incidence, etiology, and management of anaphylaxis over time	2013	Pediatric Emergency Care	Excluded at Full-Text Screen	No 2nd dose data
Lauritano, E. C., A. Novi, M. C. Santoro and I. Casagranda	Incidence, clinical features and management of acute allergic reactions: the experience of a single, Italian Emergency Department	2013	European review for medical and pharmacological sciences	Excluded at Full-Text Screen	No 2nd dose data
Vazquez, M., J. Lozano, R. Jimenez, M. Alvaro, O. Dominguez and A. M. Plaza	Allergic reactions due to cow's milk (CM) doses and triggering factors in 40 CM anaphylactic children during maintenance phase of specific oral tolerance induction (SOTI) treatment to CM in our unit	2011	Clinical and Translational Allergy. Conference: Food Allergy and Anaphylaxis Meeting	Excluded at Full-Text Screen	No 2nd dose data
Dhariwal, M. and M. Rafeeq	Emergency department management of insectstingallergic reactions ina community hospital	2011	Annals of Allergy, Asthma and Immunology	Excluded at Full-Text Screen	No 2nd dose data
Wainstein, B. K., J. Studdert, M. Ziegler and J. B. Ziegler	Prediction of anaphylaxis during peanut food challenge: Usefulness of the peanut skin prick test (SPT) and specific IgE level	2010	Pediatric Allergy and Immunology	Excluded at Full-Text Screen	<10 cases anaphylaxis

Haynes, A., W. Watson, G. Rex and S. Kapur	Review of food challenges in a pediatric tertiary care centre	2010	Allergy, Asthma and Clinical Immunology. Conference: Canadian Society of Allergy and Clinical Immunology Annual Scientific Meeting	Excluded at Full-Text Screen	No 2nd dose data
Esteso, O., A. Sala Cunill, M. Guilarte, M. Labrador, O. Luengo and V. Cardona	A review of anaphylaxis management in the emergency room	2010	Allergy: European Journal of Allergy and Clinical Immunology	Excluded at Full-Text Screen	Exclude for other reasons
Rodgers, H., N. Ruth and N. Makwana	Analysis of children presenting to a Birmingham Hospital Trust with anaphylaxis	2009	Clinical and Experimental Allergy	Excluded at Full-Text Screen	No 2nd dose data
Noimark, L., G. A. Khakoo, A. Summerfield, J. Gardner, H. Cox and J. O. Warner	Use and availability of adrenline auto-injectors in event of anaphylaxis: A pilot study	2009	Clinical and Experimental Allergy	Excluded at Full-Text Screen	No 2nd dose data
Pourpak, Z., L. Ghojezadeh, M. Mansouri, H. Mozaffari and A. Farhoudi	Wheat anaphylaxis in children	2007	Immunological Investigations	Excluded at Full-Text Screen	No 2nd dose data
Enrique, E., F. Pineda, T. Malek, J. Bartra, M. Basagana, R. Tella, J. V. Castello, R. Alonso, J. A. De Mateo, T. Cerda-Trias, M. D. M. San Miguel-Moncin, S. Monzon, M. Garcia, R. Palacios and A. Cistero-Bahima	Sublingual immunotherapy for hazelnut food allergy: A randomized, double-blind, placebo-controlled study with a standardized hazelnut extract	2005	Journal of Allergy and Clinical Immunology	Excluded at Full-Text Screen	No 2nd dose data
Bidat, E., F. Rance, T. Baranes and S. Goulamhoussen	Goat's milk and sheep's milk allergies in children in the absence of cow's milk allergy. (French]	2003	Revue Francaise d'Allergologie et d'Immunologie Clinique	Excluded at Full-Text Screen	No 2nd dose data
Marguet, C., L. Couderc, T. Blanc, R. Amar, C. Leloet, D. Feray and E. Mallet	Anaphylaxis in children and adolescents: apropos of 44 patients aged 2 months to 15 years. (French]	1999	Archives de pediatrie : organe officiel de la Societe francaise de pediatrie	Excluded at Full-Text Screen	No 2nd dose data
Rangkakulnuwat, P., K. Sutham and M. Lao-Araya	Anaphylaxis: Ten-year retrospective study from a tertiary-care hospital in Northern Thailand	2018	Asian Pacific Journal of Allergy & Immunology	Excluded at Full-Text Screen	No 2nd dose data
Ghazali, H., M. Gammoudi, A. Yahmadi, G. Chaaebeni, A. Souyah and S. Souissi	Anaphylaxis in an emergency department: Epidemiology, clinical features and management	2017	Tunisie Medicale	Excluded at Full-Text Screen	No 2nd dose data
Blazowski, LJ., Kurzawa, R.,, Kuna, P., Majak, P.	Cluster analysis of 505 real-life food-induced anaphylaxis in children reveals two stable clinical phenotypes	2020	Allergy: European Journal of Allergy and Clinical Immunology.	Excluded at Full-Text Screen	No 2nd dose data
Chung, BY., Kim, JC., Kang, SY., Jung, MJ., Kim, HO., Park, CW.	Anaphylaxis: Five Years' Experience in the Emergency Rooms of Five University Hospitals in Korea	2020	Medicina	Excluded at Full-Text Screen	No 2nd dose data
De Vera, MJT.,	Anaphylaxis diagnosis and management in the Emergency Department of a tertiary hospital in the Philippines	2020	Asia Pacific Allergy	Excluded at Full-Text Screen	No 2nd dose data
S Dua, Bond, S.: Durham, S. R., Kimber, I., Mills, C., Roberts, G., Skypala, I., Wason, J., Ewan, P., Boyle, R., Clark, A.	Effect of sleep deprivation and exercise on reaction threshold in adults with peanut allergy: A randomized controlled study	2019	Journal of Allergy and Clinical Immunology	Excluded at Full-Text Screen	No relevant data or other reason

Edelman, SMK., Makela, M. J.	Eliciting allergens and treatment of anaphylaxis: Report of the Finnish national anaphylaxis registry	2019	Allergy: European Journal of Allergy and Clinical Immunology	Excluded at Full-Text Screen	No 2nd dose data
Frois, ATC.	Anaphylactic Reactions in the Emergency Department of a Portuguese Tertiary Hospital: Clinical Characterization and Disease Notification	2019	Acta Medica Portuguesa	Excluded at Full-Text Screen	<10 anaphylaxis cases
A. S. Gaspar, AS., Faria, E., Camara, R., Rodrigues-Alves, R., Carrapatoso, I., Gomes, E., Pereira, AM., Carneiro-Leao, L., Morais-Almeida, M., Delgado, L., Pedro, E., Branco-Ferreira, M.	Anaphylaxis in Portugal: 10-year SPAIC national survey 2007-2017. (Portuguese]	2019	Revista Portuguesa de Imunoalergologia	Excluded at Full-Text Screen	No relevant data or other reason
Kraft, MTS., Strothman, K., Moore-Clingenpeel, M., Grayson, MH.	M. T. S. Kraft, R.: Strothman, K.: Moore-Clingenpeel, M.: Grayson, M. H.	2019	Annals of Allergy, Asthma and Immunology	Excluded at Full-Text Screen	No relevant data or other reason
P. S. Rangkakulnuwat, K.: Lao-Araya, M.	Anaphylaxis: Ten-year retrospective study from a tertiary-care hospital in Asia	2020	Asian Pacific Journal of Allergy and Immunology	Excluded at Full-Text Screen	No 2nd dose data

I. Record Flow: Included References

The references included in the final systematic revie

Table S14: References included in final meta-analyses (N=76). References where additional information has been provided by study authors is also listed.

AUTHOR	TITLE	YR	JOURNAL	SCREENING OUTCOME	INFORMATION SOURCE
Abrams, E. M. C., N.; Becker, A. B.	Oral food challenge outcomes in a tertiary care allergy center	2012	Journal of Allergy and Clinical Immunology	INCLUDED	Full Text + author data
Alen Coutinho I, Ferreira D, Regateiro FS, Pita J, Ferreira M, Martins JF, Fonseca IA, Loureiro C, Todo-Bom A.	Anaphylaxis in an emergency department: a retrospective 10-year study in a tertiary hospital.	2019	Eur Ann Allergy Clin Immunol.	INCLUDED	Full Text
Alen Coutinho, I., J. Pita, M. Alves, C. Loureiro and A. Todo Bom	Pediatric anaphylaxis-A view from a tertiary hospital emergency department	2018	Allergy: European Journal of Allergy and Clinical Immunology	INCLUDED	Abstract + author data
Alqurashi, W. S., I.; Chan, K.; Neto, G.; Wells, G.	Epidemiology and clinical predictors of biphasic reactions in children with anaphylaxis	2014	Academic Emergency Medicine	INCLUDED	Full Text + author data
Anvari S, Blackman AC, Anagnostou A.	Insights into Infant Anaphylaxis	2019	J Allergy Clin Immunol Pract.	INCLUDED	Full Text + author data
Arana, N. G., J.; Bilbao, A.; Maruri, M.; Suinaga, M.; Escobal, M.	Anaphylaxis: A review of a series	2009	Allergy: European Journal of Allergy and Clinical Immunology	INCLUDED	Abstract
Arkwright, P. D.	Automatic epinephrine device use in children with food allergies	2009	The Journal of allergy and clinical immunology	INCLUDED	Full Text + author data
Asaumi, T., N. Yanagida, S. Sato, A. Shukuya, M. Nishino and M. Ebisawa	Provocation tests for the diagnosis of food-dependent exercise-induced anaphylaxis	2016	Pediatric Allergy and Immunology	INCLUDED	Full Text
Banerji, A. R., Susan A.; Corel, Blanka; Garth, Alisha M.; Clark, Sunday; Camargo, Carlos A., Jr.	Repeat epinephrine treatments for food-related allergic reactions that present to the emergency department	2010	Allergy and asthma proceedings	INCLUDED	Full Text
Ben-Shoshan, M. L. V., Sebastian; Eisman, Harley; Alizadehfar, Reza; Mill, Christopher; Perkins, Emma; Joseph, Lawrence; Morris, Judy; Clarke, Ann	Anaphylaxis treated in a Canadian pediatric hospital: Incidence, clinical characteristics, triggers, and management	2013	The Journal of allergy and clinical immunology	INCLUDED	Full Text
Brennan, A. K., S. U. Patil, J. Fleming, J. A. Boyce, E. S. Stieb, M. Nichols, R. S. Iyengar, P. Permaul, J. E. Walter, P. Hesterberg and W. G. Shreffler	Outcomes using a graded protocol for open food challenges	2013	Journal of Allergy and Clinical Immunology	INCLUDED	Abstract +author data
Brown, S. G. A. S., Shelley F.; Fatovich, Daniel M.; Burrows, Sally A.; Holdgate, Anna; Celenza, Antonio; Coulson, Adam; Hartnett, Leanne; Nagree, Yusuf; Cotterell, Claire; Isbister, Geoffrey K.	Anaphylaxis: clinical patterns, mediator release, and severity	2013	The Journal of allergy and clinical immunology	INCLUDED	Full Text + author data

Campbell, R. L. B., C. J.; Lee, S.; Bellamkonda, V. R.; Li, J. T. C.; Hagan, J. B.; Lohse, C. M.; Bellolio, M. F.	Predictors of repeat epinephrine administration for emergency department patients with anaphylaxis	2015	Journal of Allergy and Clinical Immunology: In Practice	INCLUDED	Full Text
Capps, J. A. S., V.; Arkwright, P. D.	Prevalence, outcome and pre-hospital management of anaphylaxis by first aiders and paramedical ambulance staff in Manchester, UK	2010	Resuscitation	INCLUDED	Full Text + author data
Cardona, V., L. Ferre-Ybarz, M. Guilarte, N. Moreno-Perez, C. Gomez-Galan, E. Alcoceba-Borras, M. B. Delavalle and T. Garriga-Baraut	Safety of Adrenaline Use in Anaphylaxis: a Multicentre Register	2017	International archives of allergy and immunology	INCLUDED	Full Text
Chung, T. G., L.; Vandenberghe, C.; Couperthwaite, S.; Sookram, S.; Liss, K.; Villa-Roel, C.; Rowe, B. H.	Pre-hospital management of anaphylaxis in one Canadian Urban Centre	2014	Resuscitation	INCLUDED	Full Text + author data
De Swert, L. F. A. B., Dominique; Raes, Marc; Dermaux, Anna-Maria	Anaphylaxis in referred pediatric patients: demographic and clinical features, triggers, and therapeutic approach	2008	European journal of pediatrics	INCLUDED	Full Text
Dibs, S. D. and M. D. Baker	Anaphylaxis in children: a 5-year experience	1997	Pediatrics	INCLUDED	Full Text
Dogru, M. B., I.; Ozmen, S.; Ginis, T.; Senol, H. D.	The features of anaphylaxis cases followed in the Pediatric allergy clinic	2017	Guncel Pediatri	INCLUDED	Full Text + author data
Dribin, T. E., K. A. Michelson, M. C. Monuteaux, A. M. Stack, K. S. Farbman, L. C. Schneider and M. I. Neuman	Identification of children with anaphylaxis at low risk of receiving acute inpatient therapies	2019	PLoS ONE	INCLUDED	Full Text
Elizur, A., M. Y. Appel, L. Nachshon, M. B. Levy, N. Epstein-Rigbi, K. Golobov and M. R. Goldberg	NUT Co Reactivity - ACquiring Knowledge for Elimination Recommendations (NUT CRACKER) study	2018	Allergy: European Journal of Allergy and Clinical Immunology	INCLUDED	Full Text + author data
Ellis, A. K. D., J. H.	Incidence and characteristics of biphasic anaphylaxis: A prospective evaluation of 103 patients	2007	Annals of Allergy, Asthma and Immunology	INCLUDED	Full Text + author data
Ewan, P. W.Clark, A. T.	Efficacy of a management plan based on severity assessment in longitudinal and case-controlled studies of 747 children with nut allergy: Proposal for good practice	2005	Clinical and Experimental Allergy	INCLUDED	Full Text
Ewan, P. W.Clark, A. T.	Good prognosis, clinical features, and circumstances of peanut and tree nut reactions in children treated by a specialist allergy center	2008	Journal of Allergy and Clinical Immunology	INCLUDED	Full Text
Ewan, P. W.Clark, A. T.	Long-term prospective observational study of patients with peanut and nut allergy after participation in a management plan	2001	Lancet	INCLUDED	Full Text
Farias Aquino, E. G. T. A., M. A.; Mugica Garica, M. V.; Moro Moro, M.; Vila Albelda, C.; Rosado Ingelmo, A.; Gomez Traseira, C.; Tobon Franco, J. D.	Factors for the use of one or more doses of adrenaline in anaphylaxis episodes attended in a general hospital	2013	Allergy: European Journal of Allergy and Clinical Immunology	INCLUDED	Abstract +author data
Gabrielli S, Clarke A, Morris J, Eisman H, Gravel J, Enarson P, Chan ES, O'Keefe A, Porter R, Lim R, Yanishevsky Y, Gerdts J,	Evaluation of Prehospital Management in a Canadian Emergency Department Anaphylaxis Cohort	2019	J Allergy Clin Immunol Pract.	INCLUDED	Full Text

Adatia A, LaVieille S, Zhang X, Ben-Shoshan M					
Giclas, H. E., M. L. Robinson, A. A. Phillips, C. B. Santos and B. J. Lanser	Comparison of Anaphylaxis Criteria with Outpatient Oral Food Challenge Outcomes	2019	Journal of Allergy and Clinical Immunology	INCLUDED	Abstract +author data
Goh, S. H. S., J. Y.; Loh, W.; Lee, K. P.; Tan, S. C.; Heng, W. J. K.; Ibrahim, I.; Lee, B. W.; Chiang, W. C.	Cause and clinical presentation of anaphylaxis in Singapore: From infancy to old age	2018	International Archives of Allergy and Immunology	INCLUDED	Full Text + author data
Gold, M. S. and R. Sainsbury	First aid anaphylaxis management in children who were prescribed an epinephrine autoinjector device (EpiPen)	2000	Journal of Allergy and Clinical Immunology	INCLUDED	Full Text
Grabenhenrich, L. B. D., S.; Rueff, F.; Renaudin, J. M.; Scherer, K.; Pfohler, C.; Treudler, R.; Koehli, A.; Mahler, V.; Spindler, T.; Lange, L.; Bilo, M. B.; Papadopoulos, N. G.; Hourihane, J. O. B.; Lang, R.; Fernandez-Rivas, M.; Christoff, G.; Cichocka-Jarosz, E.; Worm, M.	Epinephrine in Severe Allergic Reactions: The European Anaphylaxis Register	2018	J Allergy Clin Immunol Pract	INCLUDED	Full Text + author data
Hamilton, D. K.	Is Emergency Room Care After Home Use Of An Epinephrine Auto Injector Always Needed?	2019	Journal of Allergy and Clinical Immunology	INCLUDED	Abstract +author data
Hsiao, K. C. T., D.; Robinson, M.; Simmons, J.; Smart, J.	Safety of paediatric oral food challenges in a tertiary private hospital	2014	Internal Medicine Journal	INCLUDED	Abstract
Huang, F. C., Kanwaljit; Jarvinen, Kirsi M.; Nowak-Wegrzyn, Anna	Anaphylaxis in a New York City pediatric emergency department: triggers, treatments, and outcomes	2012	The Journal of allergy and clinical immunology	INCLUDED	Full Text
Inoue, N. and A. Yamamoto	Clinical evaluation of pediatric anaphylaxis and the necessity for multiple doses of epinephrine	2013	Asia Pacific Allergy	INCLUDED	Full Text + author data
Itazawa, T., K. Ito and E. Motohiro	Severe reaction during oral food challenges in children in a prospective multicenter study	2013	Allergy: European Journal of Allergy and Clinical Immunology	INCLUDED	Full Text + author data
Jarvinen, K. M. A., Sujitha; Shreffler, Wayne G.; Noone, Sally; Sicherer, Scott H.; Sampson, Hugh A.; Nowak-Wegrzyn, Anna	Epinephrine treatment is infrequent and biphasic reactions are rare in food-induced reactions during oral food challenges in children	2009	The Journal of allergy and clinical immunology	INCLUDED	Full Text
Jarvinen, K. M. S., Scott H.; Sampson, Hugh A.; Nowak-Wegrzyn, Anna	Use of multiple doses of epinephrine in food-induced anaphylaxis in children	2008	The Journal of allergy and clinical immunology	INCLUDED	Full Text
Johnson, J. M., Andrei; Alving, Kjell; Lidholm, Jonas; Borres, Magnus P.; Nordvall, Lennart	Ten-year review reveals changing trends and severity of allergic reactions to nuts and other foods	2014	Acta paediatrica (Oslo, Norway : 1992)	INCLUDED	Full Text + author data
Kim, M. Y., C. S. Park and J. W. Jeong	Management and educational status of adult anaphylaxis patients at emergency department	2018	Korean Journal of Internal Medicine	INCLUDED	Full Text
Kondo, A. I., K.; Nagasawa, H.; Takeuchi, I.; Jitsuiki, K.; Ohsaka, H.; Omori, K.; Yanagawa, Y.	An Analysis of Patients with Anaphylaxis Treated by a Physician-Staffed Helicopter	2018	Air Medical Journal	INCLUDED	Full Text

Lee, J. G., Jackie P. D.; Brown-Whitehorn, Terri; Spergel, Jonathan M.	Biphasic reactions in children undergoing oral food challenges	2013	Allergy and asthma proceedings	INCLUDED	Full Text + author data
Lee, M. and D. R. Stukus	Pre-hospital use of epinephrine for treatment of anaphylaxis in children and adolescents	2015	Journal of Allergy and Clinical Immunology	INCLUDED	Abstract +author data
Lee, S. P., A.; Lohse, C. M.; Hess, E. P.; Campbell, R. L.	Further Evaluation of Factors That May Predict Biphasic Reactions in Emergency Department Anaphylaxis Patients	2017	Journal of Allergy and Clinical Immunology: In Practice	INCLUDED	Full Text
Liu, X.Lee, S.Lohse, C. M.Hardy, C. T.Campbell, R. L.	Biphasic Reactions in Emergency Department Anaphylaxis Patients: A Prospective Cohort Study	2019	J Allergy Clin Immunol Pract	INCLUDED	Full Text
Manivannan, V. C., Ronna L.; Bellolio, M. Fernanda; Stead, Latha G.; Li, James T. C.; Decker, Wyatt W.	Factors associated with repeated use of epinephrine for the treatment of anaphylaxis	2009	Annals of allergy, asthma & immunology : official publication of the American College of Allergy, Asthma, & Immunology	INCLUDED	Full Text
Manivannan, V. H., Robert J.; Hankins, Daniel G.; Bellolio, M. Fernanda; Fedko, Martin G.; Decker, Wyatt W.; Campbell, Ronna L.	Epinephrine use and outcomes in anaphylaxis patients transported by emergency medical services	2014	The American journal of emergency medicine	INCLUDED	Full Text
Maris, I. H., J.; O'Sullivan, R.; Daly, D.	Prospective study of the incidence of anaphylaxis in Irish children	2015	Allergy: European Journal of Allergy and Clinical Immunology	INCLUDED	Abstract +author data
Mehr, S. Liew, W. K.Tey, D.Tang, M. L.	Clinical predictors for biphasic reactions in children presenting with anaphylaxis	2009	Clin Exp Allergy	INCLUDED	Full Text
Mulligan, K. D., G.; Campbell, T.; Campbell, D.; Mehr, S.	Open food challenges in the office: Are they safe?	2014	Internal Medicine Journal	INCLUDED	Abstract +author data
Nagakura, K. I., S. Sato, N. Yanagida, M. Nishino, T. Asaumi, K. Ogura and M. Ebisawa	Oral Immunotherapy in Japanese Children with Anaphylactic Peanut Allergy	2018	International Archives of Allergy and Immunology	INCLUDED	Full Text + author data
Nogic, C., J. Belousoff and D. Krieser	The diagnosis and management of children presenting with anaphylaxis to a metropolitan emergency department: A 2-year retrospective case series	2016	Journal of Paediatrics and Child Health	INCLUDED	Full Text
Noimark, L., G. Du Toit, C. Pasticaldi, D. Haddad, M. Alfaham, J. Gardner, W. Hyer, G. Vance, G. Roberts and J. Warner	Patient risk factors and treatment of anaphylaxis: A multi-site paediatric study	2010	Allergy: European Journal of Allergy and Clinical Immunology	INCLUDED	Full Text
Noone, S. R., J.; Sampson, H. A.; Wang, J.	Epinephrine Use in Positive Oral Food Challenges Performed as a Screening Test for Food Allergy Therapy Trials	2015	Journal of Allergy and Clinical Immunology: In Practice	INCLUDED	Full Text
Olabarri, M., S. Gonzalez-Peris, P. Vazquez, A. Gonzalez-Posada, N. Sanz, A. Vinuesa, N. Diez, J. Benito and S. Mintegi	Management of anaphylaxis in Spain: pediatric emergency care providers' knowledge	2018	European Journal of Emergency Medicine	INCLUDED	Abstract
Oren, E. B., Aleena; Clark, Sunday; Camargo, Carlos A., Jr.	Food-induced anaphylaxis and repeated epinephrine treatments	2007	Annals of allergy, asthma & immunology : official publication of the American College of Allergy, Asthma, & Immunology	INCLUDED	Full Text

Ponce Guevara, L. V., E. Laffond Yges, M. T. Gracia Bara, E. Moreno Rodilla, F. J. Munoz Bellido, M. Lazaro Sastre, E. M. Macias Iglesias, S. de Arriba Mendez, M. V. Campanon Toro and I. Davila Gonzalez	Adherence to anaphylaxis guidelines: Real-world data from the emergency department of a tertiary hospital	2018	Journal of Investigational Allergology and Clinical Immunology	INCLUDED	Full Text
Rudders, S. A. B., Aleena; Katzman, Daniel P.; Clark, Sunday; Camargo, Carlos A., Jr.	Multiple epinephrine doses for stinging insect hypersensitivity reactions treated in the emergency department	2010	Annals of allergy, asthma & immunology : official publication of the American College of Allergy, Asthma, & Immunology	INCLUDED	Full Text
Rudders, S. A.Geyer, B. C.Banerji, A.Phipatanakul, W.Clark, S.Camargo Jr, C. A.	Obesity is not a risk factor for repeat epinephrine use in the treatment of anaphylaxis	2012	Journal of Allergy and Clinical Immunology	INCLUDED	Full Text
Rueter, K. T., B.; Bear, N.; Lucas, M.; Borland, M. L.; Prescott, S. L.	Increased Use of Adrenaline in the Management of Childhood Anaphylaxis Over the Last Decade	2018	Journal of Allergy and Clinical Immunology: In Practice	INCLUDED	Full Text + author data
Soller, L., T. Teoh, I. Baerg, T. Wong, K. J. Hildebrand, V. E. Cook, C. M. Biggs, N. Lee, L. Yaworski, S. B. Cameron and E. S. Chan	Extended analysis of parent and child confidence in recognizing anaphylaxis and using the epinephrine autoinjector during oral food challenges	2019	Journal of Allergy and Clinical Immunology: In Practice	INCLUDED	Full Text + author data
Sundquist, B. K., J. Jose, D. Pauze, H. Wang and K. M. Jarvinen	Anaphylaxis risk factors for hospitalization and intensive care: A comparison between adults and children in an upstate New York emergency department	2019	Allergy and Asthma Proceedings	INCLUDED	Full Text + author data
Tiyyagura, G. K. A., L.; Cone, D. C.; Langhan, M.	Pediatric anaphylaxis management in the prehospital setting	2014	Prehospital emergency care : official journal of the National Association of EMS Physicians and the National Association of State EMS Directors	INCLUDED	Full Text
Topal, E. B., A.; Yilmaz, O.; Ertoy Karagol, I. H.; Arga, M.; Demirsoy, M. S.; Turktas, I.	Severe anaphylaxis in children: A single-center experience	2014	Pediatrics and Neonatology	INCLUDED	Full Text
Tsuang, Angela; Menon, Nikhil R.; Bahri, Natasha; Geyman, Lawrence S.; Nowak-Węgrzyn, Anna	Risk factors for multiple epinephrine doses in food-triggered anaphylaxis in children	2018	Annals of Allergy, Asthma & Immunology	INCLUDED	Full Text + author data
Turner, P. J. M., Sam; Joshi, Preeti; Tan, John; Wong, Melanie; Kakakios, Alyson; Campbell, Dianne E.	Safety of food challenges to extensively heated egg in egg-allergic children: a prospective cohort study	2013	Pediatric allergy and immunology : official publication of the European Society of Pediatric Allergy and Immunology	INCLUDED	Full Text + author data
Tyquin, B. F., L.; Hollinshead, K.; Mulligan, K.; Treloar, M.; Campbell, D.	Review of the use of adrenaline autoinjectors (aai) in nsw department of education schools in terms 1 and 2 2017	2017	Internal Medicine Journal	INCLUDED	Abstract +author data
Uguz, A. L., G.; Pumphrey, R.; Ewan, P.; Warner, J.; Dick, J.; Briggs, D.; Clarke, S.; Reading, D.; Hourihane, J.	Allergic reactions in the community: a questionnaire survey of members of the anaphylaxis campaign	2005	Clinical and experimental allergy : journal of the British Society for Allergy and Clinical Immunology	INCLUDED	Full Text
Van Der Valk, J. P. M. V. W., R. G.; Dubois, A. E. J.; De Groot, H.; Reitsma, M.; Vlieg-Boerstra, B.; Savelkoul, H. F. J.; Wichers, H. J.; De Jong, N. W.	Multicentre double-blind placebo-controlled food challenge study in children sensitised to cashew nut	2016	PLoS ONE	INCLUDED	Full Text

Vijaykumar, A. L., S.; Brathwaite, N.	Audit of anaphylaxis during food challenges at a paediatric day unit	2017	Clinical and Experimental Allergy	INCLUDED	Abstract
Villafana-Soto, K. M., S.; Joshi, P.; Wong, M.; Kakakios, A.; Campbell, D.	Safety of food challenges in a tertiary referral children's hospital	2011	Internal Medicine Journal	INCLUDED	Abstract
Webb, L. M. L., Phil	Anaphylaxis: a review of 601 cases	2006	Annals of allergy, asthma & immunology : official publication of the American College of Allergy, Asthma, & Immunology	INCLUDED	Full Text
White, M. V. H., Susan L.; Bennett, M. Elizabeth; Goss, Diana; Millar, Kimrey; Hollis, Kelly; Siegel, Peter H.; Wolf, Ray A.; Wooddell, Margaret J.; Silvia, Suyapa	EpiPen4Schools pilot survey: Occurrence of anaphylaxis, triggers, and epinephrine administration in a U.S. school setting	2015	Allergy and asthma proceedings	INCLUDED	Full Text
Yanagida, N. S., Sakura; Asaumi, Tomoyuki; Ogura, Kiyotake; Ebisawa, Motohiro	Risk Factors for Severe Reactions during Double-Blind Placebo-Controlled Food Challenges	2017	International archives of allergy and immunology	INCLUDED	Full Text + author data
Yanagida, N., S. Sato, K. Takahashi, K. Nagakura, T. Asaumi, K. Ogura and M. Ebisawa	Risk of anaphylaxis during an oral food challenge increases with increasing specific ige levels	2018	Journal of Allergy and Clinical Immunology	INCLUDED	Abstract +author data
Zubrinich, C., J. Douglass, J. Bartlett, M. Patel and M. Hew	Anaphylaxis presentations to the emergency department: impending Victorian reporting legislation	2019	Internal Medicine Journal	INCLUDED	Full Text
Brough HA, Caubet JC, Mazon A, Haddad D, Bergmann MM, Wassenberg J, Panetta V, Gourgey R, Radulovic S, Nieto M, Santos AF, Nieto A, Lack G, Eigenmann PA.	Defining challenge-proven coexistent nut and sesame seed allergy: A prospective multicenter European study	2020	J Allergy Clin Immunol.	INCLUDED	Full Text + author data
Capucilli P, Kennedy K, Lee J, Grundmeier RW, Spergel JM.	Accidental versus new food allergy reactions in a pediatric emergency department	2019	J Allergy Clin Immunol Pract	INCLUDED	Full Text + author data
Capucilli, P., Kennedy K, Alfaro M, Spergel Z, Dorris S, Spergel JM.	Age Differences in Food Reaction Severity During Oral Food Challenges in a Large Pediatric Population	2021	Journal of Allergy and Clinical Immunology	INCLUDED	Full Text + author data
Chatelier, J., Lin, T., Stojanovic, S., Dabscheck, E., Zubrinich, C., Bosco, J., Hew, M.	Risk factors associated with refractory anaphylaxis	2019	Internal Medicine Journal 49 (Supplement 4)	INCLUDED	Abstract
Cohen N, Capua T, Pivko-Levy D, Ben-Shoshan M, Rimon A, Benor S.	Improved diagnosis and treatment of anaphylaxis in a pediatric emergency department (2013-2018)	2019	J Allergy Clin Immunol Pract	INCLUDED	Full Text + author data
Kahveci M, Akarsu A, Koken G, Sahiner UM, Soyer O, Sekerel BE.	Food-induced anaphylaxis in infants, as compared to toddlers and preschool children in Turkey	2020	Pediatr Allergy Immunol	INCLUDED	Full Text
Muramatsu K, Imamura H, Tokutsu K, Fujimoto K, Fushimi K, Matsuda S.	Epidemiological Study of Hospital Admissions for Food-induced anaphylaxis using the Japanese Diagnosis Procedure Combination Database	2020	J Epidemiol	INCLUDED	Full Text
Murata MA, Yamamoto LG.	Patient/parent administered epinephrine in acute anaphylaxis	2020	Am J Emerg Med	INCLUDED	Full Text
Oya S, Kinoshita K, Daya M, Kinoshita H.	Characteristics of Anaphylactic Reactions: A Prospective Observational Study in Japan	2020	J Emerg Med	INCLUDED	Full Text
Trainor JL, Pittsenbarger ZE, Joshi D, Adler MD, Smith B, Gupta RS.	Outcomes and Factors Associated With Prehospital Treatment of Pediatric Anaphylaxis	2020	Pediatr Emerg Care	INCLUDED	Full Text

J. Summary of Data Extraction for Included Studies

Table S15. Individual study characteristics for included references

ID	Reference	Anaphylaxis definition used by study authors	Number of study-defined anaphylaxis events (any trigger)	Number of cardiovascular or lower respiratory reactions (any trigger)	Number of reactions treated with adrenaline	Number of reactions treated with multiple doses of adrenaline	Number of reactions where at least 1 additional dose was adminstered by a healthcare professional	Reaction Setting	Study age group	% male	Study design	Study used for All-trigger meta-analyses	Study used for Food-trigger meta-analyses	Study used for Venom-trigger meta-analyses
1	Dogru et al, 2017	NIAID	66	55	21	0	0	Accidental Reaction	Paediatric	74.2	Retrospective	Yes	Yes	Yes
2	Van Der Valk et al, 2016	NIAID	49	9	8	0	0	Food Challenge	Paediatric	59.2	Prospective	Yes	Yes	.
3	Maris et al, 2015	NIAID	113	113	42	6	5	Accidental Reaction	Paediatric	63.7	Prospective	Yes	Yes	Yes
4	Topal et al, 2014	NIAID	34	25	20	4	4	Accidental Reaction	Paediatric	61.8	Retrospective	Yes	.	.
5	Chung et al, 2014	NIAID	136	76	73	12	12	Accidental Reaction	Both	34.6	Retrospective	Yes	Yes	Yes
6	Mulligan et al, 2014	.	13	.	8	1	1	Food Challenge	Paediatric	.	Retrospective	Yes	Yes	.
7	Hsiao et al, 2014	.	36	.	36	0	0	Food Challenge	Paediatric	.	Retrospective	Yes	Yes	.
8	Alqurashi et al, 2015	NIAID	484	407	309	37	37	Accidental Reaction	Paediatric	64.9	Retrospective	Yes	.	.
9	Farias Aquino et al, 2013	NIAID	203	111	33	9	9	Accidental Reaction	Both	47.3	Retrospective	Yes	Yes	Yes
10	Abrams et al, 2017	> than 1 organ system involvement OR use of Adrenaline	20	4	16	1	1	Food Challenge	Paediatric	59.6	Retrospective	Yes	Yes	.
11	Villafana-Soto et al, 2011	cardiorespiratory symptoms	15	15	7	2	2	Food Challenge	Paediatric	.	Retrospective	Yes	Yes	.
12	Arana et al, 2009	NIAID	47	40	33	2	.	Accidental Reaction	Paediatric	72.0	Retrospective	Yes	.	.
13	Ellis et al, 2007	Canadian Pediatric Surveillance Program: 2 body systems	134	126	106	26	.	Accidental Reaction	Both	51.5	Prospective	Yes	Yes	Yes
14	White et al, 2015	.	852	.	636	54	.	Accidental Reaction	Paediatric	.	Retrospective	Yes	.	.
15	Manivannan et al, 2014	NIAID	63	.	15	1	.	Accidental Reaction	Both	35.0	Retrospective	Yes	.	.
16	Johnson et al, 2014	NIAID	303	246	239	7	.	Accidental Reaction	Paediatric	55.6	Retrospective	Yes	Yes	.
17	Brown et al, 2013	NIAID	315	300	245	76	64	Accidental Reaction	Both	50.0	Prospective	Yes	Yes	Yes
18	Ben-Shoshan et al, 2013	NIAID	168	.	122	10	10	Accidental Reaction	Paediatric	51.8	Retrospective	Yes	.	.
19	Turner et al, 2013	NIAID	12	11	5	1	1	Food Challenge	Paediatric	58.9	Prospective	Yes	Yes	.
20	Lee et al, 2013	NIAID	310	.	262	16	16	Food Challenge	Paediatric	65.8	Retrospective	Yes	Yes	.
21	Huang et al, 2012	NIAID	213	.	169	13	9	Accidental Reaction	Paediatric	51.6	Retrospective	Yes	.	.
22	Rudders et al, 2010	NIAID	40	27	14	3	.	Accidental Reaction	Both	53.6	Retrospective	Yes	.	Yes
23	Jarvinen et al, 2009	use of adrenaline	50	34	50	3	3	Food Challenge	Paediatric	58.2	Retrospective	Yes	Yes	.
24	Manivannan et al, 2009	NIAID	208	103	104	27	27	Accidental Reaction	Both	44.2	Retrospective	Yes	Yes	Yes
25	Arkwright, 2009	use of adrenaline	18	.	22	0	0	Accidental Reaction	Paediatric	60.0	Retrospective	Yes	Yes	.
26	Jarvinen et al, 2008	use of adrenaline	95	.	95	18	17	Accidental Reaction	Paediatric	72.6	Retrospective	Yes	Yes	.
27	De Swert et al, 2008	NIAID	64	47	11	4	.	Accidental Reaction	Paediatric	64.6	Retrospective	Yes	.	.
28	Oren et al, 2007	NIAID	19	12	14	3	.	Accidental Reaction	Both	.	Retrospective	Yes	Yes	.
29	Webb and Lieberman, 2004	use of adrenaline	14	.	14	4	.	Accidental Reaction	Both	37.6	Retrospective	Yes	.	.
30	Uguz et al, 2005	.	50	47	22	4	.	Accidental Reaction	Both	47.7	Retrospective	Yes	Yes	.
31	Rueter et al, 2018	NIAID	251	251	175	20	20	Accidental Reaction	Paediatric	71.7	Retrospective	Yes	Yes	Yes
32	Kondo et al, 2018	.	68	52	48	8	8	Accidental Reaction	Both	77.9	Retrospective	Yes	.	.
33	Goh et al, 2018	NIAID	366	302	312	17	16	Accidental Reaction	Both	50.9	Retrospective	Yes	Yes	Yes
34	Lee et al, 2017	NIAID	872	609	514	79	26	Accidental Reaction	Both	42.0	Both	Yes	.	.
35	Tyquin et al, 2017	ASCIA	153	.	153	20	.	Accidental Reaction	Paediatric	59.0	Retrospective	Yes	Yes	Yes
36	Yanagida et al, 2017	NIAID	190	165	90	20	20	Food Challenge	Both	68.0	Retrospective	Yes	Yes	.
37	Noone et al, 2015	NCI-CTCAE	42	42	29	2	2	Food Challenge	Both	67.6	Retrospective	Yes	Yes	.
38	Capps and Arkwright, 2010	cardiorespiratory symptoms	514	475	116	4	4	Accidental Reaction	Both	39.6	Retrospective	Yes	Yes	Yes
39	Tiyyagura et al, 2014	NIAID	218	149	126	5	.	Accidental Reaction	Paediatric	56.0	Retrospective	Yes	.	.
40	Campbell et al, 2015	NIAID	582	492	326	45	.	Accidental Reaction	Both	42.0	Both	Yes	.	.
41	Grabenhenrich et al, 2018	.	8187	8006	1669	174	172	Accidental Reaction	Both	48.8	Prospective	Yes	Yes	Yes
42	Vijaykumar et al, 2017	.	24	.	24	3	3	Food Challenge	Paediatric	70.8	Retrospective	Yes	Yes	.
43	Tsuang et al, 2018	.	221	.	221	24	18	Accidental Reaction	Paediatric	59.0	Retrospective	Yes	Yes	.
44	Tsuang et al, 2018	.	14	.	14	1	.	Accidental Reaction	Paediatric	63.0	Prospective	Yes	Yes	.

ID	Reference	Anaphylaxis definition used by study authors	Number of study-defined anaphylaxis events (any trigger)	Number of cardiovascular or lower respiratory reactions (any trigger)	Number of reactions treated with adrenaline	Number of reactions treated with multiple doses of adrenaline	Number of reactions where at least 1 additional dose was adminstered by a healthcare professional	Reaction Setting	Study age group	% male	Study design	Study used for All-trigger meta-analyses	Study used for Food-trigger meta-analyses	Study used for Venom-trigger meta-analyses
45	Kim et al, 2018	NIAID	185	.	114	25	25	Accidental Reaction	Adult	45.0	Retrospective	Yes	.	.
46	Dribin et al, 2019	NIAID	665	.	339	81	.	Accidental Reaction	Both	56.7	Retrospective	Yes	.	.
47	Soller et al, 2019	NIAID	55	18	53	9	9	Food Challenge	Paediatric	64.0	Prospective	Yes	Yes	.
48	Hamilton et al, 2019	CoFAR grading of allergic reactions	47	.	47	9	9	Food Challenge	Paediatric	63.3	Retrospective	Yes	Yes	.
49	Anvari S et al, 2019	NIAID	275	180	243	25	.	Accidental Reaction	Paediatric	53.3	Retrospective	Yes	Yes	.
50	Giclas et al, 2019	Brown, NIAID/FAAN, and Niggemann & Beyer	59	44	52	4	4	Food Challenge	Paediatric	63.0	Retrospective	Yes	Yes	.
51	Elizur et al, 2018	2 systems	93	31	41	1	1	Food Challenge	Both	55.4	Prospective	Yes	Yes	.
52	Sundquist et al, 2019	NIAID	267	222	142	23	19	Accidental Reaction	Both	42.7	Retrospective	Yes	Yes	Yes
53	Coutinho et al, 2018	NIAID	32	25	25	3	3	Accidental Reaction	Both	56.3	Retrospective	Yes	Yes	Yes
54	Ponce Guevara et al, 2018	NIAID	89	72	37	7	7	Accidental Reaction	Both	50.6	Retrospective	Yes	.	.
55	Nagakura et al, 2018	NIAID	25	8	9	0	.	Food Challenge	Paediatric	72.0	Prospective	Yes	Yes	.
56	Yanagida et al, 2018	NIAID	334	.	45	2	2	Food Challenge	Both	67.3	Prospective	Yes	Yes	.
57	Asaumi et al, 2016	.	30	9	6	0	.	Food Challenge	Paediatric	65.0	Retrospective	Yes	Yes	.
58	Nogic et al, 2016	ASCIA	52	51	49	9	9	Accidental Reaction	Paediatric	62.0	Retrospective	Yes	.	.
59	Lee and Stukus, 2015	NIAID	408	311	330	21	21	Accidental Reaction	Paediatric	62.0	Retrospective	Yes	Yes	.
60	Inoue and Yamamoto, 2013	NIAID	61	56	35	3	3	Accidental Reaction	Paediatric	65.6	Retrospective	Yes	Yes	.
61	Itazawa et al, 2019	Not defined Sampson severity used	531	895	160	10	10	Food Challenge	Paediatric	65.2	Prospective	Yes	Yes	.
62	Brennan et al, 2013	NIAID	22	13	22	2	2	Food Challenge	Paediatric	81.2	Retrospective	Yes	Yes	.
63	Noimark et al, 2012	NIAID	245	150	41	13	9	Accidental Reaction	Paediatric	58.7	Retrospective	Yes	.	.
64	Dibs and Baker, 1997	cardiorespiratory symptoms	55	51	46	7	.	Accidental Reaction	Both	56.0	Retrospective	Yes	.	.
65	Cardona et al, 2017	use of adrenaline	268	260	268	43	40	Accidental Reaction	Both	48.5	Retrospective	Yes	Yes	Yes
66	Coutinho et al, 2019	NIAID	43	41	38	8	8	Accidental Reaction	Adult	53.5	Retrospective	Yes	Yes	.
67	Ewan and Clark, 2005	cardiorespiratory symptoms	27	27	3	0	0	Accidental Reaction	Paediatric	58.0	Prospective	Yes	Yes	.
68	Ewan and Clark, 2008	cardiorespiratory symptoms	269	269	36	0	0	Accidental Reaction	Paediatric	.	Retrospective	Yes	Yes	.
69	Ewan and Clark, 2008	cardiorespiratory symptoms	22	22	3	0	0	Accidental Reaction	Paediatric	.	Prospective	Yes	Yes	.
70	Ewan and Clark, 2001	cardiorespiratory symptoms	26	26	22	0	0	Accidental Reaction	Both	50.0	Prospective	Yes	Yes	.
71	Gold and Sainsbury, 2000	cardiorespiratory symptoms	45	45	13	2	2	Accidental Reaction	Paediatric	.	Retrospective	Yes	.	.
72	Zubrinich et al, 2019	cardiorespiratory symptoms	56	34	44	13	13	Accidental Reaction	Both	30.0	Retrospective	Yes	Yes	.
73	Liu et al, 2019	NIAID	430	357	305	50	50	Accidental Reaction	Both	42.0	Prospective	Yes	Yes	Yes
74	Olabarri et al, 2019	.	453	.	428	53	53	Accidental Reaction	Paediatric	.	Prospective	Yes	.	.
75	Mehr et al, 2009	NIAID	109	109	64	15	15	Accidental Reaction	Paediatric	59.8	Retrospective	Yes	.	.
76	Rudders et al, 2012	.	321	262	321	54	.	Accidental Reaction	Paediatric	47.0	Retrospective	Yes	Yes	Yes
77	Banerji et al, 2010	.	295	.	91	15	14	Accidental Reaction	Adult	38.0	Retrospective	Yes	Yes	.
78	Gabrielli et al, 2019	NIAID	3498	1669	2558	282	223	Accidental Reaction	Both	55.6	Prospective	Yes	Yes	Yes
79	Brough et al, 2020	Sampson	39	28	43	6	6	Food Challenge	Paediatric	69.0	Prospective	Yes	Yes	.
80	Capucilli et al, 2019	Multisystem	141	33	142	14	14	Accidental Reaction	Paediatric	50.0	Retrospective	Yes	Yes	.
81	Capucilli et al, 2021	cardiorespiratory or use of adrenaline	440	143	440	69	69	Food Challenge	Paediatric	.	Retrospective	Yes	Yes	.
82	Chatelier et al, 2019	NIAID	174	.	163	77	.	Accidental Reaction	Both	44.2	Retrospective	Yes	.	.
83	Cohen et al, 2019	NIAID	375	67	238	11	8	Accidental Reaction	Paediatric	61.0	Retrospective	Yes	Yes	Yes
84	Kahveci et al, 2020	NIAID	175	103	71	1	.	Accidental Reaction	Paediatric	67.5	Retrospective	Yes	Yes	.
85	Muramatsu et al, 2020	ICD	9078	.	4192	243	.	Accidental Reaction	Both	57.0	Retrospective	Yes	.	.
86	Murata et al,2020	use of adrenaline	181	.	181	24	.	Accidental Reaction	Both	55.8	Retrospective	Yes	.	.
87	Oya et al, 2020	NIAID	302	248	207	7	.	Accidental Reaction	Both	39.7	Prospective	Yes	.	.
88	Trainor et al, 2020	NIAID	414	199	163	48	48	Accidental Reaction	Paediatric	54.3	Retrospective	Yes	.	.

End of Online Supplement
